# Light-Fueled Primitive Replication and Selection in Biomimetic Chemical Systems

**DOI:** 10.1021/jacs.3c03597

**Published:** 2023-06-07

**Authors:** Éva Bartus, Attila Tököli, Beáta Mag, Áron Bajcsi, Gábor Kecskeméti, Edit Wéber, Zoltán Kele, Gabriel Fenteany, Tamás A. Martinek

**Affiliations:** † Department of Medical Chemistry, University of Szeged, Dóm tér 8, H-6720 Szeged, Hungary; ‡ ELKH-SZTE Biomimetic Systems Research Group, University of Szeged, Dóm tér 8, H-6720 Szeged, Hungary; § Institute of Genetics, Biological Research Centre, Temesvári krt. 62, H-6726 Szeged, Hungary

## Abstract

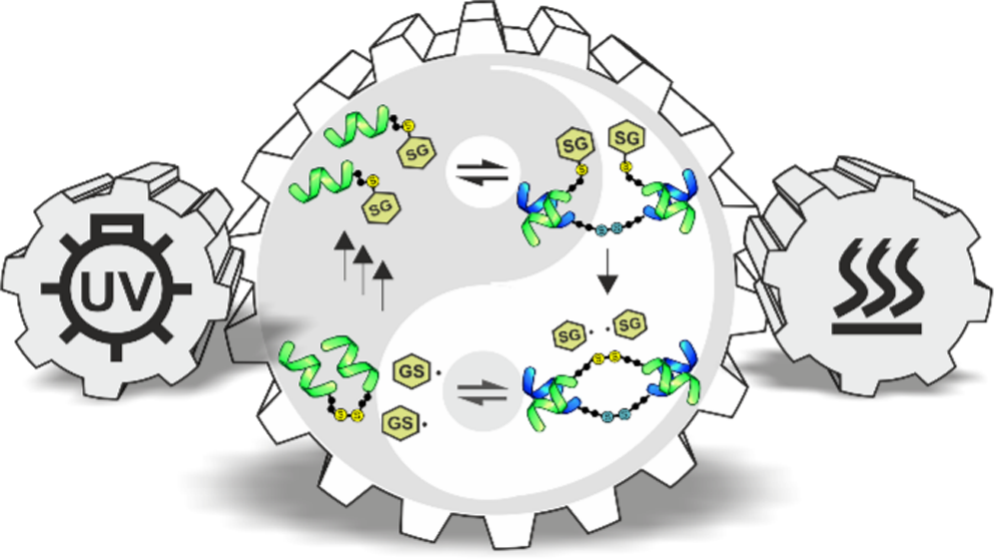

The concept of chemically evolvable replicators is central to abiogenesis. Chemical evolvability requires three essential components: energy-harvesting mechanisms for nonequilibrium dissipation, kinetically asymmetric replication and decomposition pathways, and structure-dependent selective templating in the autocatalytic cycles. We observed a UVA light-fueled chemical system displaying sequence-dependent replication and replicator decomposition. The system was constructed with primitive peptidic foldamer components. The photocatalytic formation–recombination cycle of thiyl radicals was coupled with the molecular recognition steps in the replication cycles. Thiyl radical-mediated chain reaction was responsible for the replicator death mechanism. The competing and kinetically asymmetric replication and decomposition processes led to light intensity-dependent selection far from equilibrium. Here, we show that this system can dynamically adapt to energy influx and seeding. The results highlight that mimicking chemical evolution is feasible with primitive building blocks and simple chemical reactions.

## Introduction

Chemical systems mimicking evolution need to operate dynamically far from equilibrium,^1^ which is essential for their open-ended adaptive behavior. The dynamic kinetic stability in such systems results from the balance between the asymmetric formation and destruction processes driven by the dissipation of energy harvested from the environment.^2−4^ The dissipative chemical systems^2,5,6^ are generated by nonequilibrium dynamics, which rely on an energy-harvesting catalytic cycle covering the entropy production of the system.

Chemical^7−10^ or light energy^11,12^ can promote chemical replication by producing precursors in a protometabolic manner. Molecular complexification^13^ and selection phenomena^14^ have been demonstrated by driving replication and decomposition with chemical energy in a kinetically asymmetric manner. However, a current challenge is simultaneously driving replication and replicator decomposition with external light energy. Without an efficiently competing decomposition pathway, the autocatalytic replicator synthesis runs into precursor depletion or product inhibition, depriving the system of adaptivity. If the opposite processes are kinetically symmetric, the external light energy has no influence on the steady-state concentrations, and it drives the system to equilibrium with strongly limited adaptivity. Therefore, light-driven dissipative replicator systems require asymmetric replication and decomposition mechanisms. Our goal was to find a feasible light energy-harvesting covalent chemistry that drives synthetic and breakdown processes asymmetrically, depending on the light intensity.

**Figure 1:**
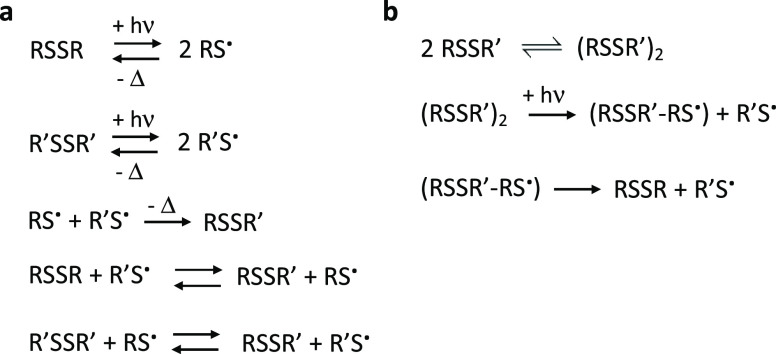
Photocatalytic thiyl-mediated disulfide exchange reaction mechanisms. (a) Diffusion-controlled chain reaction described in the literature.^15^ (b) Proximity-controlled intracomplex exchange hypothesized in this work.

UV-induced photochemical rearrangement reactions can be carried out with aliphatic disulfides in the organic liquid phase^15,16^ and an aqueous medium.^17−19^ These works showed that both UVA and UVB induce photolysis, producing thiyl radicals despite the strongly reduced absorbance of the disulfide group in the UVA region.^16,19^ Thiyl radicals participate in a diffusion-controlled chain reaction, and depending on the steric accessibility, the exchange proceeds through radical substitution or recombination (Figure 1a).

Anomalously high reaction rates were obtained for the bulky *iso*-butyl substituent,^15^ indicating that the reaction is not always governed by the diffusion-controlled collision of the thiyl radicals. These seminal findings suggest that photochemical disulfide rearrangement facilitates both diffusion-controlled and proximity-controlled mechanisms in a substituent-dependent manner (Figure 1b). Since these kinetically asymmetric mechanisms are of different order functions of light intensity (see below), they promise a way to a light energy-dependent nonequilibrium dynamics forming a photocatalytic dissipative chemical system. These properties are desirable for adaptivity and evolvability.

We set out to test the photochemical disulfide rearrangement with a diverse population of helical foldamers as substituents in the aqueous medium. In order to avoid the rapid decomposition of the peptidic chains, the feasibility of the light-induced disulfide rearrangement was investigated at UVA wavelengths. We hypothesized that preferential binding between the foldamer chains could exert side chain-dependent proximity control over the photochemical exchange. In contrast, noninteracting segments should readily enter the diffusion-governed chain reaction. The competition between the two processes was expected to yield a light intensity-dependent composition without damaging the peptidic chains. Seeking potential autocatalytic phenomena, we aimed to investigate the influence of the foldamer–foldamer interactions on the energy-harvesting thiyl chemistry. Designed helical peptides can perform selective templating in autocatalytic processes,^20,21^ and exponential replication has been achieved for peptidic helices.^22^

Here, we show that UVA light-fueled disulfide rearrangement can drive a chemical system to off-equilibrium states in a dissipative manner. We found that sequence-dependent auto-/cross-catalysis plays a dominant role in the exchange processes. A competing breakdown mechanism influenced the replicator concentration, laying the foundation for the adaptive selection phenomenon.

## Results

### UVA-Induced Photochemical Disulfide Rearrangement Is Feasible in a Foldamer Library

**Figure 2:**
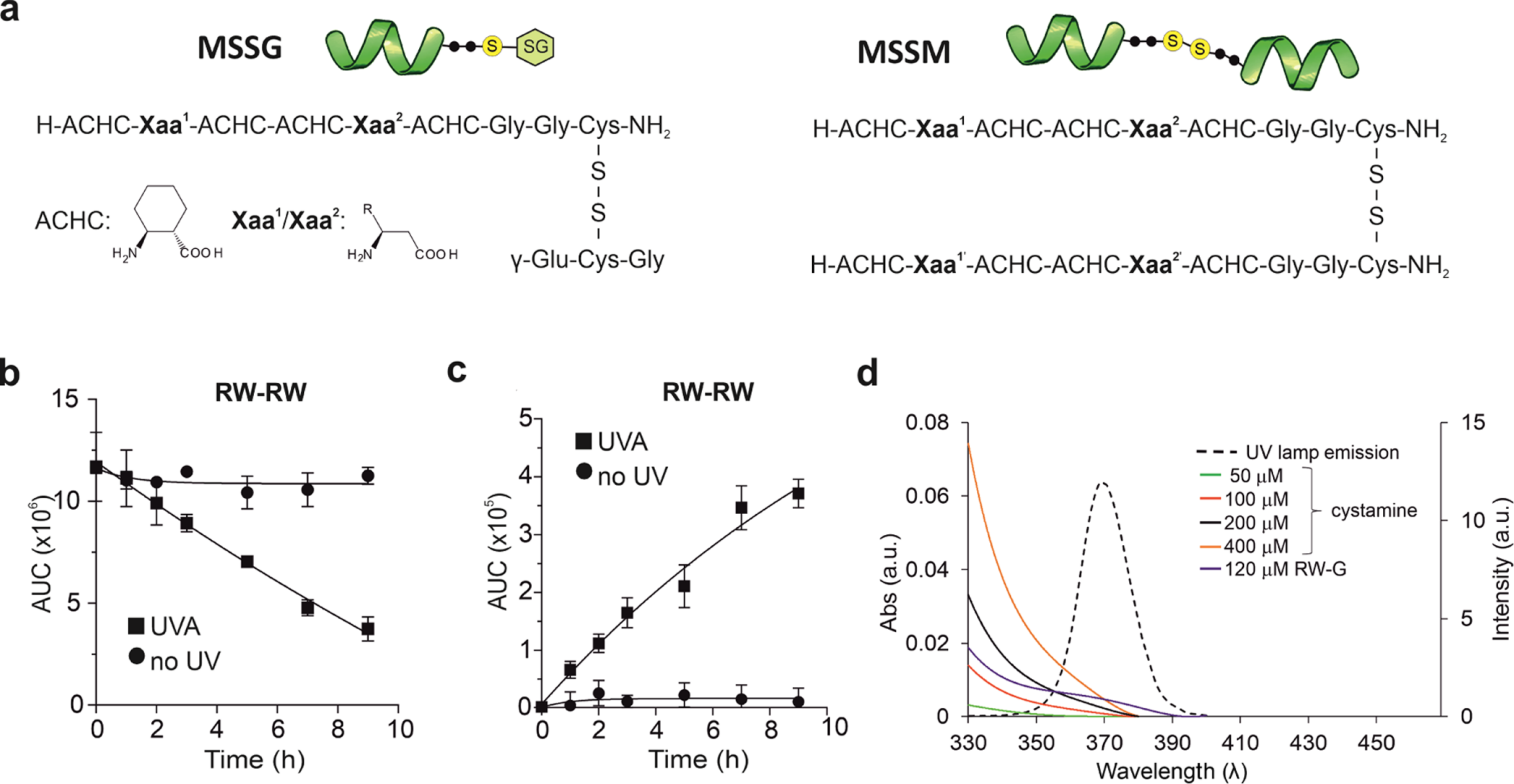
General sequences of the foldamer disulfide components and their synthesis and breakdown in the photochemical disulfide exchange reaction. (a) General sequences. SG indicates glutathione (γ-L-glutamyl-L-cysteinyl-glycine), and ACHC stands for 1*S*,2*S*-2-aminocyclohexanecarboxylic acid, which promotes helical folding. In the highlighted positions Xaa^1^ and Xaa^2^, β^3^-amino acids with proteinogenic side chains were incorporated in the following combinations: **IF, KW, LW, QW, RW, RF, SW, TW, VW, WF, WW**, and **YF**. One-letter codes correspond to the side chains in the standard α-amino acid notation. (b) Concentration decay of the **RW-RW** dimer as a result of UVA irradiation at 365 nm (square) and without UVA illumination (circle). Exponential curves were fitted to the data points to guide the eye. This reaction was started with a system containing all 10 disulfide dimers formed by combining the subset **RW, WF, LW**, and **TW**. Data for all 10 dimers are given in Figure S3. Dimer concentrations were 1 μM for each combination. The system also contained 100 μM oxidized glutathione. In parallel, the amount of monomer (**RW-G**) increases at the expense of **RW**-containing dimers without the formation of UV-induced degradation products (Figure S7). (c) Time-dependent concentration increase of the **RW**-**RW** dimer as a result of UVA irradiation at 356 nm (square) and without UVA (circle). Exponential curves were fitted to the data points to guide the eye. This experiment was started with pure disulfide monomers of **RW-G, WF-G, LW-G**, and **TW-G**. Data for all 10 dimers are given in Figure S4. (d) UV spectra of cystamine solutions in the range of 330–400 nm. UV spectrum of the representative monomer **RW-G** at the concentration of 120 μM. The emission spectrum of the UV lamp (UVL-28 EL Series UV Lamp) in the range of 330–400 nm (dashed curve). The solvent conditions for the photocatalytic reactions were pH = 7.0, 20 mM HEPES, 150 mM NaCl, 2 mM CaCl_2_.

We constructed the foldamer library using hexameric β-peptide foldamers designed to fold into compact helical structures^23,24^ (Figure 2a). The biomimetic structure and recognition surface of helical foldamers can be readily controlled using cyclic side chains and a repeating stereochemical pattern of the backbone.^25,26^ The structure was stabilized with *trans*-2-aminocyclohexanecarboxylic acids (ACHC), and the sequences contained two β^3^-amino acids with proteinogenic side chains at the variable positions (Xaa^1^ and Xaa^2^). Cys residues were attached to the C-terminus, allowing disulfide linkage between two foldamer segments yielding dimers (Figure 2a). We define the disulfides containing only one foldamer and glutathione as monomers. The ordered secondary structure (Figure S1) can promote self-association.^27^ This property was confirmed by the sequence-dependent association tendency of the foldamer segments in an aqueous glutathione redox buffer relaxed to equilibrium with the dynamic covalent thiolate mechanism (Figure S2). We did not pursue other structural designs to keep the complexity of the sequences at a low level.

For the photochemical exchange experiments, free thiol groups were eliminated, and the pH was set to 7.0 to block the light-independent thiolate-mediated nucleophilic disulfide exchange. It was essential to avoid the rapid degradation of the peptidic chains; therefore, the disulfide rearrangement reactions were attempted with UVA irradiation (365 nm) at a constant temperature (303 K) according to literature protocols.^19^ First, we tested the UVA-induced breakdown reaction of pure dimers (1 μM each) to monomers in the presence of oxidized glutathione (100 μM). In this step, the starting dimers were synthesized by using a limited set of foldameric segments: **RW, WF, LW**, and **TW**, allowing 10 different combinations. One-letter codes correspond to the variable side chains in the standard α-amino acid notation. The UVA illumination at 5.1 mW cm^–2^ decreased the dimer concentrations, while monomers were produced in a time-dependent manner (Figures 2 and S3). Second, we started the reaction from a mixture of pure monomers **RW-G**, **WF-G, LW-G**, and **TW-G**. The results confirmed the UVA-induced synthesis of dimers and the parallel decay of the monomer concentration (Figures 2 and S4). The reactions carried out under argon atmosphere successfully prevented the UV-induced degradation of the peptides including the oxidation of the tryptophan side chains (Figures S5–S7). We found no conversion without UVA illumination, and the radical scavenger nitric oxide blocked the photocatalytic reaction. These observations confirmed the literature findings^16,19^ that the exchange reaction could be induced with UVA light centered at 365 nm and proceeds via a radical mechanism. Starting the net conversions from pure monomers and dimers supported that UVA illumination facilitated the underlying mechanisms for photocatalytic dimer synthesis and breakdown, respectively.

However, this phenomenon apparently contrasts with the absorbance maximum of the disulfide bond at around 280 nm.^28^ A recent work showed that conformation-dependent stereoelectronic effects around the S–S bond could shift the absorption to the UVA region.^29^ To test this phenomenon, we measured a simple disulfide model, cystamine, which displayed a concentration-dependent absorption tail in the UVA region (Figure 2d). A representative monomer **RW-G** was also tested, and the absorption tail was observed in the 330–400 nm range. This overlap with the UV lamp′s emission spectrum facilitates the excitation of the disulfide chromophores in the UVA range. A fast conformational dynamics around the disulfide bond can expose the whole population to a UVA-induced cleavage within a short time window. In line with the literature, these findings strongly supported the ability of the disulfide group to absorb light energy at around 365 nm and, thus, drive the photochemical rearrangement.

### Light Intensity- and Sequence-Dependent Kinetic Asymmetry in the Photochemical Foldamer Disulfide Exchange

**Figure 3:**
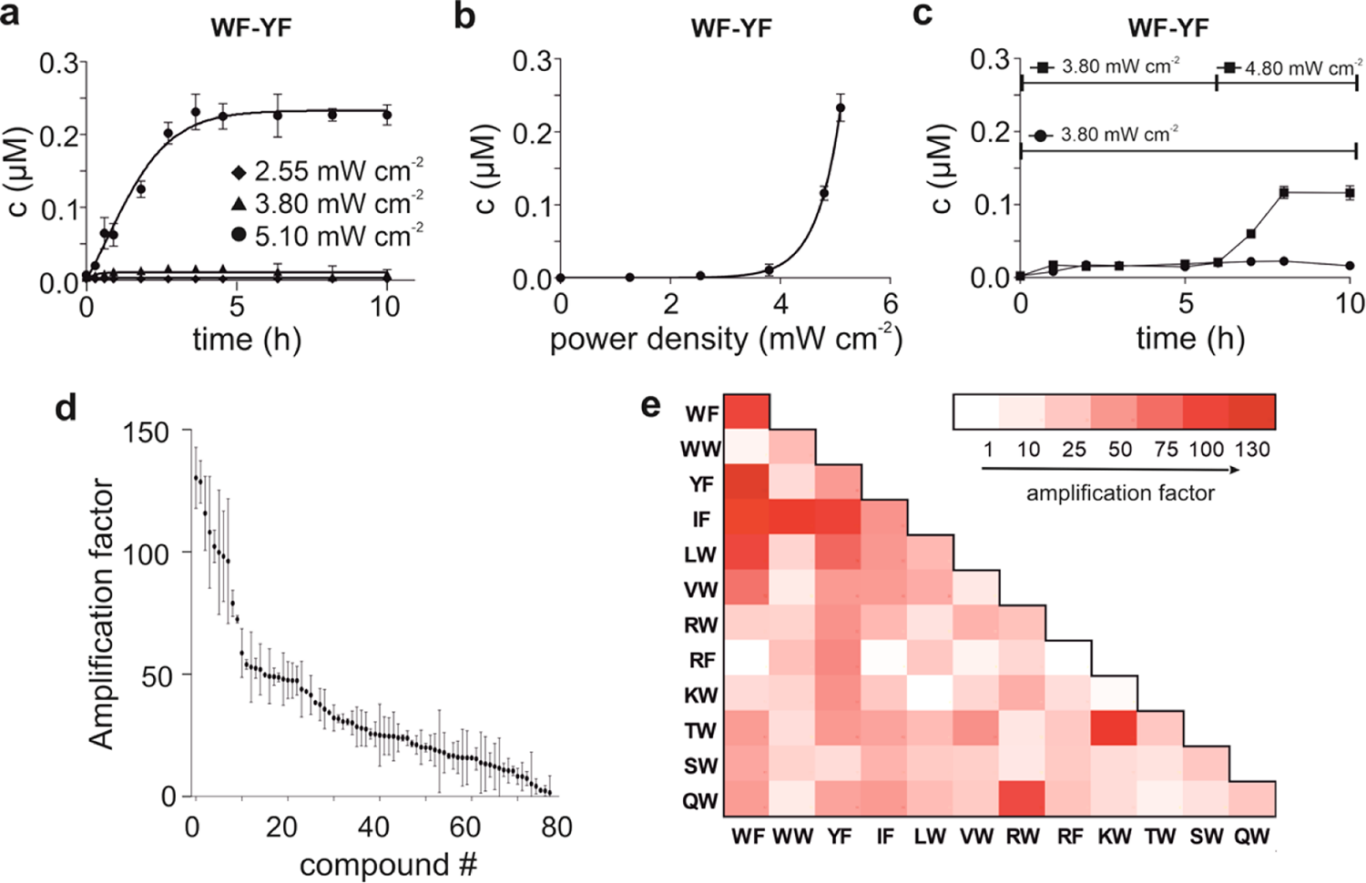
Light intensity- and substituent-dependent dimer concentrations. (a) Time- and light intensity-dependent concentration of a representative dimer (**WF-YF**) obtained at power densities of 2.55 mW cm^–2^ (diamonds), 3.80 mW cm^–2^ (triangles), and 5.10 mW cm^–2^ (3 circles). (b) Light intensity-dependent steady-state concentrations for **WF-YF** (circles). (c) Adaptivity test started with a UVA intensity of 80 mW cm^–2^ and subsequent increase to 4.80 mW cm^–2^ (squares), and the control measurement run with a constant intensity of 3.80 mW cm^–2^ (circles). (d) Light intensity-dependent amplifications obtained upon increasing power density from 2.55 to 5.10 mW cm^–2^. (e) Heat map representation of the light intensity- and sequence-dependent amplifications obtained upon increasing power density from 2.55 to 5.10 mW cm^–2^. The solvent conditions for the photocatalytic reactions were 20 mM HEPES (pH = 7.0), 150 mM NaCl, 2 mM CaCl_2_. The samples contained only disulfide derivatives (Figure 2a), and thiols were not present to block the thiolate-mediated exchange mechanism.

Next, we tested the photocatalytic disulfide exchange on a larger library of pure monomers. We chose 12 different side chain combinations based on their tendency to self-associate and the ability to bind to hydrophobic patches:^30^**IF**, **KW**, **LW**, **QW**, **RW**, **RF**, **SW**, **TW**, **VW**, **WF**, **WW**, and **YF**. The power density was increased linearly in four steps up to the maximum value available in our setup (5.10 mW cm^–2^). We could not detect dimer formation without irradiation and at 25% light intensity. At 50% and above, dimers were observed (Table 1), and the system attained steady states at low conversions (without monomer depletion) in 5 h (Figures 3a and S8). The increasing light intensity caused the onset of a steep increase in the steady-state dimer population only above 4.0 mW cm^–2^ (Figure 3b). This unexpected light intensity–concentration relationship could be mathematically approximated with a hyperbolic curve. We carried out a separate light intensity-dependent experiment, where the UVA intensity of 3.80 mW cm^–2^ was applied first, and after reaching the corresponding stationary state, the intensity was increased to 4.80 mW cm^–2^ for the same sample (Figure 3c). The dimer population again increased, and a new steady state was attained adaptively.

**Table 1:** Analytical Data of Foldameric Sequences Present in the UV-Induced Disulfide Exchange Reactions

			detected ions					detected ions	
compounds	calculated molar mass (Da)	retention time (min)^a^	[M + 2H^+^]^2+^	[M + 3H^+^]^3+^	compounds	calculated molar mass (Da)	retention time (min)^a^	[M + 2H^+^]^2+^	[M + 3H^+^]^3+^
**IF-IF**	2044.66	17.73	1022.95	682.29	**TW-SW**	2083.70	14.13	1042.93	695.92
**IF-QW**	2098.67	15.70	1049.79	700.47	**TW-TW**	2098.64	14.35	1049.99	700.56
**IF-SW**	2057.62	15.79	1029.89	686.75	**VW-QW**	2122.74	15.35	1063.00	708.79
**IF-TW**	2071.65	16.05	1036.42	691.59	**VW-SW**	2082.63	15.44	1042.40	695.14
**IF-VW**	2069.67	17.29	1035.41	690.82	**VW-TW**	2096.66	15.70	1049.37	699.79
**KW-IF**	2098.71	14.55	1049.79	700.00	**VW-VW**	2094.68	16.97	1048.03	698.88
**KW-KW**	2152.76	11.70	1077.4	718.48	**WF-IF**	2117.72	17.27	1059.49	706.71
**KW-LW**	2137.75	14.70	1069.44	713.56	**WF-KW**	2171.77	14.31	1086.48	724.64
**KW-QW**	2152.72	12.73	1076.93	718.9	**WF-LW**	2156.76	17.45	1078.90	719.64
**KW-RF**	2141.74	11.63	1071.41	714.82	**WF-QW**	2171.73	15.40	1086.93	724.91
**KW-SW**	2111.63	12.73	1056.87	705.04	**WF-RF**	2160.75	14.37	1081.34	721.03
**KW-TW**	2125.70	13.09	1063.35	709.34	**WF-RW**	2199.79	14.48	1100.43	734.51
**KW-VW**	2123.68	14.26	1063.34	708.73	**WF-SW**	2130.68	15.49	1065.95	711.30
**LW-IF**	2084.61	17.84	1042.52	695.41	**WF-TW**	2144.71	15.76	1073.20	715.93
**LW-LW**	2123.72	18.00	1062.03	708.28	**WF-VW**	2142.73	16.96	1071.97	715.50
**LW-QW**	2137.71	15.84	1069.41	713.77	**WF-WF**	2190.78	16.96	1095.93	731.76
**LW-SW**	2096.66	15.94	1048.90	700.19	**WF-WW**	2229.81	16.41	1115.44	744.44
**LW-TW**	2111.67	16.19	1056.01	704.69	**WF-YF**	2167.74	16.10	1084.52	723.76
**LW-VW**	2108.71	17.43	1054.60	703.40	**WW-IF**	2156.75	16.78	1078.99	719.84
**QW-QW**	2152.68	13.71	1077.12	718.79	**WW-KW**	2210.80	13.73	1105.93	738.15
**RF-IF**	2087.69	14.59	1044.38	697.05	**WW-LW**	2195.79	16.89	1098.46	732.71
**RF-LW**	2126.73	14.77	1063.94	709.58	**WW-QW**	2210.76	14.71	1105.98	737.87
**RF-QW**	2141.70	12.73	1071.37	715.15	**WW-RF**	2199.78	14.48	1100.43	734.51
**RF-RF**	2130.72	11.64	1066.67	710.97	**WW-RW**	2238.82	13.94	1120.36	747.47
**RF-SW**	2100.65	12.80	1050.89	701.43	**WW-SW**	2169.71	16.08	1086.53	725.38
**RF-TW**	2114.68	12.77	1057.93	706.26	**WW-TW**	2183.74	15.09	1092.55	728.13
**RF-VW**	2112.70	14.28	1056.99	705.03	**WW-VW**	2181.76	16.39	1091.41	728.06
**RW-IF**	2126.73	14.77	1064.57	709.87	**WW-WW**	2268.84	15.81	1135.45	757.47
**RW-KW**	2180.78	11.80	1091.33	728.04	**WW-YF**	2206.77	15.53	1103.89	736.72
**RW-LW**	2165.77	14.91	1083.50	722.76	**YF-IF**	2094.68	16.41	1047.94	699.11
**RW-QW**	2180.74	12.90	1090.87	728.20	**YF-KW**	2148.73	13.54	1075.00	717.42
**RW-RF**	2169.76	12.22	1085.36	724.15	**YF-LW**	2133.72	16.57	1067.86	712.64
**RW-RW**	2208.80	11.97	1104.90	737.47	**YF-QW**	2148.69	14.50	1074.93	717.02
**RW-SW**	2139.69	12.96	1070.38	714.39	**YF-RF**	2137.71	13.54	1069.47	713.51
**RW-TW**	2153.72	13.29	1077.46	718.90	**YF-RW**	2176.75	13.71	1088.93	726.82
**RW-VW**	2151.74	14.41	1076.35	718.45	**YF-SW**	2107.64	14.58	1054.34	703.55
**SW-QW**	2110.69	13.76	1056.41	704.63	**YF-TW**	2121.67	14.84	1061.45	707.98
**SW-SW**	2070.58	12.99	1035.86	690.84	**YF-VW**	2119.69	16.06	1060.47	707.47
**TW-QW**	2125.66	14.05	1063.25	709.33	**YF-YF**	2144.70	15.22	1073.30	715.89

a Analytical HPLC-MS measurement. Column: Aeris Widepore XB-C18 (250 × 4.6 mm^2^). Method: 5–80% B during 25 min, flow rate: 0.7 mL min^–1^, where eluent A: 0.1% HCOOH in water, eluent B: 0.1% HCOOH in acetonitrile.

We calculated the light-induced amplifications for the dimers upon elevating the power density from 2.55 mW cm^–2^ (50%) to 5.10 mW cm^–2^ (100%) (Figure 3d). We found highly sequence-dependent amplification. The difference between the best and the worst amplified dimers was more than two magnitudes. Among the best-amplified sequences, aromatic and aliphatic hydrophobic side chain enrichment was detected (e.g., **WF-YF**, **WF-IF**, and **WW-IF**) (Figure 3e). Dimers containing polar and cationic residues displayed moderate or low amplification except for the well-amplified dimers **RW-QW** and **KW-TW**.

**Figure 4:**
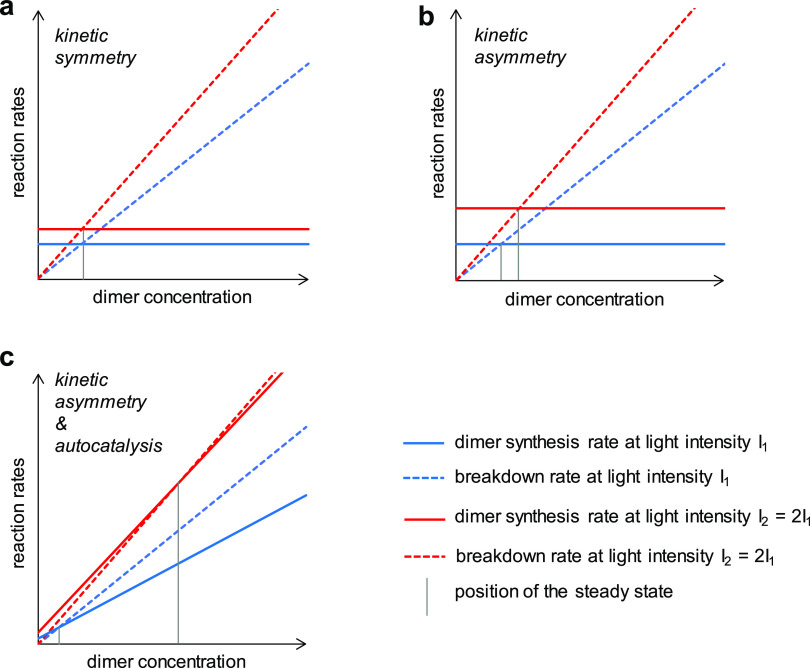
Modeled effects of kinetic asymmetry and autocatalysis on the light-induced steady-state dimer concentrations. A steady-state (gray vertical line) is attained when the dimer synthesis rate (solid) equals the breakdown rate (dashed). We plotted the rate laws at an arbitrary light intensity I_1_ (blue) and I_2_ = 2I_1_ (red). (a) For kinetic symmetry, both dimer synthesis and breakdown rates have the same dependence on the light intensity canceling out its effect on the steady state. We used the reaction order in light intensity of 0.5 for the breakdown and the synthesis reactions, corresponding to the thiyl radical-mediated chain reaction (eqs 1 and 2). (b) For kinetic asymmetry, the dimer synthesis mechanism exhibits a higher-order dependence on light intensity (eq 3), making the steady-state dimer concentrations light intensity-dependent. (c) Autocatalysis introduces a dimer concentration-dependent synthesis rate (eq 5), leading to a hyperbolic increase in the dimer population at an elevated light intensity.

The light intensity dependence of the steady-state dimer concentrations allows conclusions about the relationship between the underlying dimer synthesis and breakdown mechanisms. In kinetic symmetry, the rate laws of the synthesis and breakdown mechanisms are the same order in light intensity (Figure 4a). Thus, the identical light sensitivity of the opposite mechanisms would cancel out, causing light intensity-independent steady states. Therefore, the light intensity dependence of the steady-state dimer concentrations provides evidence for the competing dimer breakdown and synthesis mechanisms, which are different order functions of the light intensity (Figure 4b). This is possible only if the underlying breakdown and the synthesis mechanisms proceed via different light-induced mechanisms. Thus, an energy influx-dependent kinetic asymmetry determines the behavior of the system. The positive correlation between light intensity and the dimer concentrations indicates that the dimer synthesis mechanism is more sensitive to light intensity (higher-order function of light intensity) than the breakdown mechanism. We can also conclude that the kinetic asymmetry is sequence-dependent.

### Sequence-Dependent Auto- and Cross-Catalysis Are Present in the System

**Figure 5:**
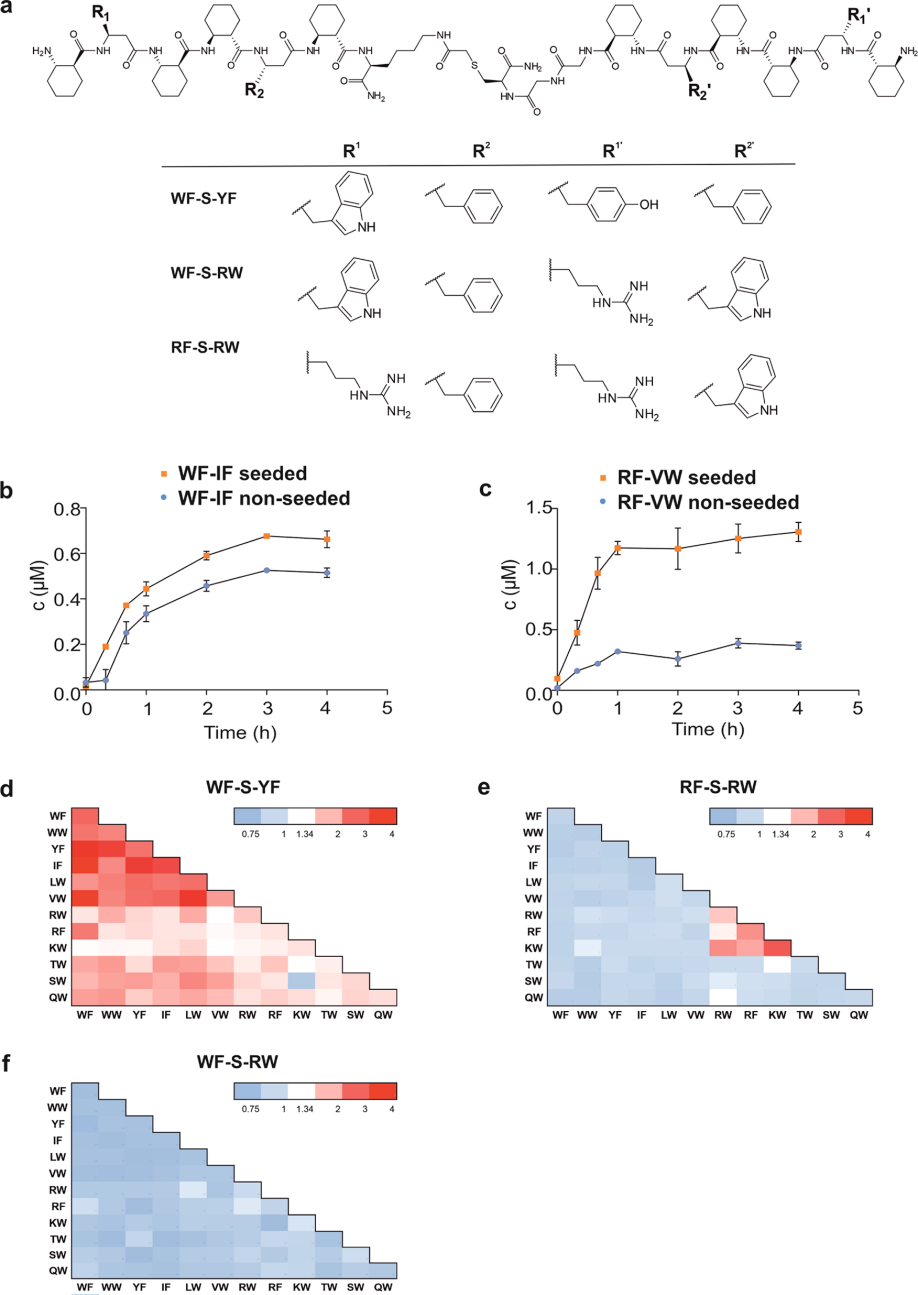
Catalytic effects of seeding with the light-insensitive seeding dimers **WF-S-YF**, **RF-S-RW**, and **WF-S-RW**. (a) Structures of the seeding dimers with the light-insensitive thioether linkage. Seeding experiments were started with 10 μM seed and disulfide monomers at the 5.10 mW cm^–2^ light intensity. Time evolution of the concentrations for representative dimers **WF-IF** (b) and **RF-VW** (c) with (orange square) and without (blue circle) the seeding dimer **WF-S-YF**. The lines are a guide to the eye. **WF-IF** displayed high light-induced amplification in the unseeded measurements, which was strongly enhanced by seeding. **RF-VW** had an unseeded initial time lag, which disappeared upon seeding. (d) Heat map representations of the sequence-dependent initial rate increase (v_seeded_/v_control_) upon seeding with **WF-S-YF** (d), **RF-S-RW** (e), and **WF-S-RW** (f) (Table S1). Red and blue colors indicate above- and below-average initial rate increase, respectively, where the average of 1.34 was calculated from all three seeding experiments. The solvent conditions for the photocatalytic reactions were 20 mM HEPES (pH = 7.0), 150 mM NaCl, 2 mM CaCl_2_.

While kinetic asymmetry can explain the positive correlation between the dimer population and the light intensity, it does not account for the hyperbolic relationship, justifying further investigations. Preliminary modeling of the effects of autocatalysis pointed to a hyperbolic increase of the dimer concentration at elevated light intensities (Figure 4c). Moreover, we observed a time delay in the light-induced synthesis for certain dimers (Figures S4 and 5b), which suggested the presence of autocatalysis in the system. If autocatalysis is coupled to the light-driven synthesis routes, it can be tested in the time domain with a seeding experiment. An initial synthesis rate increase would indicate the catalytic effect, even if the autocatalytic time delay is reduced by an effective spontaneous (non-autocatalytic) synthesis. However, the breakdown of a potential seeding disulfide dimer immediately starts upon UVA irradiation, which we tested for **WF**-**YF** (Figure S9). Thus, despite its potential catalytic effect, we could not prove that a seeding dimer is a catalyst not consumed in the reaction. To circumvent this problem, we synthesized seeding dimers with an isosteric but light-insensitive thioether coupling unit closely mimicking the disulfide linkage. We selected structurally relatively distant dimers (**WF-YF** and **RF-RW**) as parent sequences. Their combination sequence **WF-RW** was also tested. We synthesized the corresponding thioether derivatives **WF-S-YF**, **RF-S-RW**, and **WF-S-RW** (Figure 5a). The mixtures were seeded at a concentration of 10 μM, and the light intensity was set to 5.10 mW cm^–2^. The immediate effects of the seeding were observable (Figure 5b–e) without decomposition of the seed. For dimers with an initial time lag in the unseeded experiment (e.g., **RF-VW**, Figure 5b), the induction time disappeared upon seeding. Seeding with **WF-S-YF** and **RF-S-RW** caused a marked increase in the initial synthesis rates (measured at 40 min) for a number of dimers, including their parent sequences (**WF-YF** and **RF-RW**) (Figure 5d,e). These observations strongly supported the auto- and cross-catalytic effects of the parent dimers. **WF-S-YF** displayed a broad coverage, but the catalytic effect was not uniform: dimers having only hydrophobic side chains were preferred, and a single hydrophilic side chain was tolerated. In contrast, **RF-S-RW** showed a significant initial rate increase only for a small cluster nonoverlapping with that preferred by **WF-S-YF**. **RF-S-RW** accelerated the growth for sequences with two cationic side chains, an identical pattern to the seed. This finding supports the presence of selective templating for the subsets. **WF-S-RW** did not exert a significant catalytic effect on any dimers in the system (Figure 5f). Thus, a continuous passage between the hydrophobic and cationic subsets is not possible by combining a fully hydrophobic and a cationic segment in the seed, indicating considerable templating selectivity.

The rate enhancements in the seeding experiments demonstrated the presence of autocatalysis in the system. We also observed rate enhancements for the nonidentical but similar sequences demonstrating cross-catalysis. These effects are not possible without contact between the dimer products and the monomer precursors. Therefore, templating and proximity must play important roles in the catalytic step. The numeric analysis of the experimental time- and light intensity-dependent data against the dynamic model (see below) independently confirmed that autocatalysis explains the hyperbolic light intensity response.

The sequence dependency is introduced in the templating step, and this effect is dissipatively amplified to the level of the replicator populations. The overall light-induced amplification in the system occurs due to the autocatalytic subsets. Dimers with an effective and large auto-/cross-catalytic cluster can dominate the replicator population at sufficient energy influx. The small size (<1000 Da) and the low complexity of the foldameric segments do not afford a completely specific copy. Nonetheless, the sequence-dependent auto-/cross-catalytic amplification corresponds to a primitive form of chemical replication and selection.

### Far-From-Equilibrium Dissipative Adaptation

Creating and maintaining the various states observed for our system requires UVA irradiation as an external driving force. In the absence of UVA, the concentrations of the high-energy thiyl radical intermediates drop to zero, and the concentrations of the stable disulfide components get frozen at their instantaneous values. Without the continuous energy influx harvested and dissipated by the disulfide–thiyl conversion cycle, the system falls into a nondissipative nonequilibrium state and loses its ability to adapt. On this ground, this system belongs to the class of dissipatively adapting nonequilibrium systems according to the literature definitions.^4,31,32^ A number of published chemical dissipative structures^7,33−35^ relax to equilibrium when the external energy source is depleted or cut off, providing an internal reference to measure the distance from equilibrium. In our system, internal referencing is not feasible, but this feature does not make it nondissipative until the UVA light is on. On the other hand, the thiolate-mediated equilibrium is a natural reference for our system because it is governed by the binding affinities between the foldameric segments.

To estimate the distance of our system from this external reference equilibrium, we compared the photocatalytic dimer concentrations to the values obtained in equilibrium attained through the reversible thiolate-mediated exchange. For the thiolate-mediated equilibrium system, foldamer thiols were used at concentrations identical to the photocatalytic experiments in glutathione redox buffer at pH 8.0. The photocatalytic system was run at pH 7.0 without thiols and redox buffer.

**Figure 6:**
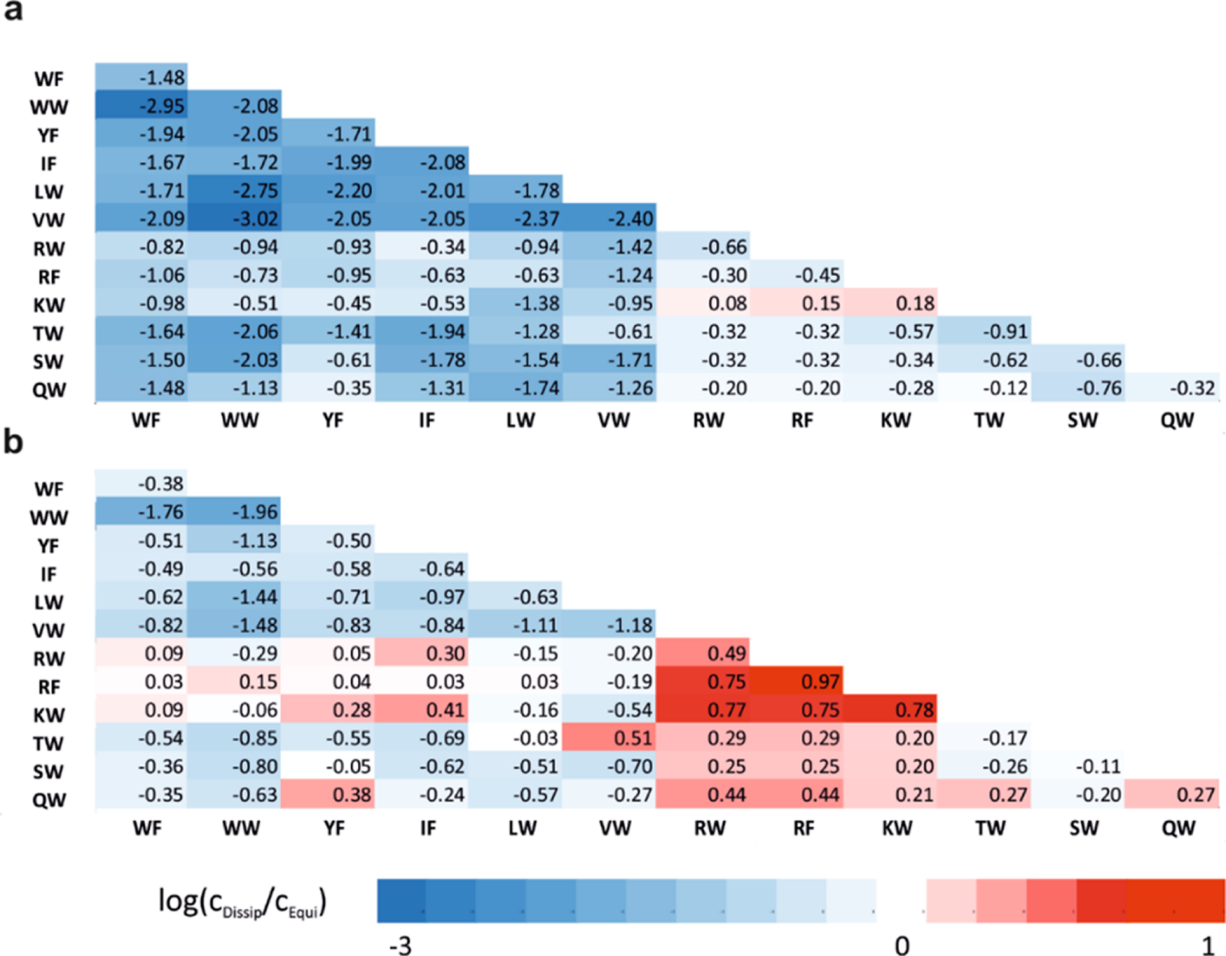
Light intensity- and sequence-dependent adaptation of the dimer concentrations relative to an external reference equilibrium. The logarithm of the concentration ratios (log(*c*_Dissip_/*c*_Equi_)) is displayed on a heat map. *c*_Dissip_ stands for the steady-state concentrations measured in the kinetically asymmetric photochemical exchange at 75% light intensity (a) and 100% light intensity (b). *c*_Equi_ indicates the concentrations obtained in the thiolate-mediated exchange reaction relaxed to equilibrium. The combinations of monomers in the specific dimers are displayed as two-letter codes on the horizontal and vertical axes. The solvent conditions for the photocatalytic reactions were 20 mM HEPES (pH = 7.0), 150 mM NaCl, 2 mM CaCl_2_. The thiolate-mediated equilibrium measurements were performed with foldamer concentrations identical to the photocatalytic experiment, pH 8.0, 20 mM HEPES, redox buffer (500 μM reduced and 125 μM oxidized glutathione), 150 mM NaCl, 2 mM CaCl_2_.

We visualized the significant differences with the logarithm of the concentration ratios (log(*c*_Dissip_/*c*_Equi_)) for each dimer and displayed the values on a heat map (Figure 6). At 75% light intensity, the steady-state dimer concentrations for the hydrophobic sequences are magnitudes below the equilibrium values (Figure 6a) due to the strong bias toward the breakdown mechanism. This finding shows that the system is far from the reference equilibrium already at low energy influx, but the breakdown process is predominant. This situation fundamentally changes at 100% light intensity because light-driven auto-/cross-catalysis successfully competes with the breakdown mechanism (Figure 6b). Hydrophobic sequences are still less favored in the dissipative system, whereas dimers with polar or cationic side chains reach almost a magnitude higher concentrations relative to the reference equilibrium. The light intensity- and sequence-dependent magnitude level deviations from the reference demonstrate the ability of our system to adapt to the energy influx on a broad scale far from equilibrium.

### Dynamic Model for the Photocatalytic Dissipative System

**Figure 7:**
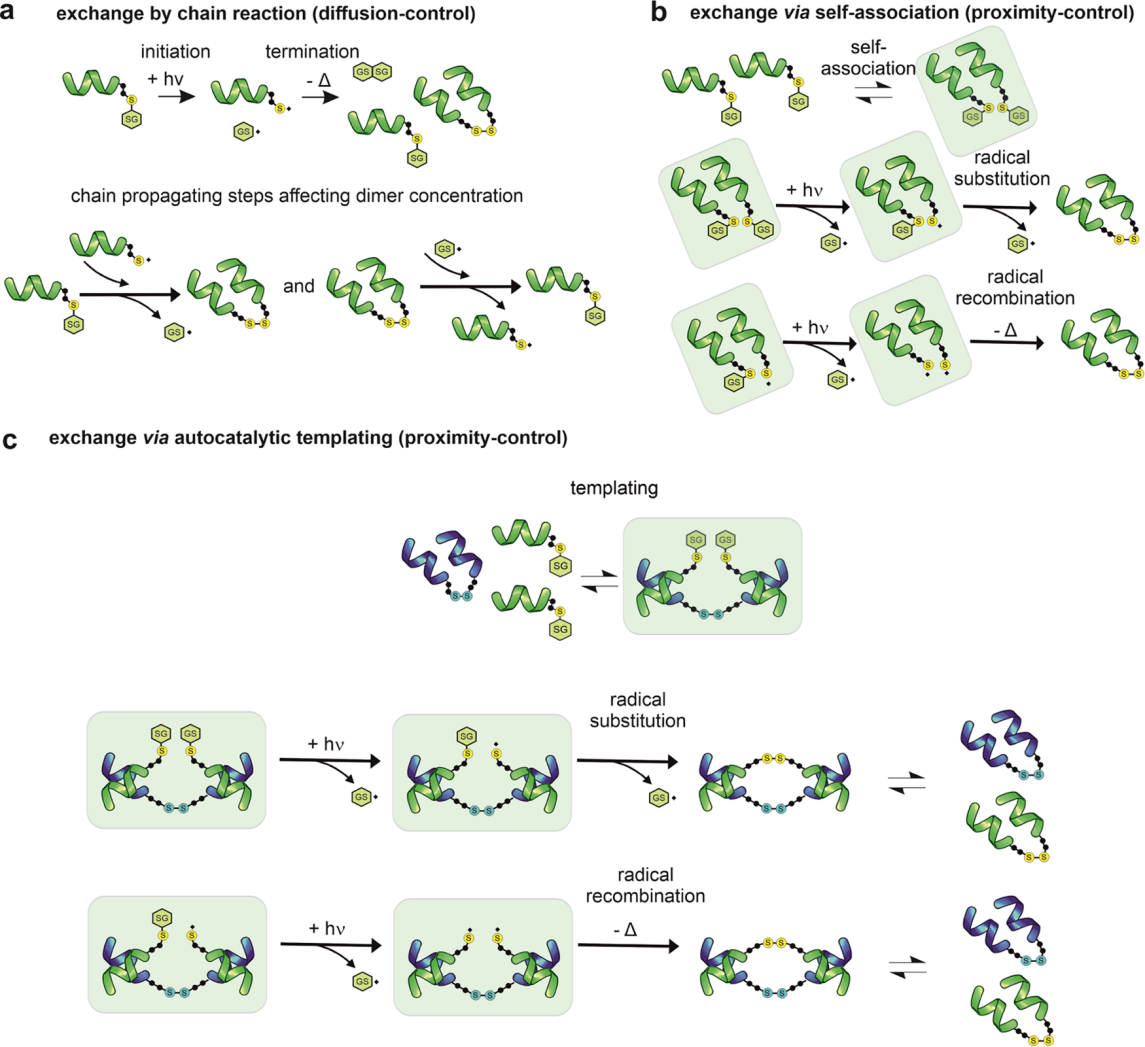
Schematic representation of the dynamic model. (a) Spontaneous synthesis and replicator breakdown through diffusion-controlled radical substitution [3a,b]. (b) Spontaneous synthesis *via* foldamer association preequilibrium and subsequent proximity-controlled radical substitution or concerted metathesis [5]. The intracomplex steps producing dimers can be radical substitution ([7]) and concerted metathesis [9]. (c) Templated autocatalysis under proximity control facilitating the proximity-controlled radical substitution [12] or concerted metathesis [14].

**Scheme 1:**
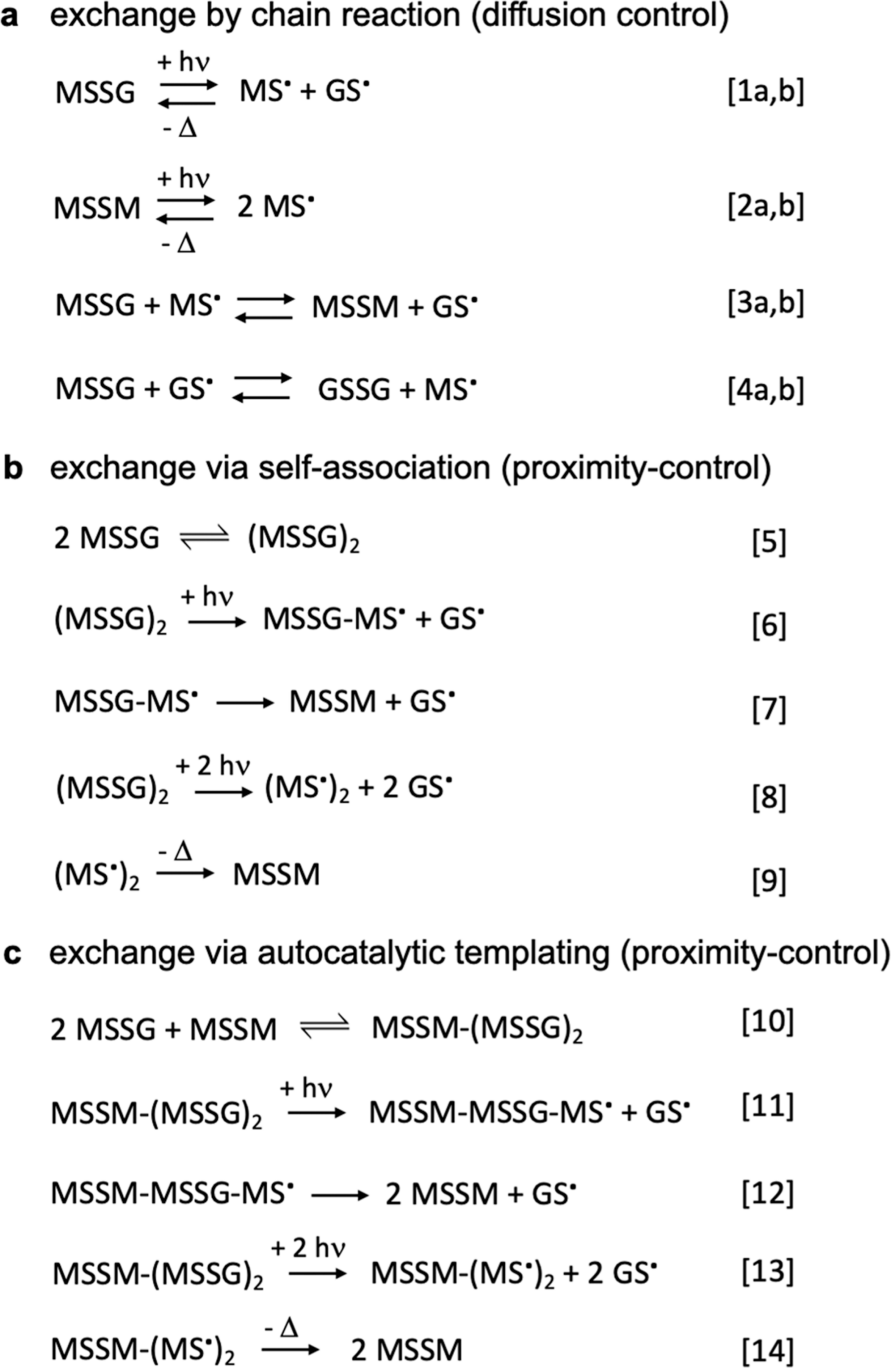
Reaction Mechanisms of the Dynamic Model The separate initiation ([1a] and [2a]), termination ([1b] and [2b]), and chain propagation steps ([3a,b] and [4a,b]) are indicated with the back-and-forth arrows which do not refer to any preequilibrium or microscopic reversibility.

Dissipative systems out of the linear regime can only be handled within the framework of reaction kinetic models.^4^ Therefore, we propose here a dynamic model explaining the experimental findings. The light-induced thiyl radicals participate in a chain reaction as established in the literature^15,18^ (Figure 7a and Scheme 1, [1]–[4]). In the absence of steric inhibition and preferential binding, the predominant exchange route is diffusion-controlled radical substitution (Figure 7a and Scheme 1, [3] and [4]). Based on the preceding literature observation, the rate equations for dimer synthesis [3a] (*v*_s,ch_) and breakdown [3b] (*v*_b_) can be computed (eqs 1 and 2). The detailed calculations are provided in the Supplementary Text.
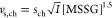
1
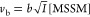
2

The terms *s*_ch_ and *b* represent the corresponding rate constants, and *I* designates light intensity. Symbols [MSSG] and [MSSM] are the monomer and dimer concentrations, respectively. The central experimental finding of this work is that preferential binding (Figure 7b and Scheme 1, [5]) facilitates the proximity-controlled exchange pathways. In this case, the light-induced homolytic cleavage [6] and the subsequent radical substitution [7] occur within the foldameric complexes. We cannot rule out *a priori* a concerted metathesis with coincident absorption of two photons (Figure 7b and Scheme 1, [8] and [9]). In this case, the cross section of the interaction is not decreased by nonlinear two-photon absorption effects because the two disulfides are separately excited. The corresponding rate equations for dimer formation via [7] (*v*_s,p1_) and [9] (*v*_s,p2_) can be derived through a binding preequilibrium and the rate-limiting intracomplex steps (eqs 3 and 4). See the Supplementary Text for the detailed computation.
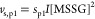
3
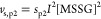
4

The proximity-controlled radical substitution and metathesis rate constants are *s*_p1_ and *s*_p2_, respectively.

The light intensity dependence of the steady-state concentration can be modeled with a hyperbolic curve, which can be obtained for this reaction system by incorporating autocatalysis into the model according to the literature.^36,37^ Indeed, we experimentally confirmed the presence of auto-/cross-catalysis (Figure 7c). This type of templating can exert autocatalysis on dimer formation by both proximity-controlled radical substitution (Figure 7c and Scheme 1, [12]) and concerted disulfide metathesis (Figure 7c and Scheme 1, [14]). The corresponding rate terms (*v*_s,a1_ and *v*_s,a2_) can be calculated (eqs 5 and 6). Details are provided in the Supplementary Text.
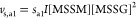
5
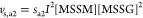
6

The autocatalytic radical substitution and concerted metathesis rate constants are *s*_a1_ and *s*_a2_, respectively.

With eqs 1–6, the steady-state concentration can be expressed (eq 7). However, we observed that the steady-state concentrations steeply converge to zero with decreasing light intensity for all sequences (Figure 3b). This result strongly suggests that the diffusion-controlled radical substitution (Figure 7a and Scheme 1, [3a]) (*v*_s,ch_) has no detectable contribution to the dimer synthesis (*s*_ch_ ≈ 0); that is, the light-independent term is negligible in the numerator of eq 7. Dimer synthesis, therefore, proceeds predominantly via proximity-controlled pathways, whereas breakdown to monomers occurs through diffusion-controlled radical substitution. This observation simplifies eq 7 to eq 8.
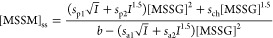
7
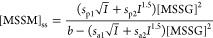
8

**Figure 8:**
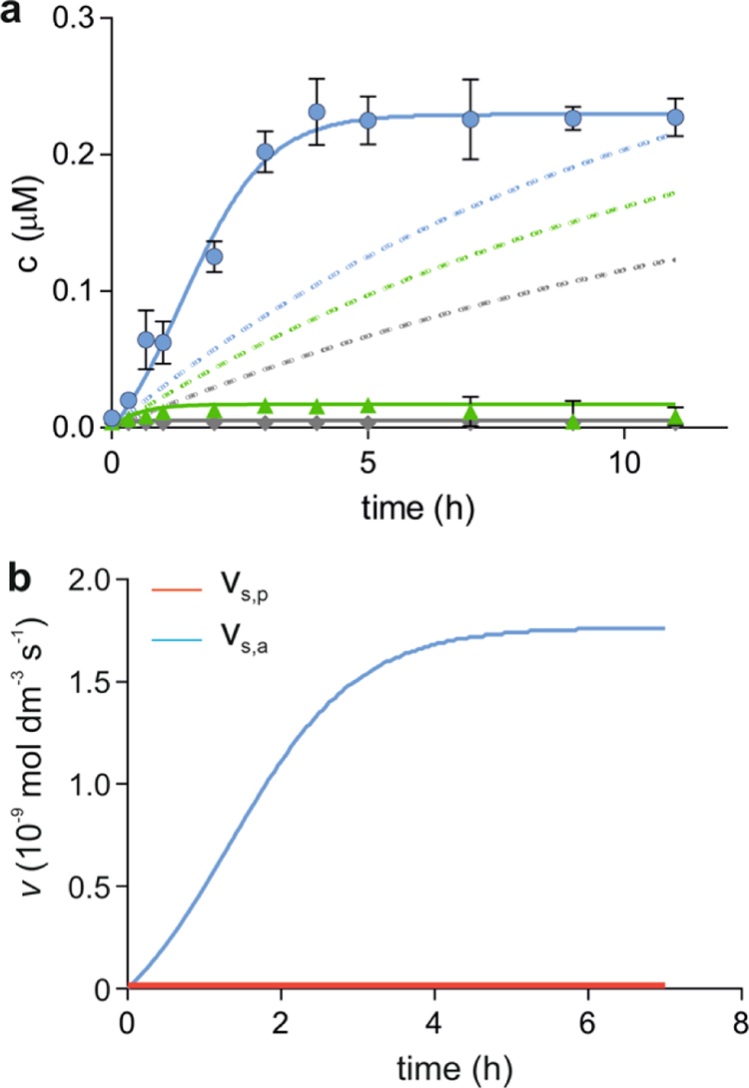
Nonlinear regression of the dynamic model to the time- and light intensity-dependent data array and the effects of autocatalysis on dimer formation. (a) Best-fitting dynamic models with (solid) and without (dashed curves) autocatalysis. Representative experimental time- and light intensity-dependent data array is shown for the dimer **WF-YF** measured at power densities of 2.55 mW cm^–2^ (gray), 3.80 mW cm^–2^ (green), and 5.10 mW cm^–2^ (blue). (b) Time evolution of the reaction rates calculated for the proximity-controlled spontaneous synthesis (*v*_s,p_: red) and the autocatalytic synthesis (*v*_s,a_: blue) in the best-fitting dynamic model for **WF-YF**. The breakdown rate is not indicated in this plot.

Thus, the light intensity dependence also appears in the denominator explaining the hyperbolic function observed in the experiments. The hyperbolic approximation is valid only at low conversions ([MSSG] ≈ [MSSG]_0_), which holds for our experiments. Beyond the steady-state analysis, the dynamic model incorporating eqs 2–6 was used to numerically simulate and fit the time- and light intensity-dependent data arrays with variable rate constants. Nonlinear regressions were carried out simultaneously against all data points measured for the individual dimers. Excellent agreement was found (Figure 8a, solid curves, and Figure S8). The regression of the dynamic model excluding the autocatalytic terms (eqs 5 and 6) failed (Figure 8a, dashed).

Neither the hyperbolic response of the steady-state concentration nor the initial conversion rate changes upon the light intensity increase were captured correctly without the autocatalytic term. This finding supports that the hyperbolic response in the energy influx domain indicates autocatalysis. Together with the seeding results in the time domain, we substantiated autocatalysis with two independent experiments. Time evolution of the reaction rates revealed that the autocatalytic pathway dominates dimer synthesis after 10 min (Figure 8b). The proximity-controlled, spontaneous (non-autocatalytic) mechanism is effective in seeding but would not produce detectable dimer formation within the observed time frame. Inspection of the resulting rate constants indicated that both the proximity-controlled radical substitution and the concerted metathesis mechanisms are present in the system. However, at the level of the individual dimers, the mechanism appeared dependent on the sequence (Table S2). Understanding this substituent-dependent mechanism requires further investigation, which is beyond the scope of the present work.

## Discussion

Disulfide linkage has a rich history, and its nucleophilic exchange mechanism is currently popular in systems chemistry applications.^38,39^ The photocatalytic rearrangement reaction of disulfides has also been known for decades.^15^ However, our work demonstrates the ability of the UV light-induced cleavage-recombination cycle of the disulfide bond to drive kinetically asymmetric processes. The root of asymmetry is the transient binding between peptidic segments that facilitates a fast intracomplex route for the thiyl-mediated exchange reactions under proximity control. In parallel, the diffusion-controlled chain reaction in the background effectively couples disulfides with substituents that rapidly diffuse without preferential binding. The weak interactions can exert proximity control because the high-energy radical intermediates rapidly relax to the dimer before entering the chain reaction. This finding suggests that the weak binding between primitive structures facilitates kinetic asymmetry if the dynamic covalent rearrangement is fast enough.^4,34^ Since the competing mechanisms are affected by the light intensity in different orders, the light not only switched processes on and off, but the energy influx also influenced the extent of kinetic asymmetry. UVA irradiation drives the system to off-equilibrium steady states, ratcheting up the fittest sequences,^2,40^ and the system relaxes into a kinetic trap in the dark. Thus, the system meets the criteria of dissipativity.

The modular nature of the foldameric disulfides allowed templating interactions between the dimers and the monomers. This interaction extends the proximity-controlled mechanism to an autocatalytic process, thereby facilitating a primitive replication. Weak and transient interactions between the foldamer segments govern the binding preequilibrium responsible for the proximity-controlled dimer synthesis. In this regard, the system resembles the two-step crystal nucleation^41,42^ because neither mechanism requires high affinity and specific interactions to accelerate the formation of the final spatial order embodied in the crystal packing or covalent tethering.

In the aqueous environment, the hydrophobic interactions have a major contribution to the helix-helix association. Aromatic and aliphatic side chains have the sufficient hydrophobic surface area to shift the preequilibrium toward the monomer-template complexes. However, the amplification is not determined only by the hydrophobicity level. Sequence **WW-WW** is predominant in the thiolate-mediated equilibrium, while it does not amplify well in the photocatalytic nonequilibrium system. In contrast, **RW-QW** and **KW-TW** show marked amplifications without being very hydrophobic. From these observations, we infer that dimer growth is sensitive to the side chain combinations beyond the overall hydrophobicity. Facilitating rapid turnover of the templated autocatalysis and the proximity of the sulfur-containing functional groups require an optimum helix-helix interaction sensitive to the shape complementarity and physicochemical properties of the foldamers.

Similarly to the chemically fueled replicator systems described in the literature,^9,14^ our system drives the competing replication and the replicator death mechanisms asymmetrically without physical separation of the pathways.^43,44^ Besides exploring the mechanistic details of the dissipative replication and selection phenomena, there can be further technical advantages for the light-driven replicator system: (i) it does not generate waste products, which is potentially compatible with all classes of chemical structures that survive UVA, (ii) controlling the level of the input energy is straightforward, (iii) the kinetic asymmetry is controlled by molecular recognition events, which opens up new ways to gain rational control over the dissipative replication and selection.

In this system, the tendency toward assembly into structures of increased complexity is amplified by replication. In contrast, rapid diffusion—a property associated with low complexity—promotes decomposition. Thus, the dissipative adaptation^4,45^ in the present system helps elucidate the chemical mechanisms of spontaneous emergence of complexity by selection. Pioneering experiments with dissipative self-assembling systems established the principles that govern chemical energy^46−48^ or light-driven^49,50^ trajectories to off-equilibrium states that generate spatial proximity-based order.^3,35,51−53^ The dissipative self-organization of material can be a source of structural complexity^7,54,55^ linked to the emergence of life. Peptide-disulfides in the photochemical exchange reaction can be a valuable tool for studying the dissipative self-organization of simple building blocks.

## Conclusions

We successfully coupled the light-harvesting thiyl radical chemistry with the molecular recognition processes occurring in a foldameric system. This system unexpectedly displayed a primitive replication mechanism, a kinetically asymmetric replicator death pathway, and dissipative adaptation to the external energy source and seeding. Although these features are essential for mimicking chemical evolution, prebiotic chemistry is beyond the scope of this study. However, the chemical availability of primitive Cys-containing peptides is supported by prebiotic Cys-catalyzed amino acid and peptide synthesis.^56,57^ These findings make thiyl radical-mediated dissipative replication of short, folding peptidic sequences an intriguing mechanism,^58^ potentially illuminating energetic aspects of the transition from prebiotic chemical systems to biotic evolution.

## Supporting Information Available

Synthesis, purification, and characterization of peptides; further experimental details (representative total ion chromatograms, HPLC-MS characterization, and evaluation data); calculations used for evaluation; mathematical description of the dissipative model; time-dependent experimental data with fitted curves; fitted rate constants; 2D NMR spectra proving the helicity of the compounds; seeding experiment with **WF-YF**; and supporting tables containing seeding data (PDF)

## Abbreviations

ACHC: 1S,2S-2-aminocyclohexanecarboxylic acid
SG: glutathione
