# Supplementary material for: Light-Fueled Primitive Replication and Selection in Biomimetic Chemical Systems
Source: J Am Chem Soc. 2023 Jun 7;145(24):13371–83. doi: 10.1021/jacs.3c03597 (PMC10288511; doi:10.1021/jacs.3c03597)
Supplement: Supplementary file 1 — ja3c03597_si_001.pdf [file ja3c03597_si_001.pdf]

Supporting Information

Light-fueled primitive replication and selection in biomimetic chemical systems

Éva Bartus†,§, Attila Tököli†, Beáta Mag†, Áron Bajcsi†, Gábor Kecskeméti†, Edit Wéber†,§, Zoltán Kele†, Gabriel Fenteany†,‡,§, Tamás A. Martinek†,§*
†Department of Medical Chemistry, University of Szeged, Dóm tér 8, H-6720 Szeged, Hungary
§ELKH-SZTE Biomimetic Systems Research Group, University of Szeged, Dóm tér 8, H-6720 Szeged, Hungary
‡Institute of Genetics, Biological Research Centre Temesvári krt. 62, H-6726 Szeged, Hungary

*Email: martinek.tamas@med.u-szeged.hu

Table of Contents

Table of Contents **2**
Experimental Procedures **3**
- Synthesis and purification of the foldameric sequences 3
- Synthesis and purification of the glutathione protected monomers 3
- LC-MS analysis of the purified glutathione protected monomers 3
- Synthesis and purification of thioether dimers RF-S-RW, WF-S-YF and WF-S-RW 3
- Preliminary experiments for testing disulfide rearrangement in a foldamer library 3
- UVA-induced disulfide exchange reaction 4
- LC-MS measurements and MS data analysis 4
- Generating statistical dimer distribution and calibration of the MS-AUC-to-concentration conversion 4
- Generating equilibrium dimer distribution through thiolate-mediated exchange 5
- Calculation of the light intensity-dependent amplification factor 5
- Calculation of the concentration amplifications for the dimers upon seeding 5

Supplementary Text **6**
- Equation (1): Rate of spontaneous dimer synthesis through diffusion-controlled radical substitution 6
- Equation (2): Rate of dimer breakdown through diffusion-controlled radical substitution 6
- Equations (3) and (4): Rates of dimer synthesis through proximity-controlled mechanisms 7
- Equations (5) and (6): Rates of autocatalytic dimer synthesis 8
- Dynamic model for the foldamer-based photochemical disulfide exchange system 8
- Fitting the dynamic model to the experimental time- and energy-dependent data arrays 8

Supplementary Figures **9**
Supplementary Tables **19**
Peptide characterisation data **24**
References **39**

Experimental Procedures

Synthesis and purification of the foldameric sequences

Foldameric sequences having L-Gly-L-Gly-L-Cys C-terminal segments were synthesised manually by standard solid-phase peptide synthesis with Fmoc/tBu chemistry. Rink Amide AM resin was used as solid support (capacity: 0.71 mmol/g) and HATU (1-[bis(dimethylamino)methylene]-1*H*-1,2,3-triazolo[4,5-b]pyridinium 3-oxid hexafluorophosphate) as coupling reagent in the presence of DIEA (*N,N*-diisopropylethylamine). Amino acids and coupling reagents were used in excess of 3 equivalents and shaking was applied at room temperature for 3 h. Deprotection was carried out in a DMF (*N,N*-dimethylformamide) solution containing 2% DBU (1,8-diazabicycloundec-7-ene) and 2% piperidine. Cleavage was performed with TFA/H_2O/DTT (DL-dithiothreitol)/TIS (triisopropylsilane) (90:5:2.5:2.5), which was followed by precipitation in ice-cold diethyl ether. The resin was washed with acetic acid and water, filtered, then lyophilised. Peptides were purified by RP-HPLC on a C18 column (Phenomenex Luna C18, 250 x 10.00 mm, particle size: 10 µm, pore size: 100 Å). The HPLC eluents were 0.1% TFA in water (Eluent A), and 0.1% TFA/ 80% ACN (acetonitrile) in water (Eluent B). Different gradient elution was used according to the hydrophobicity of the peptides. Purity was confirmed by analytical RP-HPLC and ESI-MS measurements.

Synthesis and purification of the glutathione protected monomers

Glutathione-protected monomers were synthesised by oxidative coupling of thiols in the solution phase. Each purified foldamer was dissolved in 20% DMSO (dimethyl sulfoxide) in water separately to 1 mM concentration in the presence of 20× excess of GSH (reduced glutathione) and stirred overnight at room temperature exposed to atmospheric oxygen. The completeness of the oxidation reaction was monitored by HPLC-MS, and the reaction mixture was injected directly onto a semi-preparative HPLC column (Phenomenex Luna C18, 250 × 10.00 mm; particle size: 10 µm; pore size: 100 Å) and purified. Under this reaction condition the amount of the homodimeric foldamer was negligible and could be completely separated from the foldamer-glutathione adduct.

LC-MS analysis of the purified glutathione protected monomers

UHPLC-MS/MS measurements were used to characterize the pure peptides by using an ACQUITY I-Class UPLC™ liquid chromatography system (Waters, Manchester, UK) coupled with a Q Exactive™ Plus Hybrid Quadrupole-Orbitrap Mass Spectrometer (Thermo Fisher Scientific, San Jose, CA, USA). Chromatographic separation was carried out at 25 °C using 0.1% formic acid in water as solvent A and ACN containing 0.1% formic acid as solvent B. The following multistep gradient was used: 5-50% over 20 minutes then 50-80% over 5 minutes and finally 80% solvent B for additional 5 minutes at 0.7 mL min^{-1} flow rate. Samples where incubated at 5°C until the measurement and 15 µL of the sample was injected into the UHPLC–MS/MS system. The MS instrument was operated in the positive-ion mode using the equipped HESI-II source with the following parameters: capillary temperature: 256°C; spray voltage: 3.5 kV; aux gas heater temperature: 412°C; sheath gas flow: 47.5 mL min^{-1}; aux gas flow: 11 mL min^{-1}; and S-lens RF level, 50.0 (source auto-defaults). Full scan was conducted with a mass range of 150–2000 *m/z* with resolution of 70,000. The ACG (automatic gain control) setting was defined as 3×10^6 charges, and the maximum injection time was set to 100 ms. Data dependent MS/MS was acquired in a mass range of 200-2000 *m/z* with resolution of 17,500. AGC setting was defined as 5×10^5 charges, and the maximum injection time was set to 150 ms.

Synthesis and purification of thioether dimers RF-S-RW, WF-S-YF and WF-S-RW

Chloroacetylated **RF** and **WF** was synthesized on a solid support with C-terminal 4-methyltrityl (Mtt)- protected lysine. The Mtt protecting group was eliminated with a treatment of AcOH/TFE/DCM (2:1:7) for 1 h. Chloroacetic acid was coupled to the ε-amino group of lysine in excess of 5 equivalents with DCC/HOAt activation. The crude peptides (**RF**-Lys(ClAc) and **WF**-Lys(ClAc)) were cleaved from the resin with a mixture of TFA/H_2O/TIS (92:5:3) and followed by precipitation in ice-cold diethyl ether. The resin was washed with acetonitrile and water, then filtered and lyophilized. Crude peptides were purified by RP-HPLC on a C18 column (Phenomenex Luna, 250 x 10.00 mm). The purified chloroacetylated peptides were dissolved in 0.1 M Tris buffer (pH = 8.2) with acetonitrile as a co-solvent. Previously purified **RW**, **YF** or **RW** peptides containing Gly-Gly-Cys linker at the C-terminus were dissolved in the same buffer in 1.5 molar excess and added to the reaction mixture under continuous stirring. After overnight incubation at room temperature, the mixture was purified on a C18 HPLC column.

Preliminary experiments for testing disulfide rearrangement in a foldamer library

Two setups for the starting systems were used for testing. First, four different glutathione protected monomers, **WF-G, RW-G, LW-G** and **TW-G** were dissolved in 20 mM HEPES buffer with 150 mM NaCl and 2 mM $CaCl_2$ (pH=7.0) each one in 10 µM concentration. Second, the 10-membered dimeric library was prepared by slow oxidation of the thiol forms of the foldamers, **WF-SH, RW-SH, LW-SH** and **TW-SH**, which formed the statistical concentration distribution. The components were transferred into the previously defined buffer so that each heterodimer had a concentration of 10 µM. An excess of oxidized glutathione (500 µM) was added to this latter mixture.

The reaction mixtures were transferred separately into quartz cuvettes having PTFE stopper and kept under argon atmosphere during the experiment. Solutions were stirred at 150 RPM and kept at constant temperature of 303±1 K *via* air cooling system. Continuous irradiation of the samples was carried out by UVL-28 EL Series UV Lamp at 365 nm (Analytic Jena US, Upland, CA). Product distribution was monitored with HPLC-MS. To directly test the effect of UV in the disulfide rearrangement, a control experiment was performed from the mixtures and kept under the same condition but without UV-irradiation.

UVA-induced disulfide exchange reaction

Twelve different monomers were dissolved in 20 mM HEPES, 150 mM NaCl, 2 mM $CaCl_2$ (pH = 7.0), with each monomer at a final concentration of 10 µM. 2.0 mL reaction mixture was transferred into quartz cuvettes having a PTFE stopper and kept under an argon atmosphere during the experiment. Solutions were stirred at 150 RPM and kept at a constant temperature of 303 ± 1 K with an air-cooling system (modified Jasco Jetstream 2 Plus Column Thermostat). The temperature was monitored with a laser gun thermometer. Continuous illumination of the samples was carried out with a UVL-28 EL Series UV lamp (Analytic Jena US, Upland, CA). The distance dependence of the power density and the emission spectrum of the lamp were determined prior to the experiments (Figure S10), and the irradiation intensity was controlled by the distance between the lamp and the sample (Figure S11). 200 µL samples were taken from the reaction mixture at each time point. Placing the sample in the dark froze the reaction; no relaxation to equilibrium occurred. To eliminate any slow non-photocatalytic disulfide exchange reaction in the mixture until the analysis, we added 100 µL of 10% TFA in water. In the case of seeded experiments, 100 µL ACN was added to the sample in order to prevent aggregation. The product distribution of the samples was analysed using HPLC/ESI-MS measurements.

LC-MS measurements and MS data analysis

LC-MS analysis was performed with a Dionex UltiMate 3000 HPLC system interfaced with an LTQ ion trap mass spectrometer (Thermo Electron Corp., San Jose, CA, USA). Samples were injected onto an AerisTM Widepore XB-C18 (250 x 4.6 mm, particle size: 3.6 µm, pore size 100Å) analytical HPLC column using gradient elution 5-80% solution B for 25 min at 0.7 mL min-1 flow rate. Eluent composition was 0.1% formic acid in distilled water (Solution A) and 0.1% formic acid in acetonitrile (Solution B). The MS instrument was operated in the positive-ion mode using the equipped HESI-II source with the following parameters: capillary temperature: 350°C; spray voltage: 3.0 kV; source heater temperature: 250°C; sheath gas flow: 30 mL min^{-1}; aux gas flow: 10 mL min^{-1}. Mass spectra were acquired in full-scan mode from 200 to 2000 m/z. Thermo Xcalibur 2.2 software was used for peak identification and integration. 96% of the foldameric building blocks could be resolved independently by HPLC-MS/MS measurements based on molecular weight, MS fragmentation pattern and retention time depending on the relative hydrophobicity of the side chains. Those components which could not be resolved independently were integrated and averaged. A representative raw file for the library was utilised to create a processing method, where each sample component was associated with a chromatographic peak based on the previously identified mass (m/z) and retention time (Table 1, Table S3-4). Using the ICIS peak detection algorithm, the general detection and integration criteria were: smoothing points: 5; baseline window: 60; area noise factor: 5; peak noise factor: 10. All raw data files were reprocessed with these processing setups together and analysed. Errors in peak identification during the automatic processing were corrected manually.

Generating statistical dimer distribution and calibration of the MS-AUC-to-concentration conversion

Statistical product distribution for the dimers were generated in a chaotropic solvent mixture of 20% DMSO:80% water. Glutathione-protected monomers were dissolved (each one at 10 µM final concentration) and the library was completely reduced with 2 molar equivalents of TCEP (tris(2-carboxyethyl)phosphine). Subsequently, the sample was continuously stirred for 48 h exposed to atmospheric oxygen.

To confirm that oxidation in the chaotropic solvent (DMSO:water) results in statistical product distribution, a subset of monomers was analysed for which self-association tendency is significantly different (**WF-G**, **LW-G**, **RW-G** and **TW-G**). The oxidised mixture was analysed using HPLC-UV with the detection wavelength of 210 nm, where the integrated intensity is directly proportional to the peptide concentration. For this system, the oxidation products could be quantitatively analysed in HPLC-UV without problem. As expected, the reaction yielded four homodimers and six heterodimers with statistical concentration distribution (Figure S12).

Next, we performed the same experiment starting from the 12-membered monomer library. Completeness of the oxidation after the TCEP treatment was confirmed, and the final product distribution was quantitatively analysed with HPLC-MS. Due to the large number of components, UV detection could not separate the products. Based on the model experiment, the statistical concentration of each dimer was predicted and then calculated. After the quantitative evaluation of the HPLC-MS chromatograms, the MS-AUC/concentration ratios were calculated for each component (Table S5).

Generating equilibrium dimer distribution through thiolate-mediated exchange

The twelve different thiol-functionalized foldameric building blocks (**IF-G, KW-G, LW-G, QW-G, RW-G, RF-G, SW-G, TW-G, VW-G, WF-G, WW-G** and **YF-G**) were dissolved at a concentration of 10 µM in a redox buffer (pH 8.0, 20 mM HEPES, 150 mM NaCl, 2 mM $CaCl_2$, 3 mM NaN_3, 500 µM GSH and 125 µM GSSG). Reaction mixture was shaken at 250 rpm, 37°C for three days in a properly closed vial. Samples (100 µL) were taken from the mixture every 24 h, quenched with 10% TFA in water (50 µL) and analysed with HPLC-MS[1]. Product distribution was obtained via quantitative evaluation of the HPLC-MS chromatograms and conversion of AUC-to-concentration was carried out as described above (Figure S1, Table S5)

Calculation of the light intensity-dependent amplification factor

Light intensity-dependent amplification factors (AF) were calculated for each dimer to determine the sensitivity to the energy influx. The following formula (eq. (S12)) was used:

$$AF_{i(C),int} = \frac{AUC_{i(C),100\%}}{AUC_{i(C),50\%}} \tag{S12}$$

where $AUC_{i(C),100\%}$ is the area under the curve (AUC) of compound *i* measured at 100% light intensity (5.10 mW cm^{-2}) and $AUC_{i(C),50\%}$ is the AUC of the same compound measured at 50% light intensity (2.55 mW cm^{-2}). Errors of AF_i (σ_{AF}) were calculated from three parallel measurements with the following general formula (neglecting the correlation between the variables):

$$\sigma_{AFi} = AF_i\sqrt{\left(\frac{\sigma_{AUC1}}{AUC_1}\right)^2 + \left(\frac{\sigma_{AUC2}}{AUC_2}\right)^2} \tag{S13}$$

where $AUC_1 \pm \sigma_{AUC1}$ and $AUC_2 \pm \sigma_{AUC2}$ are the measured variables with uncertainties when AF_i is calculated by AUC_1/AUC_2.

Calculation of the concentration amplifications for the dimers upon seeding

Concentration amplifications (CA) were calculated for each dimer to evaluate the catalytic effect of seeding dimer (**WF-S-YF**). The following formula (S14) was used for the calculation:

$$CA_{i,seeding} = \frac{AUC_{i,seeded}}{AUC_{i,control}} \tag{S14}$$

Where $AUC_{i,\ seeded}$ is the area under the curve (AUC) of compound i measured in the presence of the seeding compound and $AUC_{i,\ control}$ is the AUC of the same compound measured without the seeding compound.

Supplementary Text

Equation (1): Rate of spontaneous dimer synthesis through diffusion-controlled radical substitution

In this mechanism, dimers (MSSM) are produced by the reaction of freely diffusing monomer radicals (MS·) with glutathione-protected monomers (MSSG) as shown in reaction [3a] (Figure 1b). Here, "MS" corresponds to a single foldameric sequence.

$$\mathrm{MSSG + MS^{\bullet} \longrightarrow MSSM + GS^{\bullet}} \qquad [3a]$$

The reaction rate for [3a] ($v_{s,ch}$) satisfies eq. (S1),

$$v_{s,ch} = k_{ch}[\mathrm{MS}^{\cdot}][\mathrm{MSSG}] \qquad \text{(S1)}$$

where k_{ch} is the rate constant for the radical substitution step. In radical chain reactions, [MS·] can be approximated with a quasi-steady state approach. At low conversions, MS· is produced preferentially by homolytic cleavage of the starting material MSSG. Therefore, the rate of formation is proportional to the light intensity ($k_I I$) and [MSSG]. The chain-termination steps consuming MS· involve collisions with MS· and GS·. Again, the large excess of MSSG at low conversions affords the approximation that reaction [1a] (Figure 1b) is dominant. Thus, there is a 12× molar excess for [GS·], that is, [GS·] ≈ 12[MS·]. Consequently, the chain-termination rate can be expressed as a second order function in [MS·] (eq. (S2)).

$$\frac{d[MS^{\cdot}]}{dt} = 0 = k_I I[\mathrm{MSSG}] - k_t[\mathrm{MS}^{\cdot}]^2 - k_t 12[\mathrm{MS}^{\cdot}]^2 \qquad \text{(S2)}$$

$$[\mathrm{MS}^{\cdot}] = \sqrt{\frac{k_I I}{13k_t}[\mathrm{MSSG}]} \qquad \text{(S3)}$$

Here, k_I is the rate constant of the light-induced homolytic cleavage, and k_t is the rate constant for the chain-termination reaction. Substituting eq. (S3) into eq. (S1) and collecting the concentration- and light intensity-independent terms into the constant s_{ch}, the rate is given by eq. (1).

$$v_{s,ch} = s_{ch}\sqrt{I}[\mathrm{MSSG}]^{1.5} \qquad (1)$$

Equation (2): Rate of dimer breakdown through diffusion-controlled radical substitution

The decomposition of MSSM proceeds in this mechanism through the reaction between the freely diffusing glutathione radical (GS·) and MSSM (reaction [3b]).

$$\mathrm{MSSM + GS^{\bullet} \longrightarrow MSSG + MS^{\bullet}} \qquad [3b]$$

The rate equation can be expressed as follows (eq. (S4)).

$$v_b = k_b[\mathrm{GS}^{\cdot}][\mathrm{MSSM}] \qquad \text{(S4)}$$

For [GS·], we apply the quasi-steady state approximation again. The source of [GS·] is the light-induced homolytic cleavage of MSSG and GSSG. The chain-termination steps consuming GS· involve collisions with GS· and MS·. Due to the 12× excess of GS· over MS· in the system studied, the latter reaction can be neglected. Thus, we obtain eq. (S5).

$$\frac{d[\mathrm{GS}^{\cdot}]}{dt} = 0 = k_I I([\mathrm{MSSG}] + 2[\mathrm{GSSG}]) - k_t[\mathrm{GS}^{\cdot}]^2 \qquad \text{(S5)}$$

Due to the mass balance for the ‘GS’ moiety, the term [MSSG] + 2[GSSG] is constant and equals to the initial concentration of MSSG ($[\mathrm{MSSG}]_0$), which yields eq. (S6).

$$[\mathrm{GS}^{\cdot}] = \sqrt{\frac{k_I I}{k_t}[\mathrm{MSSG}]_0} \tag{S6}$$

Thus, $[\mathrm{GS}^{\cdot}]$ is proportional to the square root of the light intensity, and eq. (S6) can be substituted into (S4). Collecting the constant term into the overall rate constant of *b*, we obtain eq. (2).

$$v_b = b\sqrt{I}[\mathrm{MSSM}] \tag{2}$$

Equations (3) and (4): Rates of dimer synthesis through proximity-controlled mechanisms

This mechanism begins with the association preequilibrium producing the complex $(\mathrm{MSSG})_2$.

$$2\ \mathrm{MSSG} \rightleftharpoons (\mathrm{MSSG})_2 \qquad [5]$$

$$[(\mathrm{MSSG})_2] = \frac{1}{K_{D,p}}[\mathrm{MSSG}]^2 \tag{S7}$$

$K_{D,p}$ stands for the dissociation constant. The rate-determining step is the photochemical cleavage of a monomer within the complex (reaction [6]). If the geometry of the complex is advantageous, the resulting high-energy radical can rapidly relax through the intracomplex radical substitution [7].

$$(\mathrm{MSSG})_2 \xrightarrow{+\,h\nu} \mathrm{MSSG\text{-}MS}^{\bullet} + \mathrm{GS}^{\bullet} \qquad [6]$$

$$\mathrm{MSSG\text{-}MS}^{\bullet} \longrightarrow \mathrm{MSSM} + \mathrm{GS}^{\bullet} \qquad [7]$$

The rate of formation for $\mathrm{MSSG\text{-}MS}^{\cdot}$ is proportional to the light intensity ($k_I I$) and $[(\mathrm{MSSG})_2]$. Using eq. (S7), the rate equation can be obtained (eq. (S8)).

$$v_{s,p1} = \frac{k_I I}{K_{D,p}}[\mathrm{MSSG}]^2 \tag{S8}$$

Substituting the constant term with the overall rate constant s_{p1} yields eq. (3).

$$v_{s,p1} = s_{p1} I [\mathrm{MSSG}]^2 \tag{3}$$

Literature results showed that recombination of two thiyl radicals is a possible photochemical exchange mechanism, when radical substitution is sterically hindered[2]. Therefore, the intracomplex version of this mechanism cannot be ruled out *a priori*. If the absorption of the two photons is consecutive, the reaction rate remains first order in light-intensity; that is, the functional form of the rate equation is the same as eq. (3). If coincident absorption of two photons produces the diradical intermediate $(\mathrm{MS}^{\cdot})_2$ and MSSM is formed in a concerted manner (steps [8] and [9]), the probability of the interaction with two photons is proportional to the square of the light intensity.

$$(\mathrm{MSSG})_2 \xrightarrow{+\,2\,h\nu} (\mathrm{MS}^{\bullet})_2 + 2\ \mathrm{GS}^{\bullet} \qquad [8]$$

$$(\mathrm{MS}^{\bullet})_2 \xrightarrow{-\,\Delta} \mathrm{MSSM} \qquad [9]$$

The cross-section of the interaction is not decreased by the non-linear "two-photon absorption" effect, because disulfides in the complex are not coupled quantum mechanically. Moreover, the recombination of the diradical [9] does not involve any activation energy, leading to a very fast reaction. On this ground, we incorporated this mechanism into the model with the rate equation (4).

$$v_{s,p2} = s_{p2} I^2 [\mathrm{MSSG}]^2 \tag{4}$$

Equations (5) and (6): Rates of autocatalytic dimer synthesis

The autocatalytic mechanism starts with a binding preequilibrium between the dimers and the monomers ([10]). The dissociation constant ($K_{D,a}$) determines the concentration of the complex available for the further, rate determining step.

$$2\ \text{MSSG} + \text{MSSM} \rightleftharpoons \text{MSSM-(MSSG)}_2 \qquad [10]$$

$$[\text{MSSM-(MSSG)}_2] = \frac{1}{K_{D,a}}[\text{MSSM}][\text{MSSG}]^2 \qquad \text{(S9)}$$

The calculation of the rates for the autocatalytic routes is closely analogous to the non-autocatalytic synthesis pathways described above. For the intracomplex radical-substitution pathway, the rate-determining step is the absorption of a photon, which leads to a linear light-intensity dependence of the reaction rate. Using (S9), eq. (6) is obtained.

$$v_{s,a1} = s_{a1} I [\text{MSSM}][\text{MSSG}]^2 \qquad (5)$$

Coincident absorption and the concerted conversion to MSSM is also possible for the autocatalytic complex, which yields a rate equation quadratic in light intensity (eq (7)).

$$v_{s,a2} = s_{a2} I^2 [\text{MSSM}][\text{MSSG}]^2 \qquad (6)$$

Dynamic model for the foldamer-based photochemical disulfide exchange system

The mathematical framework of chemical evolution[3] was invoked to analyse the experimentally determined energy-dependent dynamics of the dimer synthesis. The differential equation describing the time- and light intensity-dependent concentration of the dimers ([MSSM]) contained the following rate terms (eq. (S10)): non-autocatalytic (spontaneous) synthesis ($v_{s,p1}$ and $v_{s,p2}$), autocatalytic synthesis ($v_{s,a1}$ and $v_{s,a2}$) and break down (v_b).

$$\frac{d[\text{MSSM}]}{dt} = v_{s,p1} + v_{s,p2} + v_{s,a1} + v_{s,a2} - v_b \qquad \text{(S10)}$$

Fitting the dynamic model to the experimental time- and energy-dependent data arrays

Differential equation (S10) was numerically integrated using the Runge-Kutta (RK4) method to simulate the light intensity-dependent time evolution of a dimer. The synthesis rates are dependent on the actual monomer concentration, which was expressed with [MSSM] using the mass balance (S11).

$$[\text{MSSG}] = [\text{MSSG}]_0 - 2[\text{MSSM}] \qquad \text{(S11)}$$

The numeric integrations were carried out with the parameters of $[\text{MSSM}]_0 = 0$, $[\text{MSSG}]_0 = 2\times10^{-5}$ M and $\Delta t = 4.17\times10^{-2}$ h. The light intensities were set to the calibrated values. The rate constants s_{p1}, s_{p2}, s_{a1}, s_{a2} and b were determined by fitting (non-linear regression) the simulated curves against the time- and light intensity-dependent experimental data array.

Supplementary Figures

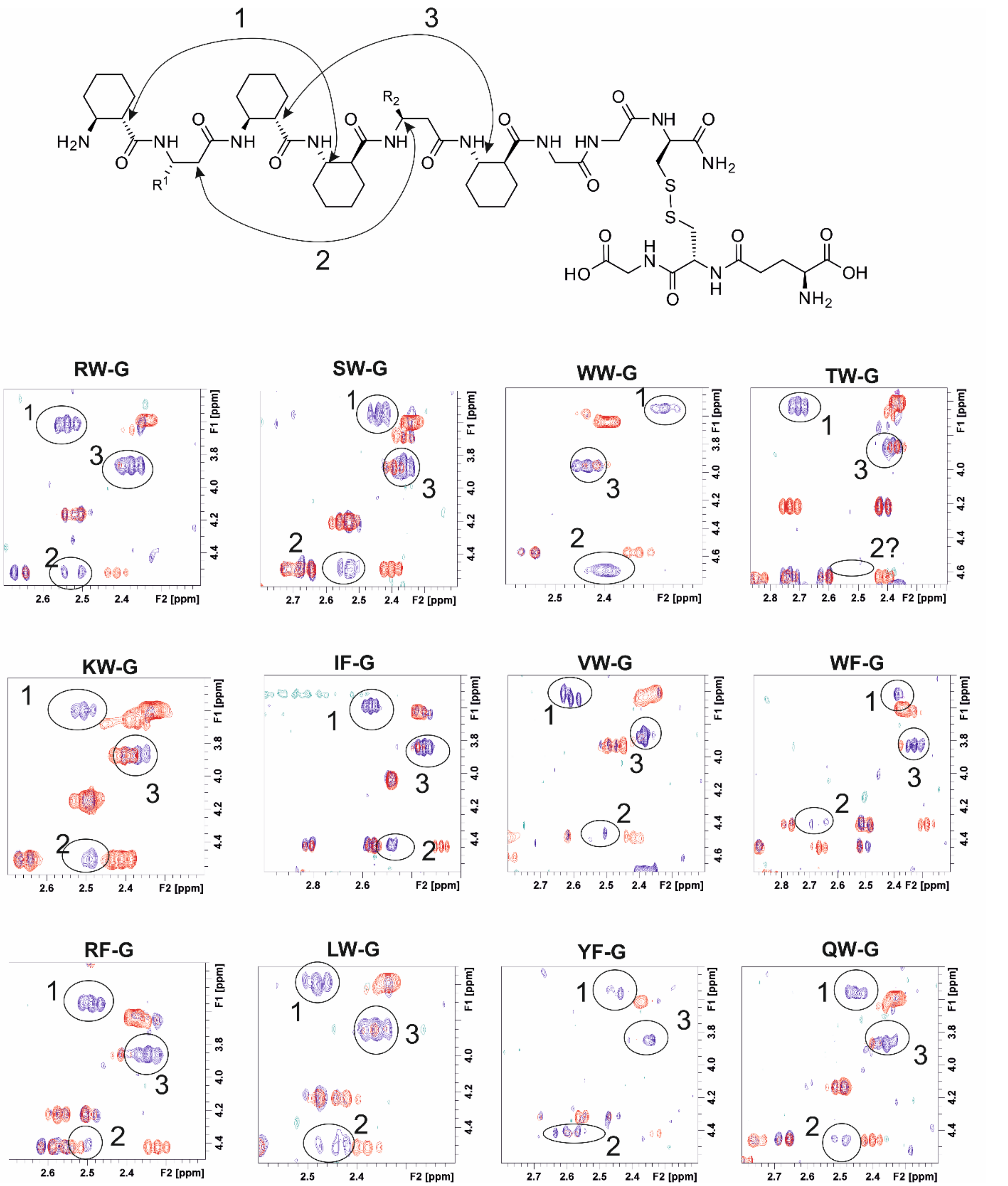

Figure S1. Helical structure of the monomers proven by NMR spectroscopy. Informative Hα-Hβ regions of the overlaid 2D ROESY and TOCSY NMR spectra (blue and red, respectively). Circled cross peaks in the spectra labelled with 1, 2 and 3 numbers can be assigned to long-range NOE interactions, which are represented with arrows at the general structure of the monomers (top), and are characteristic of the H14 helix. NMR experiments were acquired at 298 K with a Bruker Avance III 600 MHz spectrometer equipped with a 5 mm CP-TCI triple-resonance cryoprobe. 2D TOCSY measurements were performed with homonuclear Hartman–Hahn transfer with a mixing time of 80 ms (DIPSI2 sequence). 2D ROESY spectra were recorded with a mixing time of 400 ms. Glutathione protected monomer peptides were dissolved in 20 mM pH 7.0 d_{18}-HEPES (90% H_2O, 10% D_2O) or in H_2O containing 10% D_2O at a concentration of 100 to 200 μM. Spectra were processed with Topspin 3.6.1.

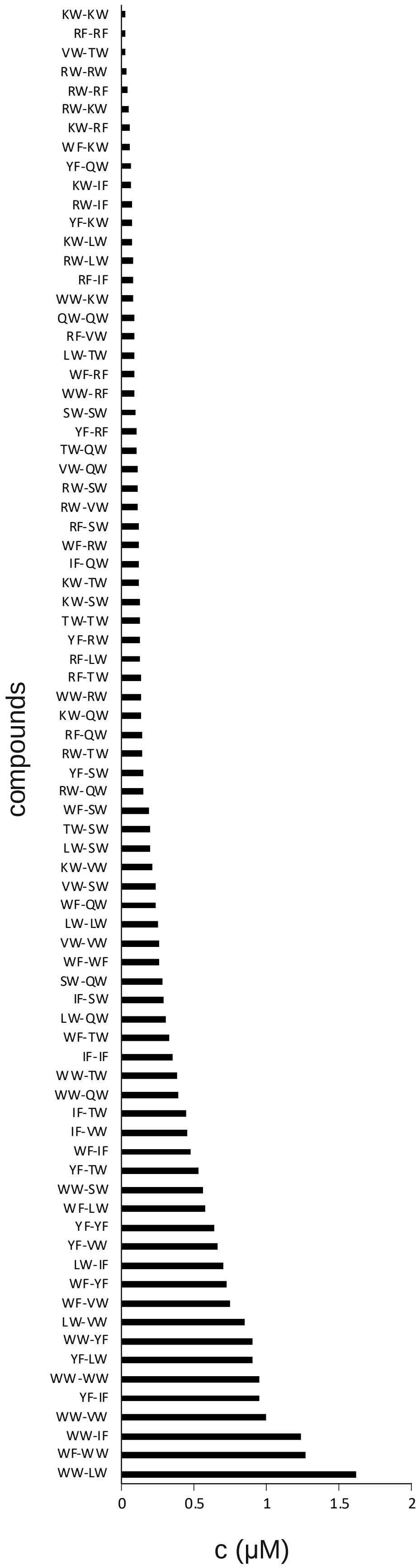

Figure S2. The equilibrium product distribution of foldameric dimers. Equilibrium product distribution was measured in an aqueous solution. The twelve different thiol-functionalized foldameric building blocks (IF-G, KW-G, LW-G, QW-G, RW-G, RF-G, SW-G, TW-G, VW-G, WF-G, WW-G and YF-G) were dissolved at a concentration of 10 µM in a redox buffer (pH 8.0, 20 mM HEPES, 150 mM NaCl, 2 mM $CaCl_2$, 3 mM NaN_3, 500 µM GSH and 125 µM GSSG). Reaction mixture was shaken at 250 rpm, 37°C for three days in a properly closed vial. Product distribution was obtained via quantitative evaluation of the HPLC-MS chromatograms.

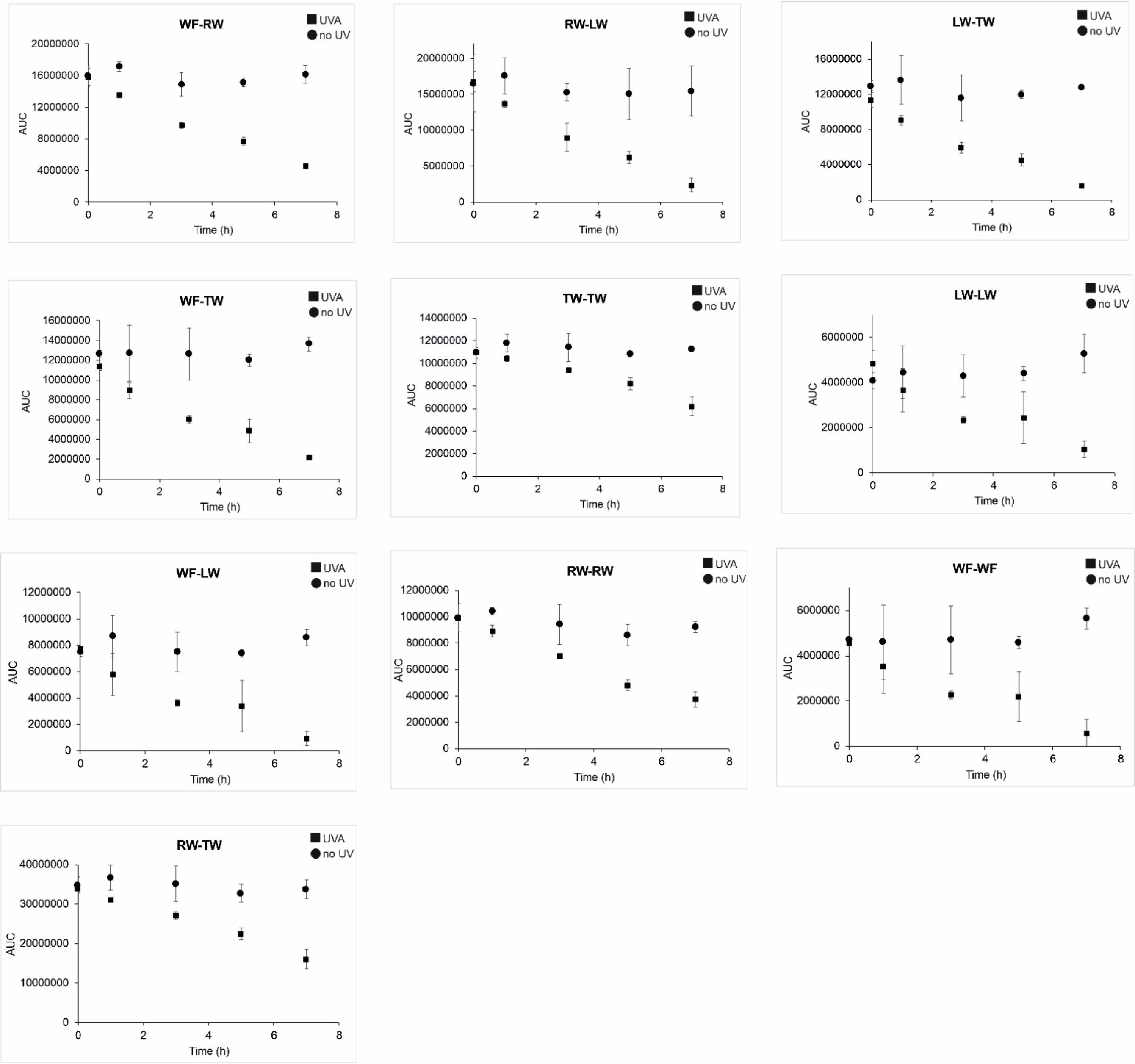

Figure S3. UVA-induced breakdown reaction of pure dimers (1 μM each) to monomers in the presence of oxidized glutathione (100 μM). The reaction was started with a system containing all 10 dimers formed by combining the subset **RW, WF, LW**, and **TW**. Dimer concentrations were 1 μM for each combination and the system contained 100 μM oxidized glutathione. The solvent conditions for the photocatalytic reactions were pH = 7.0, 20 mM HEPES, 150 mM NaCl, 2 mM $CaCl_2$ and UVA irradiation was used at 5.1 mW cm^{-2} at 356 nm (square). Control experiments were performed without UVA (circle).

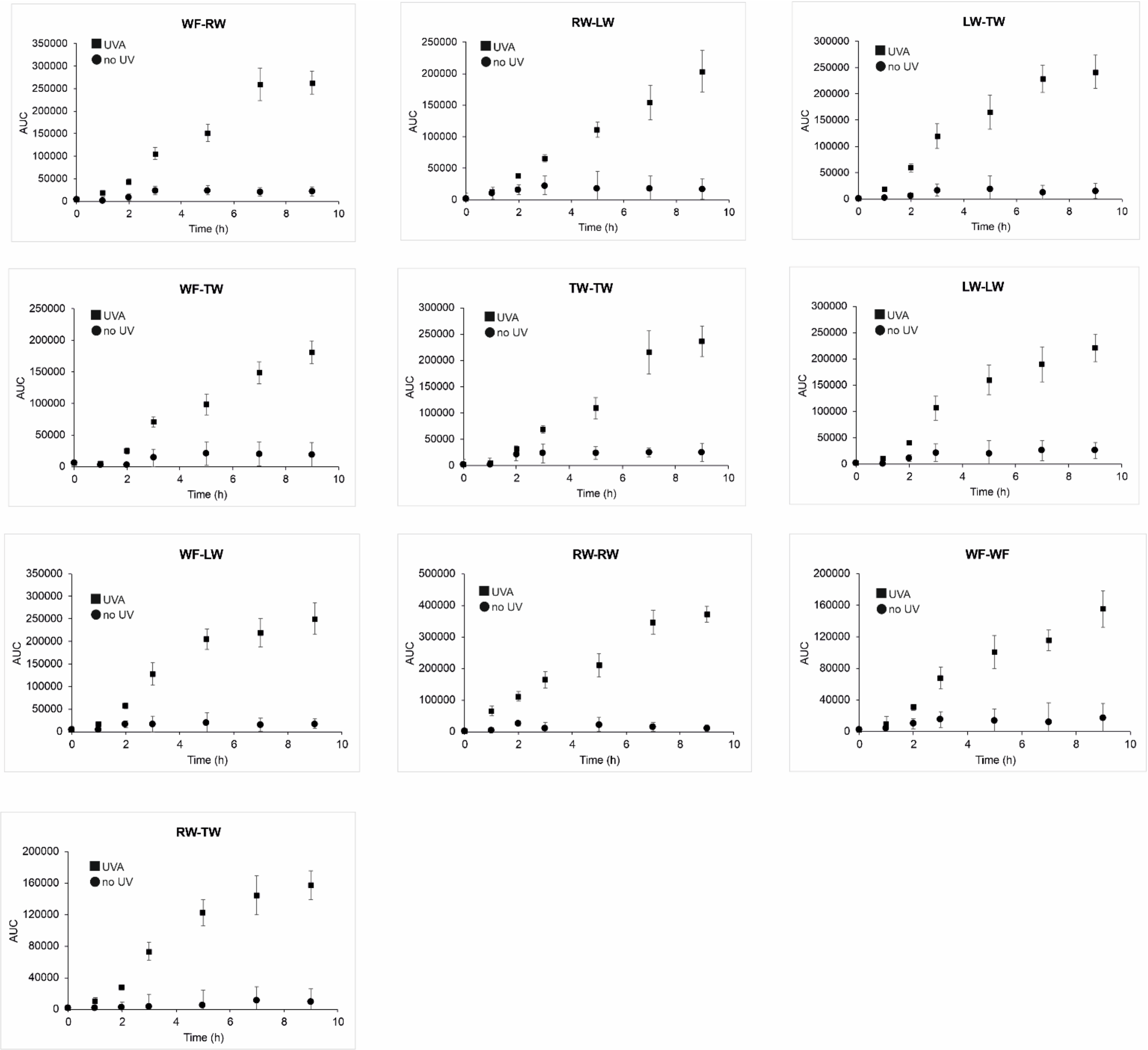

Figure S4. UVA-induced formation of dimers from monomers. Time-dependent concentration increase of the dimers as a result of UVA irradiation at 356 nm (square) and without UVA (circle). The experiment was started with pure monomers of **RW-G, WF-G, LW-G**, and **TW-G** at a concentration of 10 μM each. The solvent conditions were pH = 7.0, 20 mM HEPES, 150 mM NaCl, 2 mM $CaCl_2$ and UVA illumination was used at 5.1 mW cm^{-2}.

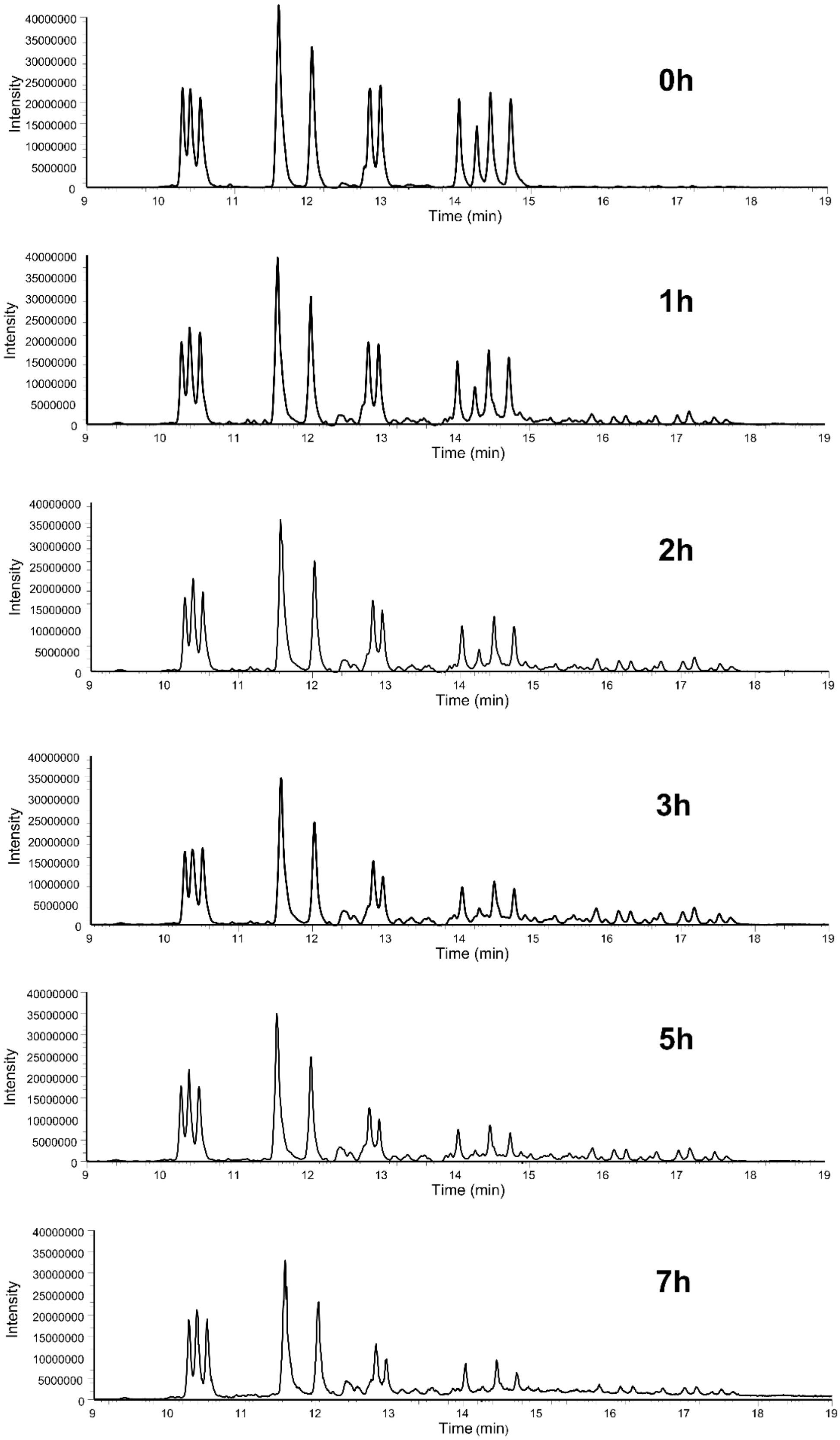

Figure S5. Representative total ion chromatograms of the foldameric mixture obtained at the power density of 5.10 mW cm^{-2}. Samples were analysed at the given time points. Conditions of the analysis: Column: Aeris Widepore XB-C18 (250 x 4.6 mm). Method: 5–80% B during 25 min, flow rate: 0.7 mL min^{-1}, where eluent A: 0.1% HCOOH in water, eluent B: 0.1% HCOOH in acetonitrile.

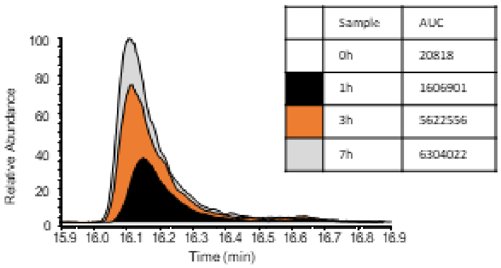

	Sample	AUC
	0h	20818
	1h	1606901
	3h	5622556
	7h	6304022

WF-YF

Mass ranges: 736-738 ($[M+3H^{+}]^{3+}$), 1104-1106 ($[M+2H^{+}]^{2+}$)

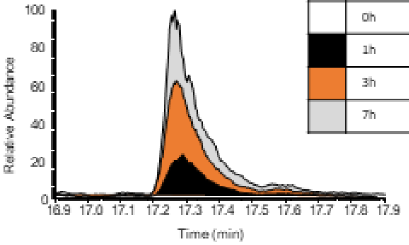

	Sample	AUC
	0h	24332
	1h	1639842
	3h	5438239
	7h	6049365

WF-IF

Mass ranges: 706-708 ($[M+3H^{+}]^{3+}$), 1059-1061 ($[M+2H^{+}]^{2+}$)

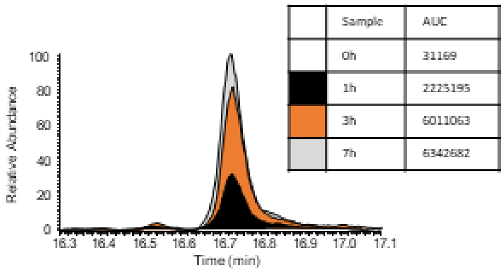

	Sample	AUC
	0h	31169
	1h	2225195
	3h	6011063
	7h	6342682

WW-IF

Mass ranges: 719-721 ($[M+3H^{+}]^{3+}$), 1079-1081 ($[M+2H^{+}]^{2+}$)

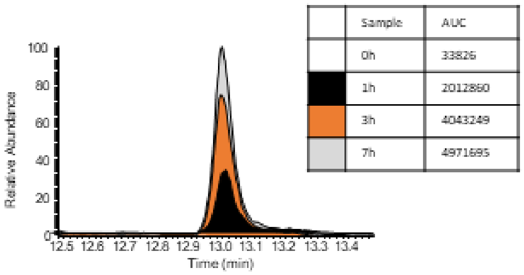

	Sample	AUC
	0h	33826
	1h	2012860
	3h	4043249
	7h	4971695

KW-TW

Mass ranges: 709-711 ($[M+3H^{+}]^{3+}$), 1063-1065 ($[M+2H^{+}]^{2+}$)

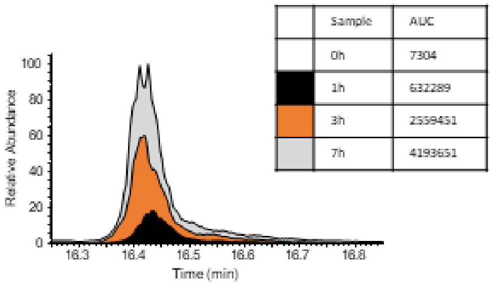

	Sample	AUC
	0h	7304
	1h	632289
	3h	2559451
	7h	4193651

YF-IF

Mass ranges: 699-701 ($[M+3H^{+}]^{3+}$), 1048-1050 ($[M+2H^{+}]^{2+}$)

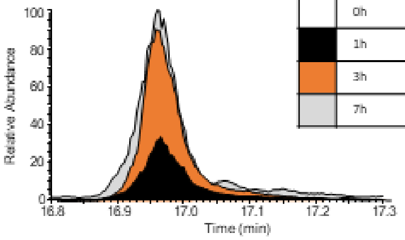

	Sample	AUC
	0h	27548
	1h	2184003
	3h	5517118
	7h	5724821

WF-WF

Mass ranges: 731-733 ($[M+3H^{+}]^{3+}$), 1096-1098 ($[M+2H^{+}]^{2+}$)

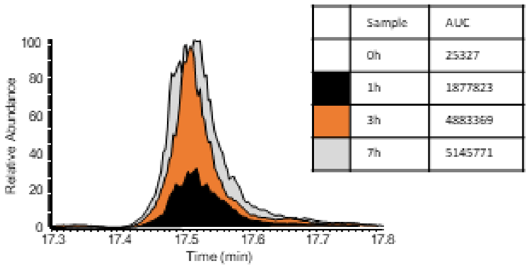

	Sample	AUC
	0h	25327
	1h	1877823
	3h	4883369
	7h	5145771

WF-LW

Mass ranges: 719-721 ($[M+3H^{+}]^{3+}$), 1079-1081 ($[M+2H^{+}]^{2+}$)

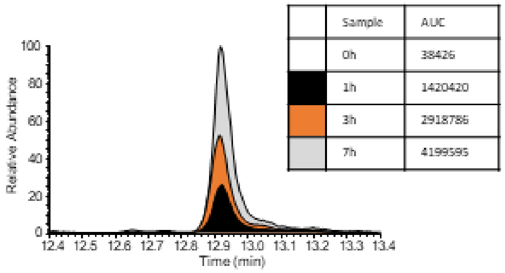

	Sample	AUC
	0h	38426
	1h	1420420
	3h	2918786
	7h	4199595

RW-QW

Mass ranges: 727-729 ($[M+3H^{+}]^{3+}$), 1091-1093 ($[M+2H^{+}]^{2+}$)

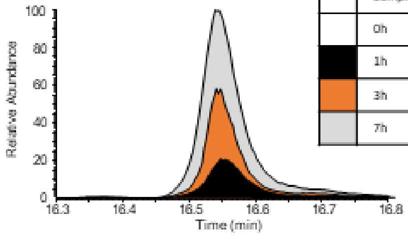

	Sample	AUC
	0h	9111
	1h	829605
	3h	2963542
	7h	3689304

YF-LW

Mass ranges: 712-714 ($[M+3H^{+}]^{3+}$), 1067-1069 ($[M+2H^{+}]^{2+}$)

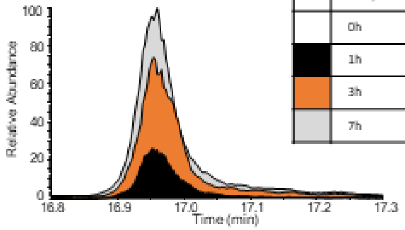

	Sample	AUC
	0h	12651
	1h	930071
	3h	3054505
	7h	3553018

WF-VW

Mass ranges: 715-717 ($[M+3H^{+}]^{3+}$), 1072-1074 ($[M+2H^{+}]^{2+}$)

Figure S6. Extracted ion chromatograms of the given representatives obtained at power density of 5.10 mW cm^{-2}. Samples were measured at different time points (after 1, 3 and 7 h, black, orange and grey, respectively), intensity of the signal was observed at the given mass-to range values.

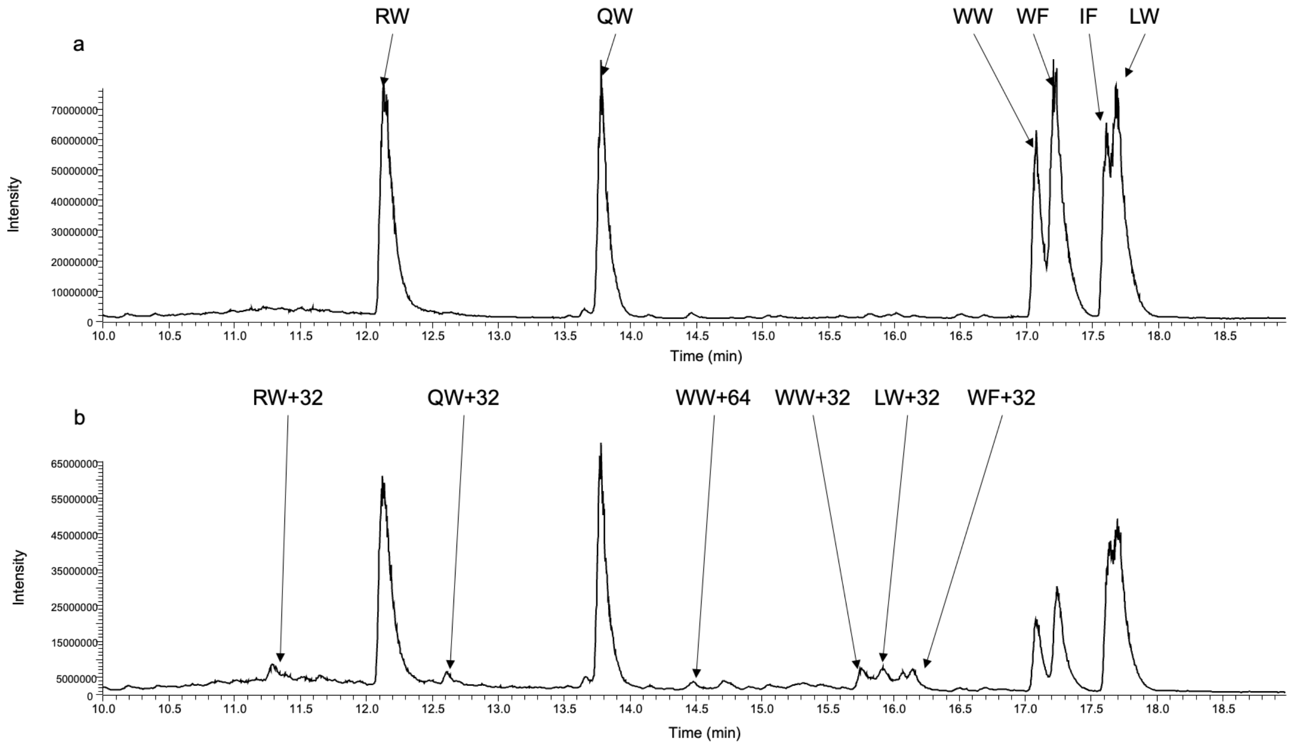

Figure S7. Chromatograms showing the importance of argon atmosphere during UVA irradiation. A mixture of six foldamers (**RW, QW, WW, WF, IF**, and **LW**) without glutathione was used at 10 μM concentration. Samples were measured on HPLC-MS after 12 hours of UVA irradiation. In accordance with the UV-induced disulfide exchange experiments, the argon atmosphere was replenished every hour in the case of (a). Foldamers were stable in the presence of argon atmosphere (a). Without argon atmosphere, UVA-induced oxidation of H14 helices was observed as tryptophan oxidation products with foldamer molecular weight +32 Da mass (b).

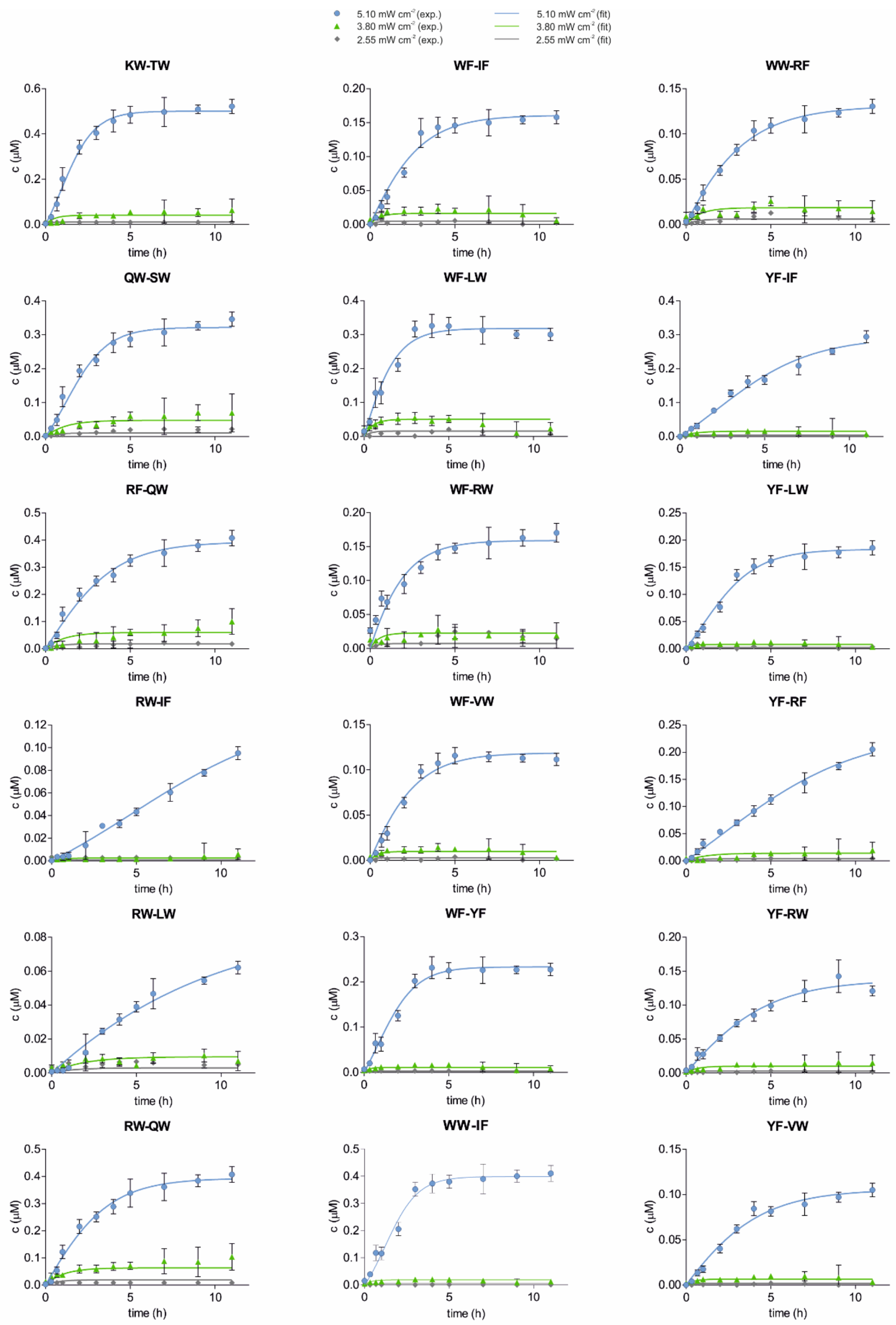

Figure S8. Fitting of the dynamic model to the light intensity and time-dependent data arrays measured for representative replicators. Data at different light intensities are represented as follows: 2.55 mW cm^{-2} (gray diamond), 3.80 mW cm^{-2} (green triangle) and 5.10 mW cm^{-2} (blue circle); fitted curves are depicted with matching color. Non-linear least-squares analysis was used for fitting. Experimental data were obtained from three parallel measurements on three different samples. See Table S2 for fitted parameters.

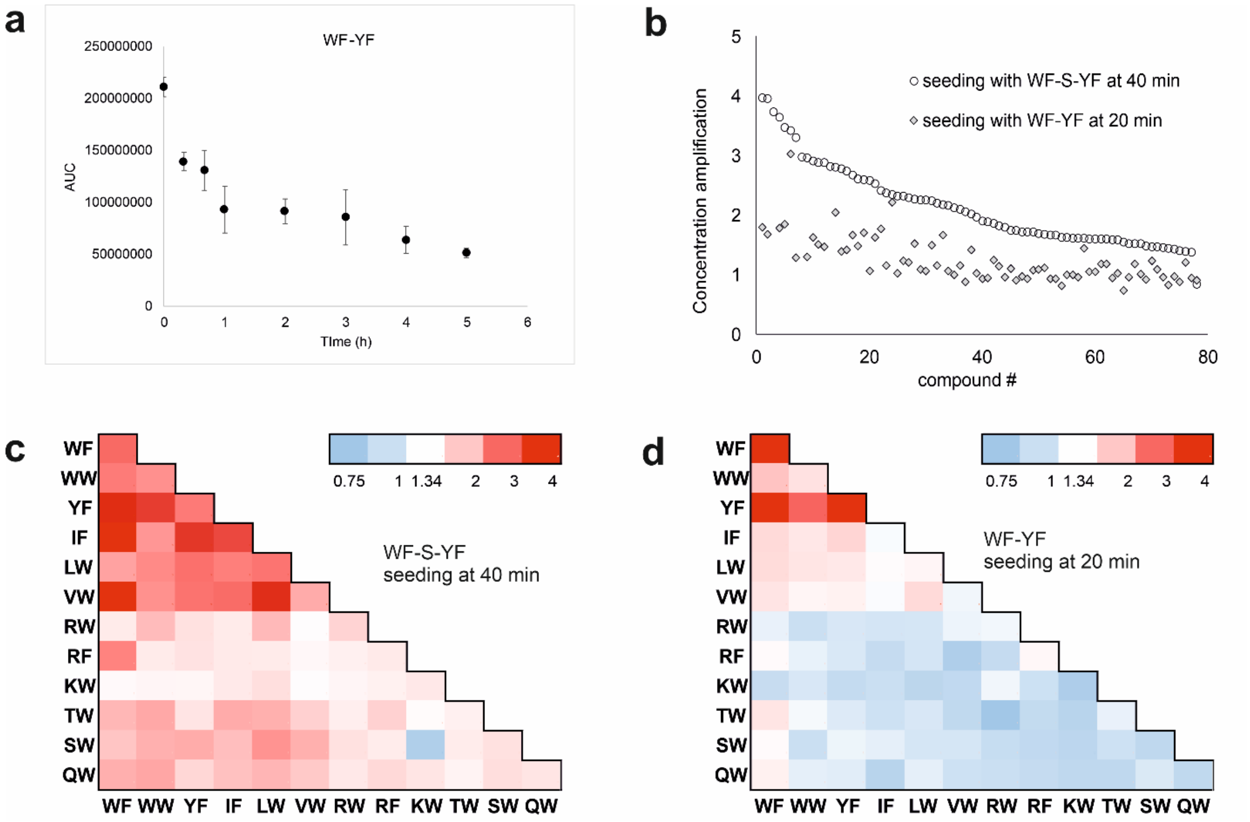

Figure S9. Seeding experiment with the disulfide dimer WF-YF. (a) WF-YF dimer break down upon UVA irradiation in the mixture containing 12 glutathion-protected monomers **IF-G, KW-G, LW-G, QW-G, RW-G, RF-G, SW-G, TW-G, VW-G, WF-G, WW-G**, and **YF-G** at 10 μM concentrations. Initial WF-YF disulfide dimer concentration was 10 μM. (b) Comparison of concentration amplification factors upon seeding with 10 μM **WF-YF** (grey diamond) and the thioether **WF-S-YF** dimers (white circles). In the case of **WF-YF**, the sequence-dependent initial rate increase proved to be higher at 20 minutes than at 40 minutes (probably due to the break down of the seed). Accordingly, amplification factors obtained at 20 minutes are represented for **WF-YF**. As **WF-YF** readily decomposed to **WF-WF** and **YF-YF**, concentration amplification factors of these dimers and that of **WF-YF** seed are not represented in the scatter plot. Heat map representations of the amplification factors upon seeding with **WF-S-YF** at 40 min (**c**) and **WF-YF** at 20 minutes (**d**).

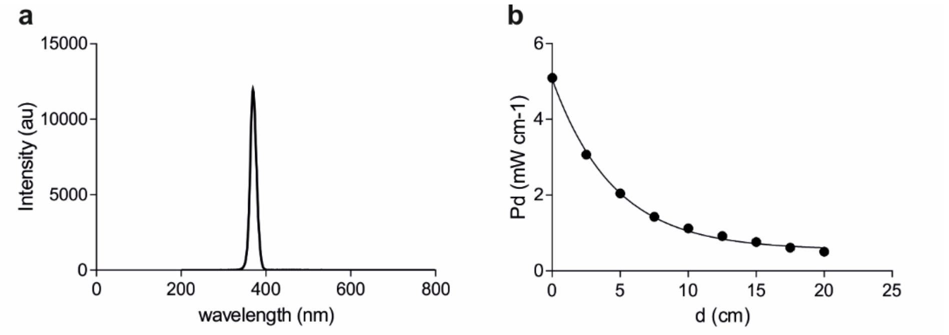

Figure S10. Calibration of the UV light source. (**a**) The emission spectrum of the UV light source (UVL-28 EL Series UV Lamp), with an emission maximum at 365 ± 5 nm and (**b**) dependence of the power density (Pd [mW cm^{-2}]) on the distance. The power density was measured with an S140C Integrating Sphere Photodiode Power Sensor (Thorlab Inc.). The wavelength range of the Si detector was 350–1100 nm, and the power range was 1 μW–500 mW (resolution: 1 nW)

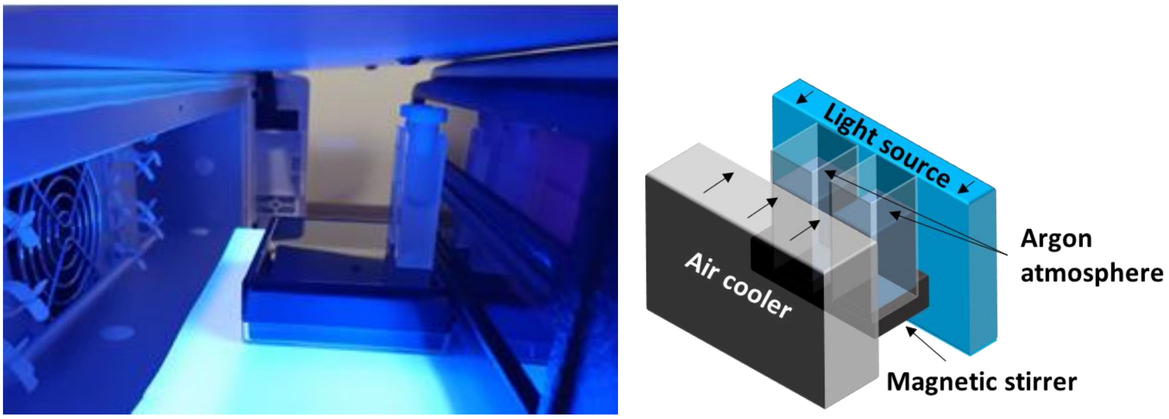

Figure S11. Photo and schematic representation of the experimental setup. The reaction mixtures were stirred at 150 RPM in quartz cuvettes having a PTFE stopper and kept under an argon atmosphere during the experiment. Constant temperature (303 ± 1 K) was maintained with *an* active air-cooling system (column thermostat). The temperature was monitored with a laser-gun thermometer. Continuous irradiation of the samples was carried out by UVL-28 EL Series UV lamp working at 365 nm (Analytic Jena US, Upland, CA), and the power density was varied by changing the distance between the light source and the samples.

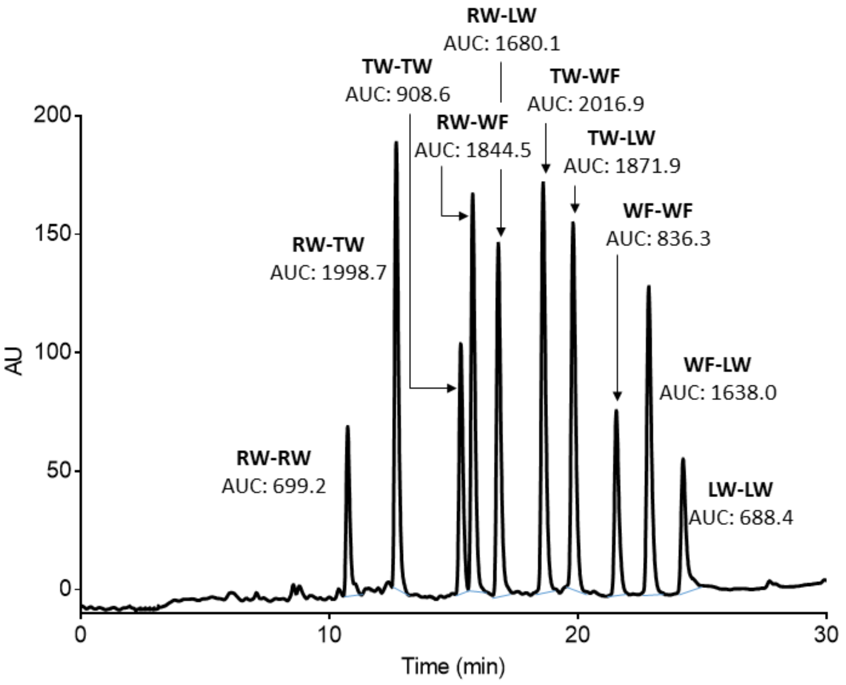

Figure S12. Product distribution obtained by slow oxidation in a chaotropic solvent. Oxidation of the glutathione-protected foldamers **WF-SG**, **LW-SG**, **RW-SG** and **TW-SG** resulted in a statistical product distribution despite their different tendencies to associate. Samples were analysed by HPLC-MS. Conditions of the analytical HPLC-MS measurement: column: Phenomenex Luna-C18 (250 x 4.6 mm); method: 5-80% B during 25 min, flow rate: 0.7 mL min^{-1}, where eluent A: 0.1% TFA in water, eluent B: 0.1% TFA in ACN: water = 8:1.

Supplementary Tables

Table S1. Concentration amplifications (CA) for the MSSM dimers upon seeding with WF-S-YF, RF-S-RW and WF-S-RW at 40 min.

	seeding with WF-S-YF at 40 min		seeding with RF-S-RW at 40 min		seeding with WF-S-RW at 40 min	
**compound#**	**$CA_{i,\ 40\ min}$**	**σ ($CA_{i,\ 40\ min}$)**	**$CA_{i,\ 40\ min}$**	**σ ($CA_{i,\ 40\ min}$)**	**$CA_{i,\ 40\ min}$**	**σ ($CA_{i,\ 40\ min}$)**
**IF-IF**	3.30	0.01	0.85	0.17	0.81	0.14
**IF-QW**	2.06	0.07	0.92	0.08	0.77	0.11
**IF-SW**	2.10	0.09	0.95	0.09	0.80	0.16
**IF-TW**	2.32	0.01	0.93	0.10	0.78	0.08
**IF-VW**	2.96	0.08	0.94	0.26	0.79	0.10
**KW-IF**	1.62	0.06	0.98	0.08	0.82	0.13
**KW-KW**	1.63	0.21	3.40	0.64	1.09	0.27
**KW-LW**	1.74	0.01	0.98	0.06	0.89	0.20
**KW-QW**	1.67	0.10	0.99	0.04	0.94	0.26
**KW-RF**	1.52	0.16	2.19	0.61	0.67	0.30
**KW-SW**	0.84	0.16	1.01	0.07	0.94	0.26
**KW-TW**	1.41	0.19	1.31	0.27	0.93	0.33
**KW-VW**	1.38	0.03	0.98	0.06	0.90	0.25
**LW-IF**	2.78	0.29	0.87	0.20	0.79	0.17
**LW-LW**	2.88	0.22	0.92	0.25	0.80	0.17
**LW-QW**	2.20	0.20	0.89	0.03	0.84	0.23
**LW-SW**	2.58	0.13	0.90	0.09	0.87	0.23
**LW-TW**	2.26	0.14	0.96	0.03	0.79	0.10
**LW-VW**	3.65	0.06	1.02	0.39	0.84	0.12
**QW-QW**	1.67	0.14	0.96	0.14	0.83	0.12
**RF-IF**	1.61	0.11	0.99	0.06	0.83	0.19
**RF-LW**	1.60	0.15	1.00	0.09	0.90	0.13
**RF-QW**	1.80	0.09	1.01	0.06	0.97	0.33
**RF-RF**	1.61	0.23	2.44	0.21	0.92	0.27
**RF-SW**	1.59	0.01	1.03	0.15	0.86	0.21
**RF-TW**	1.89	0.09	1.00	0.08	0.95	0.29
**RF-VW**	1.45	0.04	0.93	0.11	0.89	0.24
**RW-IF**	1.60	0.15	0.97	0.05	0.86	0.07
**RW-KW**	1.47	0.23	2.50	0.83	0.88	0.14
**RW-LW**	2.16	0.43	0.98	0.11	1.36	0.42
**RW-QW**	1.63	0.05	1.34	0.32	0.86	0.27
**RW-RF**	1.53	0.13	1.52	0.49	1.14	0.41
**RW-RW**	1.87	0.35	1.87	0.41	1.04	0.22
**RW-SW**	1.72	0.04	1.12	0.27	0.94	0.19
**RW-TW**	1.55	0.06	0.99	0.12	0.87	0.19
**RW-VW**	1.39	0.28	1.03	0.03	0.82	0.26
**SW-QW**	1.75	0.02	0.95	0.06	0.87	0.16
**SW-SW**	1.72	0.09	0.94	0.06	1.00	0.34
**TW-QW**	1.49	0.04	0.93	0.04	0.79	0.08
**TW-SW**	1.59	0.02	0.94	0.05	0.86	0.08
**TW-TW**	1.53	0.03	1.00	0.06	0.80	0.17
**VW-QW**	1.97	0.02	0.94	0.08	0.83	0.19
**VW-SW**	2.26	0.31	1.00	0.10	0.87	0.27
**VW-TW**	1.90	0.09	0.97	0.10	0.83	0.20
**VW-VW**	2.32	0.02	0.95	0.21	0.83	0.04
**WF-IF**	3.97	0.26	0.90	0.23	0.75	0.12
**WF-KW**	1.43	0.04	0.91	0.03	0.96	0.30
**WF-LW**	2.41	0.13	0.93	0.19	0.77	0.11
**WF-QW**	2.27	0.12	0.86	0.04	0.91	0.27
**WF-RF**	2.74	0.06	0.91	0.08	1.09	0.37
**WF-RW**	1.60	0.13	0.93	0.03	0.83	0.16
**WF-SW**	2.02	0.37	0.96	0.14	0.87	0.31
**WF-TW**	2.18	0.06	0.93	0.06	0.78	0.14
**WF-VW**	3.96	0.09	0.92	0.23	0.74	0.09
**WF-WF**	2.98	0.10	0.92	0.13	0.73	0.08
**WF-WW**	2.81	0.16	0.87	0.11	0.75	0.08

**WF-YF**	3.73	0.12	0.87	0.11	0.70	0.20
**WW-IF**	2.53	0.04	0.90	0.13	0.73	0.13
**WW-KW**	1.47	0.03	1.15	0.19	0.78	0.17
**WW-LW**	2.67	0.13	0.96	0.26	0.76	0.12
**WW-QW**	2.37	0.02	0.87	0.07	0.80	0.14
**WW-RF**	1.60	0.04	0.97	0.03	0.84	0.18
**WW-RW**	2.13	0.30	1.04	0.21	0.83	0.14
**WW-SW**	2.25	0.00	0.86	0.07	0.71	0.20
**WW-TW**	2.35	0.01	0.88	0.09	0.72	0.06
**WW-VW**	2.61	0.09	0.88	0.17	0.73	0.09
**WW-WW**	2.60	0.12	0.87	0.09	0.75	0.09
**WW-YF**	3.42	0.06	0.92	0.12	0.78	0.16
**YF-IF**	3.48	0.20	0.91	0.18	0.77	0.03
**YF-KW**	1.46	0.23	0.98	0.07	0.85	0.22
**YF-LW**	2.91	0.08	0.94	0.24	0.76	0.01
**YF-QW**	1.82	0.06	0.96	0.05	0.78	0.18
**YF-RF**	1.70	0.06	0.96	0.10	0.82	0.22
**YF-RW**	1.72	0.07	1.01	0.03	0.85	0.20
**YF-SW**	2.30	0.08	0.96	0.01	0.65	0.11
**YF-TW**	1.68	0.00	1.00	0.06	0.94	0.25
**YF-VW**	2.89	0.05	0.91	0.14	0.77	0.10
**YF-YF**	2.82	0.03	0.90	0.11	0.75	0.01

Table S2. Fitted rate constants for representative dimers.

**Compounds**	s_{p1} M^{-1} (cm^2) W^{-1} s^{-1}	s_{p2} x 10^2 M^{-1} $(cm^2)^2$ W^{-2} s^{-1}	s_{a1} x 10^7 M^{-2} (cm^2) W^{-1} s^{-1}	s_{a2} x 10^{10} M^{-2} $(cm^2)^2$ W^{-2} s^{-1}	**b** x 10^{-2} $(cm^2)^{0.5}$ $W^{-0.5}$ s^{-1}	**RMSD** x 10^{-9} M s^{-1}
**KW-TW**	0.00	38.03	0.01	205.16	2.80	19.97
**QW-SW**	0.00	25.94	0.00	107.88	1.57	15.49
**RF-QW**	0.00	25.80	0.00	77.01	1.12	24.99
**RW-IF**	0.00	1.99	0.00	117.49	1.68	3.60
**RW-LW**	1.27	0.00	0.00	70.29	1.05	4.50
**RW-QW**	0.00	27.53	18.24	66.25	1.47	18.47
**WF-IF**	5.43	0.00	247.59	0.00	6.95	10.53
**WF-LW**	0.00	38.80	100.06	73.31	3.85	22.19
**WF-RW**	2.73	14.65	0.00	143.86	2.21	17.27
**WF-VW**	4.39	0.24	304.75	0.00	8.61	6.70
**WF-YF**	0.00	13.41	270.42	0.00	7.45	11.26
**WW-IF**	0.00	24.06	0.00	280.08	3.84	20.34
**WW-RF**	4.38	0.00	155.16	0.57	4.42	15.14
**YF-IF**	0.00	8.91	0.02	112.28	1.58	11.85
**YF-LW**	4.01	0.04	270.87	0.00	7.53	7.15
**YF-RF**	0.00	5.91	0.00	83.24	1.19	6.35
**YF-RW**	2.83	0.35	177.91	0.08	5.01	6.71
**YF-VW**	0.00	6.77	0.00	178.50	2.64	5.06

Mean standard deviation of the fitted parameters was estimated with jackknife resampling technique and it was found to be less than 3.4% in each case.
s_{p1}: rate constant of spontaneous synthesis by proximity-controlled radical substitution
s_{p2}: rate constant of spontaneous synthesis by proximity-controlled concerted metathesis
s_{a1}: rate constant of autocatalytic synthesis by proximity-controlled radical substitution
s_{a2}: rate constant of autocatalytic synthesis by proximity-controlled concerted metathesis
b: rate constant of dimers break down via diffusion-controlled radical substitution

Table S3. HPLC-MS characterisation of dimers and monomers in the system.

Compounds	Calculated molar mass (Da)	Retention time (min)[a]	Detected ions	
			$[M+2H^+]^{2+}$	$[M+3H^+]^{3+}$
**IF-IF**	2044.66	17.73	1022.95	682.29
**IF-QW**	2098.67	15.70	1049.79	700.47
**IF-G**	1327.62	14.49	664.84	443.54
**IF-SW**	2057.62	15.79	1029.89	686.75
**IF-TW**	2071.65	16.05	1036.42	691.59
**IF-VW**	2069.67	17.29	1035.41	690.82
**KW-IF**	2098.71	14.55	1049.79	700.00
**KW-KW**	2152.76	11.70	1077.40	718.48
**KW-LW**	2137.75	14.70	1069.44	713.56
**KW-QW**	2152.72	12.73	1076.93	718.90
**KW-RF**	2141.74	11.63	1071.41	714.82
**KW-G**	1381.69	10.42	691.85	461.56
**KW-SW**	2111.63	12.73	1056.87	705.04
**KW-TW**	2125.70	13.09	1063.35	709.34
**KW-VW**	2123.68	14.26	1063.34	708.73
**LW-IF**	2084.61	17.84	1042.52	695.41
**LW-LW**	2123.72	18.00	1062.03	708.28
**LW-QW**	2137.71	15.84	1069.41	713.77
**LW-G**	1366.68	14.76	684.35	456.56
**LW-SW**	2096.66	15.94	1048.90	700.19
**LW-TW**	2111.67	16.19	1056.01	704.69
**LW-VW**	2108.71	17.43	1054.60	703.40
**QW-QW**	2152.68	13.71	1077.12	718.79
**QW-G**	1381.65	11.60	691.83	461.55
**RF-IF**	2087.69	14.59	1044.38	697.05
**RF-LW**	2126.73	14.77	1063.94	709.58
**RF-QW**	2141.70	12.73	1071.37	715.15
**RF-RF**	2130.72	11.64	1066.67	710.97
**RF-G**	1370.68	10.31	686.35	457.89
**RF-SW**	2100.65	12.80	1050.89	701.43
**RF-TW**	2114.68	12.77	1057.93	706.26
**RF-VW**	2112.70	14.28	1056.99	705.03
**RW-IF**	2126.73	14.77	1064.57	709.87
**RW-KW**	2180.78	11.80	1091.33	728.04
**RW-LW**	2165.77	14.91	1083.50	722.76
**RW-QW**	2180.74	12.90	1090.87	728.20
**RW-RF**	2169.76	12.22	1085.36	724.15
**RW-RW**	2208.80	11.97	1104.90	737.47
**RW-G**	1409.69	10.56	705.85	470.90
**RW-SW**	2139.69	12.96	1070.38	714.39
**RW-TW**	2153.72	13.29	1077.46	718.90
**RW-VW**	2151.74	14.41	1076.35	718.45
**SW-QW**	2110.69	13.76	1056.41	704.63
**SW-G**	1340.63	11.60	671.32	447.88
**SW-SW**	2070.58	12.99	1035.86	690.84
**TW-QW**	2125.66	14.05	1063.25	709.33
**TW-G**	1354.64	12.06	678.33	452.55
**TW-SW**	2083.70	14.13	1042.93	695.92
**TW-TW**	2098.64	14.35	1049.99	700.56
**VW-QW**	2122.74	15.35	1063.00	708.79
**VW-G**	1352.66	14.05	677.34	451.89
**VW-SW**	2082.63	15.44	1042.40	695.14
**VW-TW**	2096.66	15.70	1049.37	699.79
**VW-VW**	2094.68	16.97	1048.03	698.88
**WF-IF**	2117.72	17.27	1059.49	706.71
**WF-KW**	2171.77	14.31	1086.48	724.64
**WF-LW**	2156.76	17.45	1078.90	719.64
**WF-QW**	2171.73	15.40	1086.93	724.91
**WF-RF**	2160.75	14.37	1081.34	721.03
**WF-RW**	2199.79	14.48	1100.43	734.51
**WF-G**	1400.66	14.29	701.34	467.89
**WF-SW**	2130.68	15.49	1065.95	711.30
**WF-TW**	2144.71	15.76	1073.20	715.93
**WF-VW**	2142.73	16.96	1071.97	715.50
**WF-WF**	2190.78	16.96	1095.93	731.76
**WF-WW**	2229.81	16.41	1115.44	744.44
**WF-YF**	2167.74	16.10	1084.52	723.76
**WW-IF**	2156.75	16.78	1078.99	719.84
**WW-KW**	2210.80	13.73	1105.93	738.15
**WW-LW**	2195.79	16.89	1098.46	732.71
**WW-QW**	2210.76	14.71	1105.98	737.87
**WW-RF**	2199.78	14.48	1100.43	734.51
**WW-RW**	2238.82	13.94	1120.36	747.47
**WW-G**	1439.67	12.86	720.84	480.89
**WW-SW**	2169.71	16.08	1086.53	725.38
**WW-TW**	2183.74	15.09	1092.55	728.13
**WW-VW**	2181.76	16.39	1091.41	728.06
**WW-WW**	2268.84	15.81	1135.45	757.47
**WW-YF**	2206.77	15.53	1103.89	736.72
**YF-IF**	2094.68	16.41	1047.94	699.11

**YF-KW**	2148.73	13.54	1075.00	717.42
**YF-LW**	2133.72	16.57	1067.86	712.64
**YF-QW**	2148.69	14.50	1074.93	717.02
**YF-RF**	2137.71	13.54	1069.47	713.51
**YF-RW**	2176.75	13.71	1088.93	726.82
**YF-G**	1377.66	12.98	689.83	460.22
**YF-SW**	2107.64	14.58	1054.34	703.55
**YF-TW**	2121.67	14.84	1061.45	707.98
**YF-VW**	2119.69	16.06	1060.47	707.47
**YF-YF**	2144.70	15.22	1073.30	715.89

[a]Analytical HPLC-MS measurement. Column: Aeris Widepore XB-C18 (250 x 4.6 mm). Method: 5–80% B during 25 min, flow rate: 0.7 mL min^{-1}, where eluent A: 0.1% HCOOH in water, eluent B: 0.1% HCOOH in acetonitrile.

Table S4. Characterisation of the glutathione-protected monomers.

**Compounds**	**Exact mass (Da)**	**Detected ions**		**Retention time (min)[a]**	**Retention time (min)[b]**
		$[M+1H^+]^{1+}$	$[M+2H^+]^{2+}$		
**IF-G**	1327.62	1328.67	664.84	20.81	14.49
**KW-G**	1381.69	1382.69	691.85	16.58	10.42
**LW-G**	1366.68	1367.68	684.35	21.79	14.76
**QW-G**	1381.65	1382.65	691.83	17.48	11.60
**RF-G**	1370.68	1371.69	686.35	16.62	10.31
**RW-G**	1409.69	1410.7	705.85	18.03	10.56
**SW-G**	1340.63	1341.63	671.32	17.23	11.60
**TW-G**	1354.64	1355.65	678.33	19.15	12.06
**VW-G**	1352.66	1353.67	677.34	20.11	14.05
**WF-G**	1400.66	1401.67	701.34	21.37	14.29
**WW-G**	1439.67	1440.68	720.84	17.23	12.86
**YF-G**	1377.66	1378.65	689.83	18.94	12.98

[a]Analytical HPLC-UV measurement. Column: Phenomenex Luna C18 (250 x 4.6 mm). Method: 5–80% B during 25 min, flow rate: 1.2 mL min^{-1}, where eluent A: 0.1% TFA in water, eluent B: 0.1% TFA and 80% ACN in water.
[b]Analytical HPLC-MS measurement. Column: Aeris Widepore XB-C18 (250 x 4.6 mm). Method: 5–80% B during 25 min, flow rate: 0.7 mL min^{-1}, where eluent A: 0.1% HCOOH in water, eluent B: 0.1% HCOOH in ACN.

Table S5. Calibration of the MS Area Under Curve (AUC) to concentration conversion for each component of the system.

**Compounds**	**calculated concentration (μM)[a]**	**mean AUC**	**SD**	**Conversion factor: AUC/μM**
**IF-IF**	0.2083	6738896	167227	**32346754**
**IF-QW**	0.4167	14321621	1021416	**34371864**
**IF-G**	5	233796364	12121312	**46759273**
**IF-SW**	0.4167	10250563	267887	**24601332**
**IF-TW**	0.4167	9781464	99474	**23475495**
**IF-VW**	0.4167	14658581	333948	**35180567**
**KW-IF**	0.4167	6984610	192959	**16763051**
**KW-KW**	0.2083	4688541	196654	**22505032**
**KW-LW**	0.4167	10931447	352192	**26235452**
**KW-QW**	0.4167	9592254	191099	**23021391**
**KW-RF**	0.4167	6041711	280144	**14500094**
**KW-G**	5	93498264	8075947	**18699653**
**KW-SW**	0.4167	10307687	34078	**24738429**
**KW-TW**	0.4167	11140758	215090	**26737798**
**KW-VW**	0.4167	11219383	61459	**26926498**
**LW-IF**	0.4167	13317251	59335	**31961377**
**LW-LW**	0.2083	5459491	232775	**26205601**
**LW-QW**	0.4167	7510246	171240	**18024575**
**LW-G**	5	71564374	1943793	**14312875**
**LW-SW**	0.4167	7846422	820359	**18831398**
**LW-TW**	0.4167	7877221	1158047	**18905315**
**LW-VW**	0.4167	11755384	350708	**28212900**
**QW-QW**	0.2083	2505601	113767	**12026904**
**QW-G**	5	154788096	3212397	**30957619**
**RF-IF**	0.4167	9685531	98651	**23245255**
**RF-LW**	0.4167	9423472	313860	**22616315**
**RF-QW**	0.4167	5395324	470128	**12948767**
**RF-RF**	0.2083	2600196	66430	**12480959**
**RF-G**	5	76355793	557309	**15271159**
**RF-SW**	0.4167	8404764	40877	**20171417**
**RF-TW**	0.4167	6458569	88664	**15500554**
**RF-VW**	0.4167	9015525	232567	**21637242**
**RW-IF**	0.4167	9139403	235650	**21934550**
**RW-KW**	0.4167	7268733	135533	**17444946**
**RW-LW**	0.4167	9690533	135870	**23257261**
**RW-QW**	0.4167	8128642	168034	**19508725**
**RW-RF**	0.4167	4839118	14804	**11613873**
**RW-RW**	0.2083	2840665	146521	**13635212**
**RW-G**	5	81307536	3537250	**16261507**
**RW-SW**	0.4167	9262117	211359	**22229063**
**RW-TW**	0.4167	5594796	220034	**13427499**
**RW-VW**	0.4167	10268484	436026	**24644341**

**SW-QW**	0.4167	7741284	52795	**18579067**
**SW-G**	5	214373675	971923	**42874735**
**SW-SW**	0.2083	4460048	9425	**21408267**
**TW-QW**	0.4167	6258758	7940	**15021007**
**TW-G**	5	205650252	4735772	**41130050**
**TW-SW**	0.4167	7866053	65835	**18878512**
**TW-TW**	0.2083	6984610	192959	**33526183**
**VW-QW**	0.4167	8395542	149336	**20149285**
**VW-G**	5	218439667	209807	**43687933**
**VW-SW**	0.4167	9549648	30622	**22919137**
**VW-TW**	0.4167	14321621	1021416	**34371864**
**VW-VW**	0.2083	6719439	16227	**32253357**
**WF-IF**	0.4167	16802806	261057	**40326702**
**WF-KW**	0.4167	12948964	294491	**31077488**
**WF-LW**	0.4167	14057330	138666	**33737565**
**WF-QW**	0.4167	9308290	140062	**22339878**
**WF-RF**	0.4167	9750493	108676	**23401163**
**WF-RW**	0.4167	5231109	21871	**12554652**
**WF-G**	5	229629674	1234010	**45925935**
**WF-SW**	0.4167	10201405	81613	**24483352**
**WF-TW**	0.4167	10261229	335150	**24626930**
**WF-VW**	0.4167	12943413	2777026	**31064167**
**WF-WF**	0.2083	9182636	141237	**44076722**
**WF-WW**	0.4167	7037382	357237	**16889704**
**WF-YF**	0.4167	11977596	184803	**28746208**
**WW-IF**	0.4167	6697741	203025	**16074566**
**WW-KW**	0.4167	7218262	137235	**17323816**
**WW-LW**	0.4167	6808518	206582	**16340430**
**WW-QW**	0.4167	4956501	97189	**11895592**
**WW-RF**	0.4167	5597430	97043	**13433822**
**WW-RW**	0.4167	5744039	55944	**13785683**
**WW-G**	5	154424106	809265	**30884821**
**WW-SW**	0.4167	7736738	259061	**18568158**
**WW-TW**	0.4167	6666086	237986	**15998593**
**WW-VW**	0.4167	6666086	237986	**15998593**
**WW-WW**	0.2083	2408501	253201	**11560822**
**WW-YF**	0.4167	4863880	87381	**11673302**
**YF-IF**	0.4167	8352642	1717484	**20046325**
**YF-KW**	0.4167	8037313	69720	**19289535**
**YF-LW**	0.4167	9072627	56896	**21774286**
**YF-QW**	0.4167	6032798	175591	**14478703**
**YF-RF**	0.4167	6941704	43753	**16660077**
**YF-RW**	0.4167	7672105	108675	**18413037**
**YF-G**	5	177069749	1736459	**35413950**
**YF-SW**	0.4167	6309200	101256	**15142069**
**YF-TW**	0.4167	6577118	44472	**15785071**
**YF-VW**	0.4167	10338711	140452	**24812887**
**YF-YF**	0.2083	2466209	13733	**11837825**

[a]Calculated from the statistical product distribution

Peptide characterisation data

HPLC and MS characterization of the individual monomeric sequences

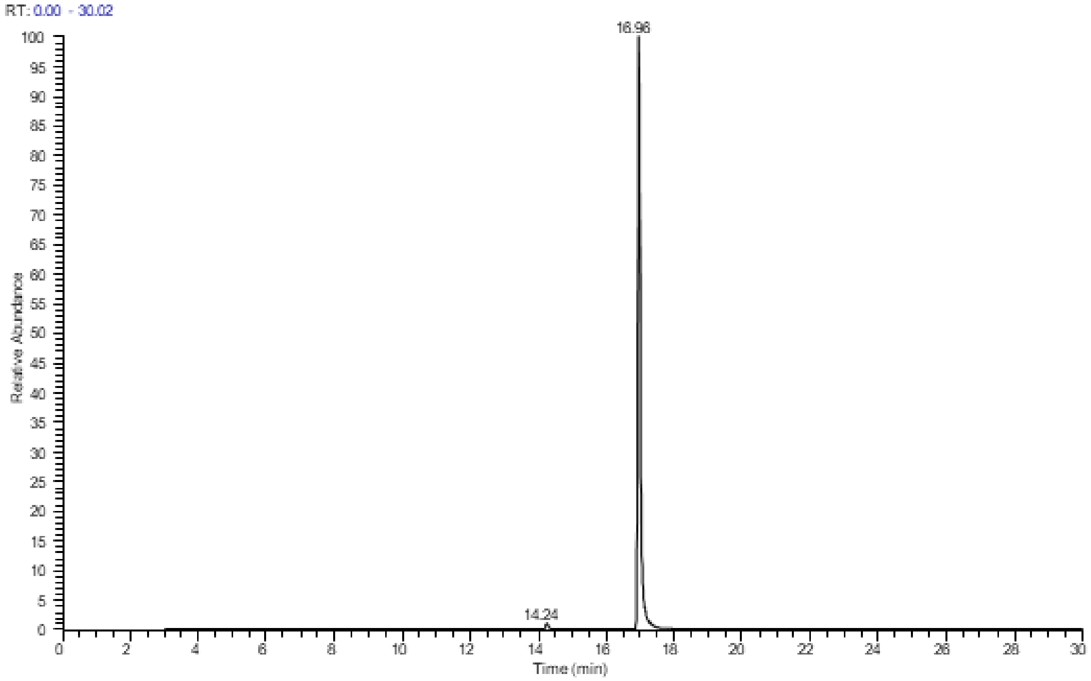

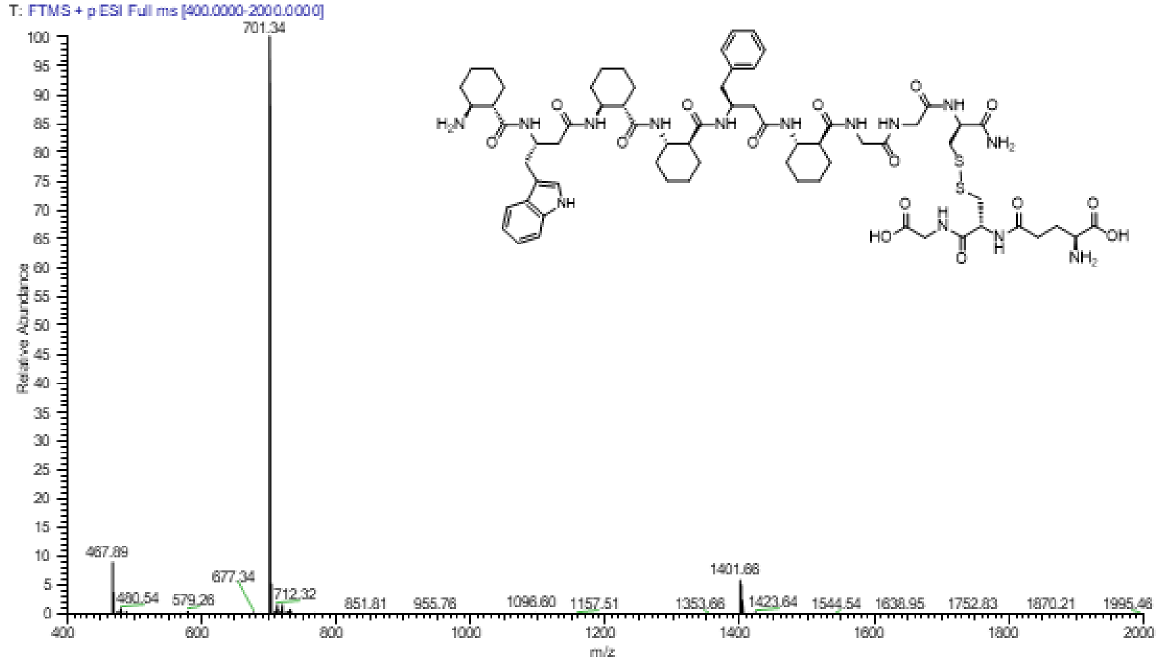

HPLC trace and mass spectrum of the glutathionic precursor **WF-G** (exact mass: 1400.66). Calculated isotopic profile for $[M+2H]^{2+}$ (species, abundance): 701.3383 (100%), 701.8400 (72.47%), 702.3417 (25.86%), 702.8379 (6.48%); m/z calculated: 1401.67 $[M+H]^{+}$, 701.24 $[M+2H]^{2+}$; m/z observed: 1401.06 $[M+H]^{+}$, 701.34 $[M+2H]^{2+}$

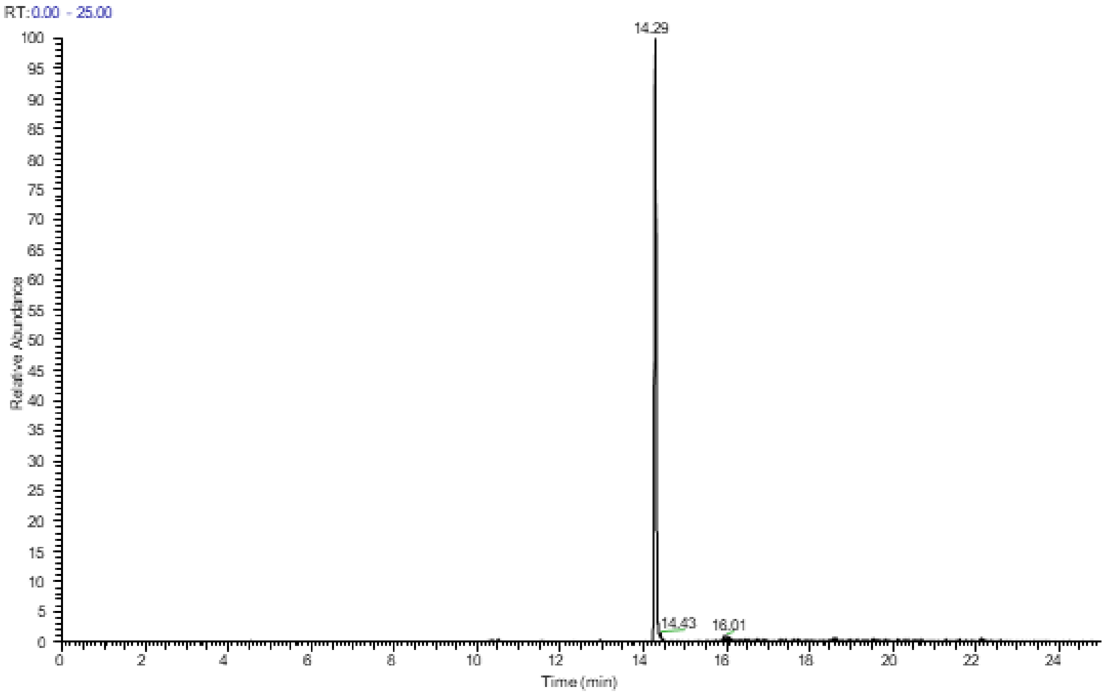

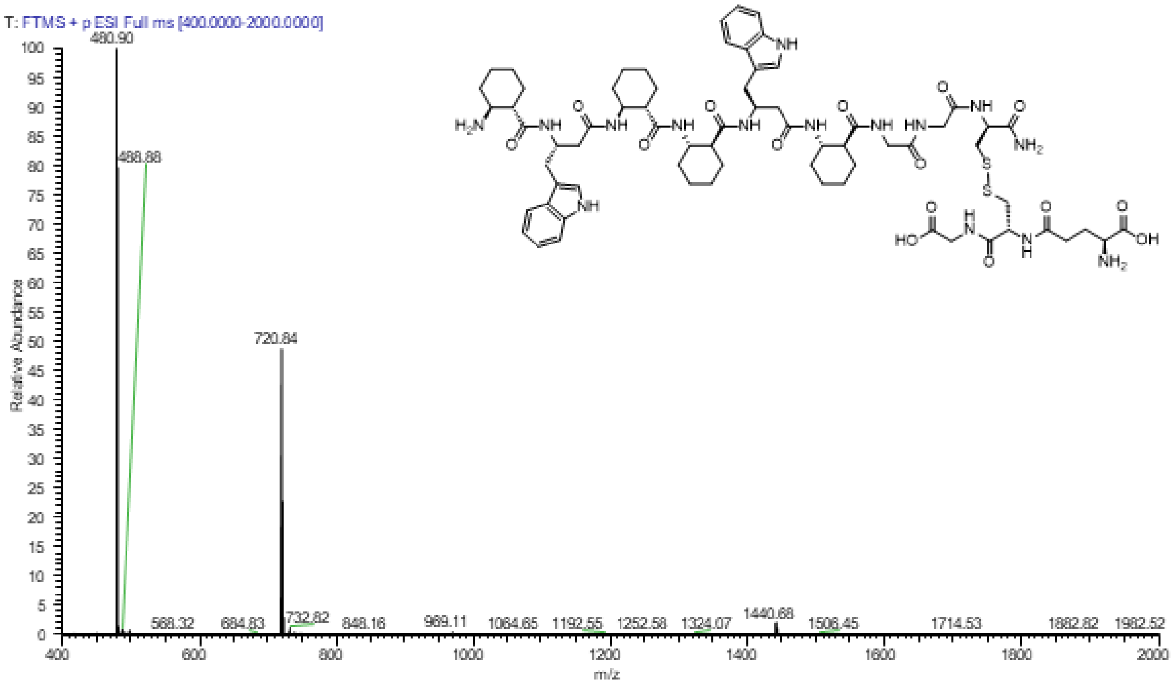

HPLC trace and mass spectrum of the glutathionic precursor **WW-G** (exact mass: 1439.67). Calculated isotopic profile for $[M+2H]^{2+}$ (species, abundance): 720.8438 (100%), 721.3454 (74.63%), 721.8471 (27.44%), 722.3434 (6.78%); m/z calculated: 1440.51 $[M+H]^{+}$, 720.76 $[M+2H]^{2+}$, 480.84 $[M+3H]^{3+}$; m/z observed: 1440.68 $[M+H]^{+}$, 720.64 $[M+2H]^{2+}$, 480.90 $[M+3H]^{3+}$

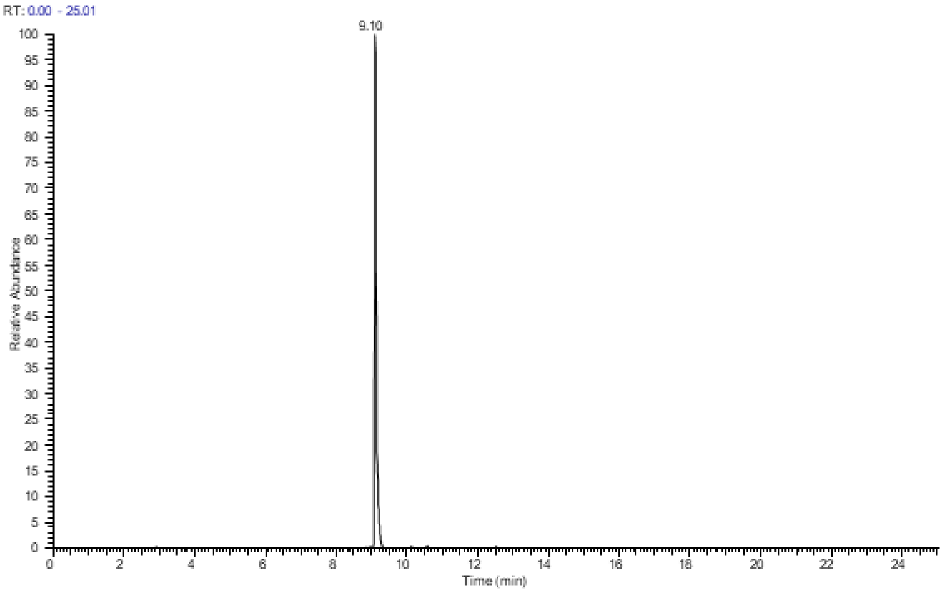

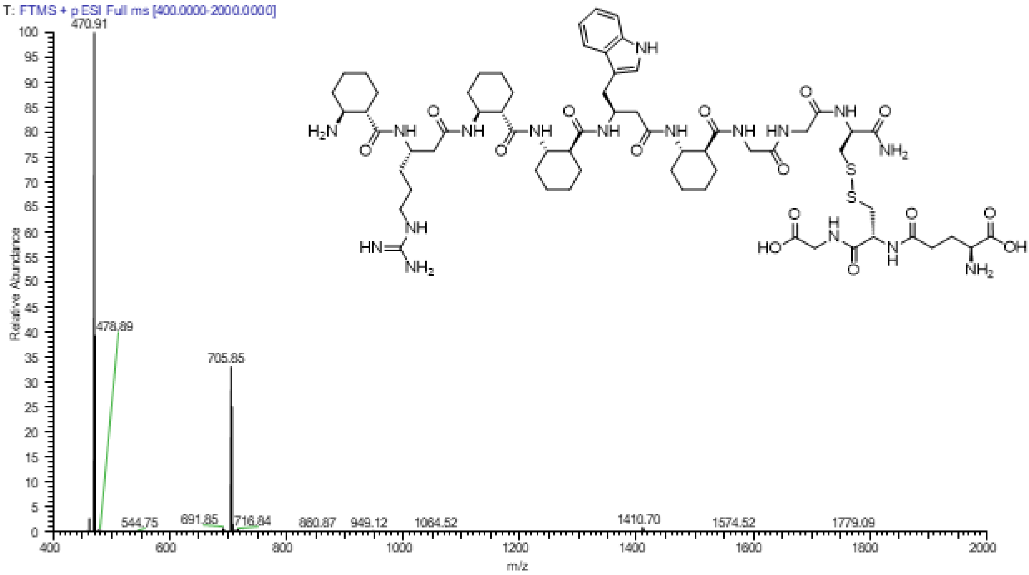

HPLC trace and mass spectrum of the glutathionic precursor **RW-G** (exact mass: 1409.69). Calculated isotopic profile for $[M+2H]^{2+}$ (species, abundance): 705.3508 (100%), 705.8524 (69.22%), 706.3487 (8.95%), 706.3510 (4.30%); m/z calculated: 1410.49 $[M+H]^{+}$, 705.75 $[M+2H]^{2+}$, 470.83 $[M+3H]^{3+}$; m/z observed: 1410.70 $[M+H]^{+}$, 705.85 $[M+2H]^{2+}$, 470.91 $[M+3H]^{3+}$

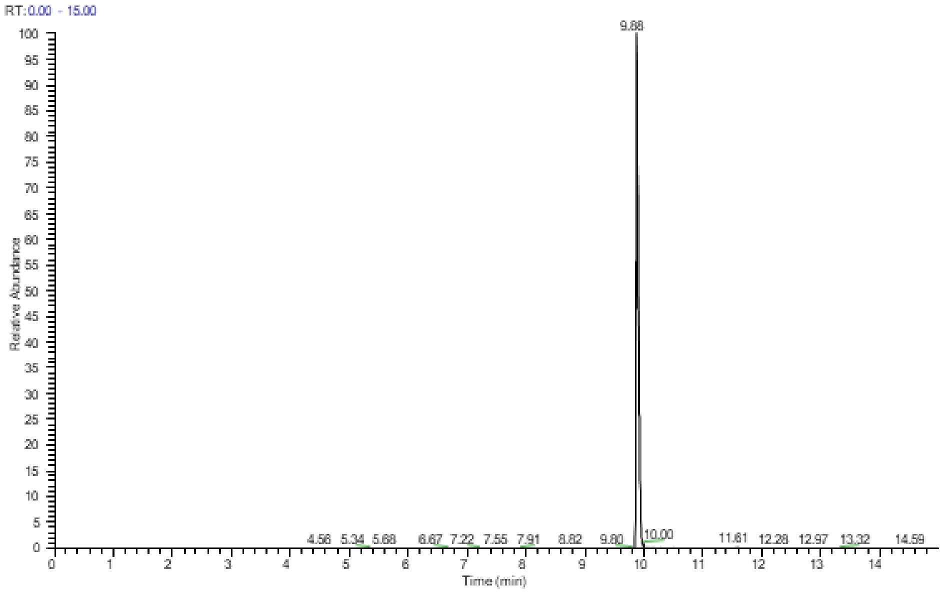

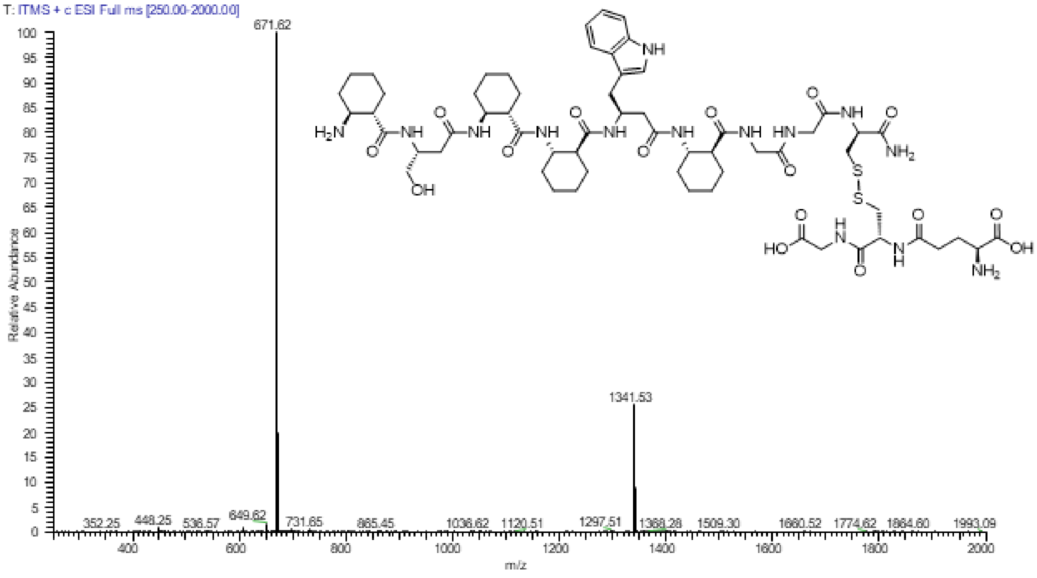

HPLC trace and mass spectrum of the glutathionic precursor **SW-G** (exact mass: 1340.63). Calculated isotopic profile for $[M+2H]^{2+}$ (species, abundance): 670.8162 (100%), 671.3179 (65.98%), 671.8141 (8.95%), 671.8164 (3.37%); m/z calculated: 1341.38 $[M+H]^{+}$, 671.19 $[M+2H]^{2+}$; m/z observed: 1341.53 $[M+H]^{+}$, 671.62 $[M+2H]^{2+}$

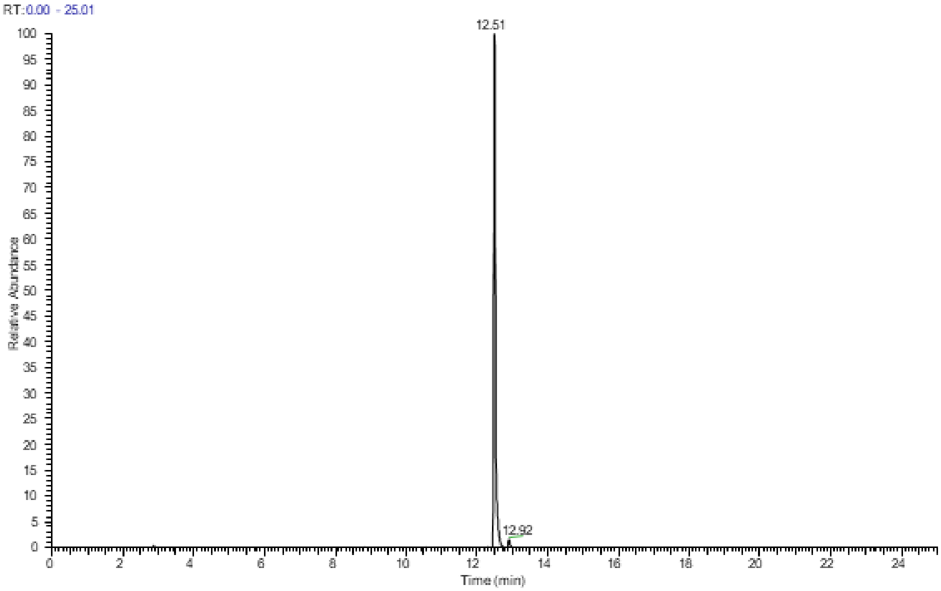

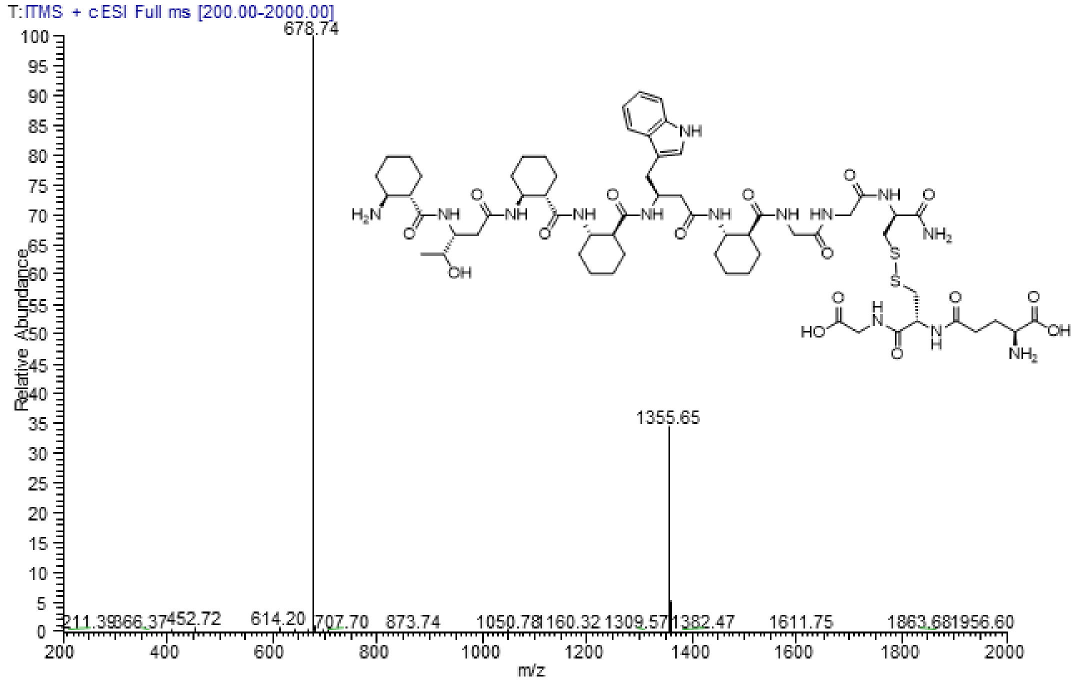

HPLC trace and mass spectrum of the glutathionic precursor **TW-G** (exact mass: 1354.64). Calculated isotopic profile for $[M+2H]^{2+}$ (species, abundance): 677.8240 (100%), 678.3257 (67.06%), 678.8211 (8.95%), 678.8242 (3.43%); m/z calculated: 1355.41 $[M+H]^{+}$, 678.21 $[M+2H]^{2+}$; m/z observed: 1355.65 $[M+H]^{+}$, 678.74 $[M+2H]^{2+}$

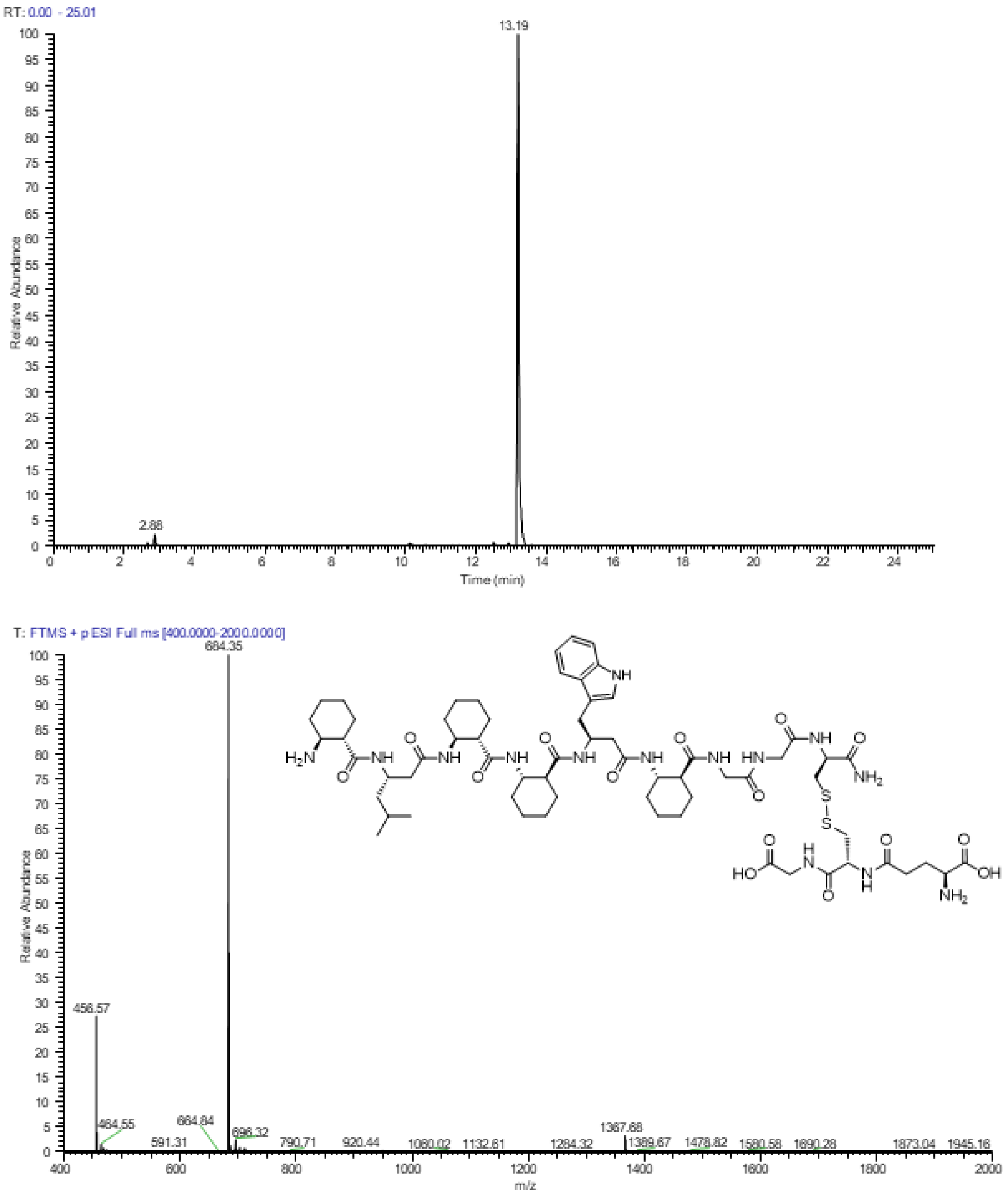

HPLC trace and mass spectrum of the glutathionic precursor **LW-G** (exact mass: 1366.68). Calculated isotopic profile for $[M+2H]^{2+}$ (species, abundance): 683.8422 (100%), 684.3439 (69.22%), 684.8401 (8.95%), 684.8424 (3.54%); m/z calculated: 1367.46 $[M+H]^{+}$, 684.23 $[M+2H]^{2+}$; m/z observed: 1367.68 $[M+H]^{+}$, 684.35 $[M+2H]^{2+}$

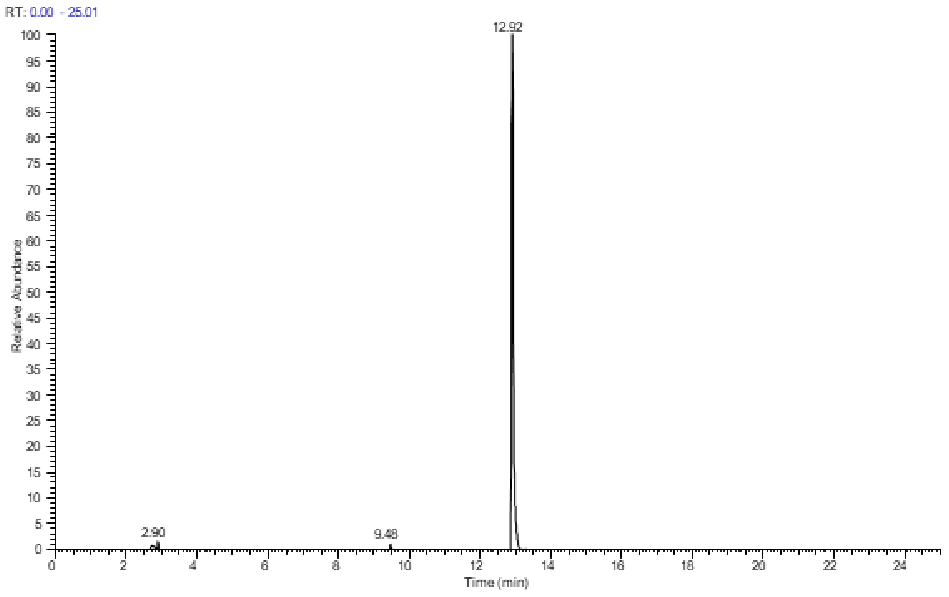

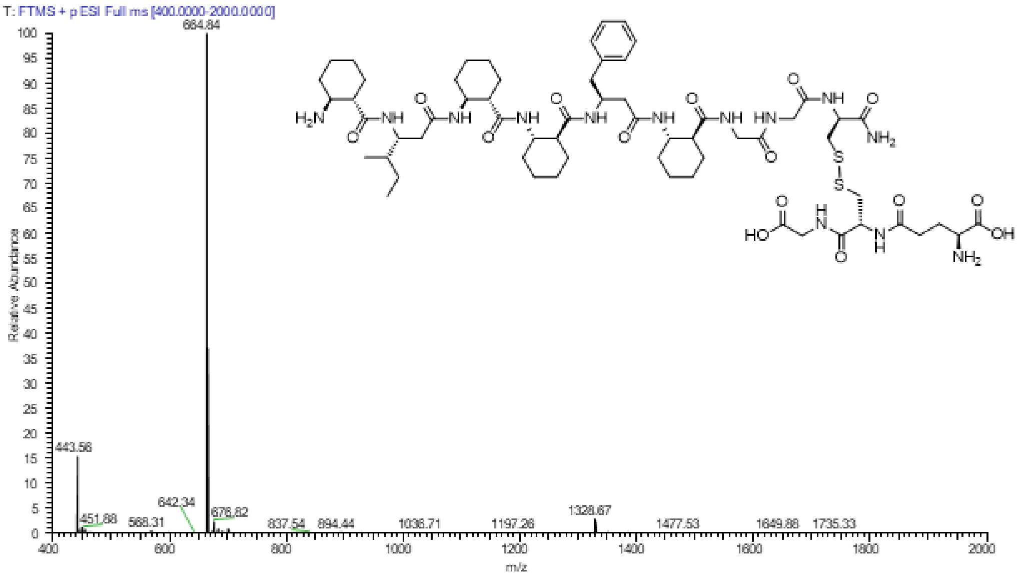

HPLC trace and mass spectrum of the glutathionic precursor **IF-G** (exact mass: 1327.67). Calculated isotopic profile for $[M+2H]^{2+}$ (species, abundance): 664.3468 (100%), 664.8385 (67.06%), 665.3347 (8.95%), 665.3370 (3.18%); m/z calculated: 1328.42 $[M+H]^{+}$, 664.71 $[M+2H]^{2+}$; m/z observed: 1328.67 $[M+H]^{+}$, 664.84 $[M+2H]^{2+}$

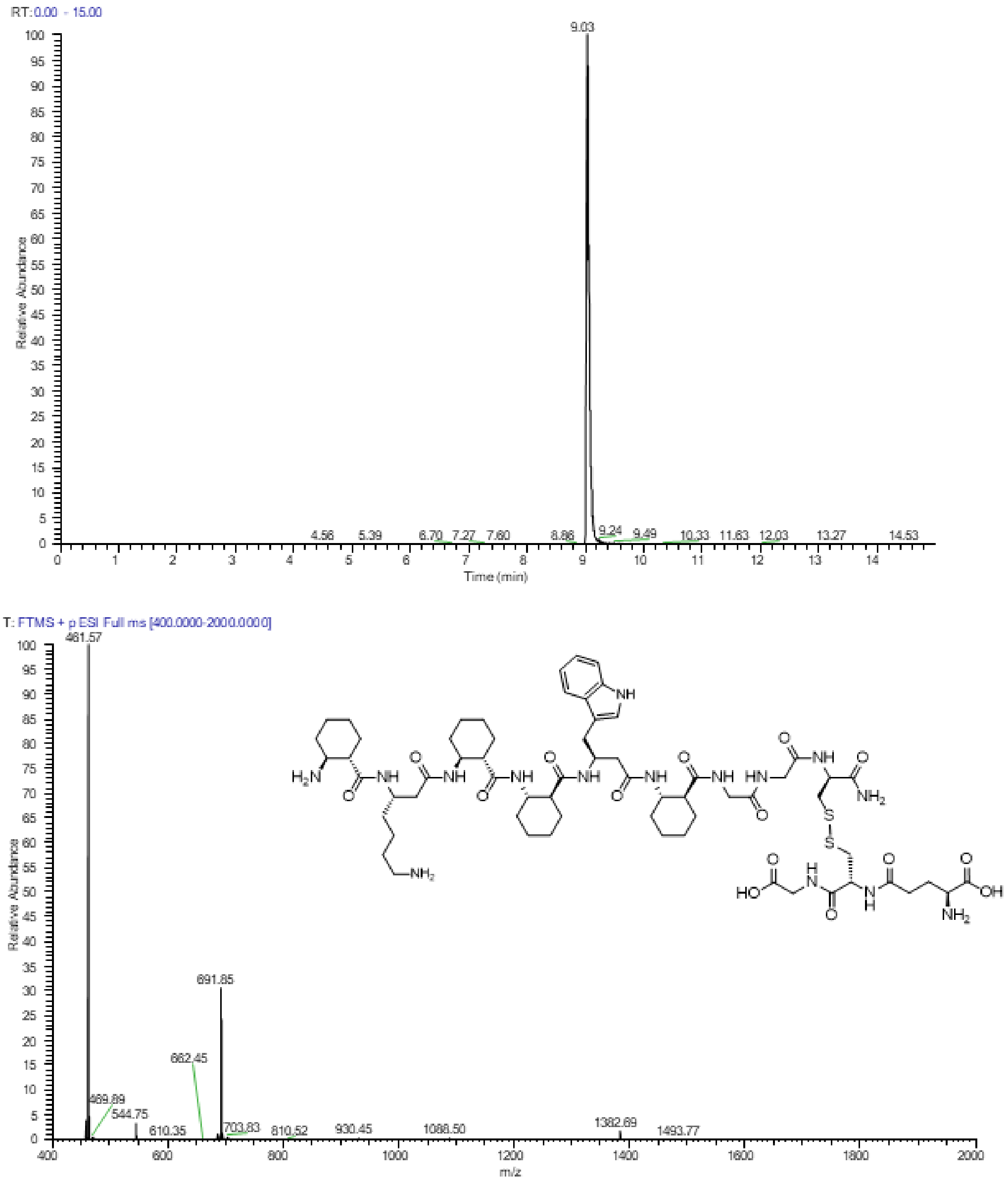

HPLC trace and mass spectrum of the glutathionic precursor **KW-G** (exact mass: 1381.69). Calculated isotopic profile for $[M+2H]^{2+}$ (species, abundance): 691.3477 (100%), 691.8494 (69.22%), 692.3447 (8.95%), 692.3479 (3.79%); m/z calculated: 1382.47 $[M+H]^{+}$, 691.74 $[M+2H]^{2+}$, 461.49 $[M+3H]^{3+}$; m/z observed: 1382.69 $[M+H]^{+}$, 691.85 $[M+2H]^{2+}$, 461.57 $[M+3H]^{3+}$

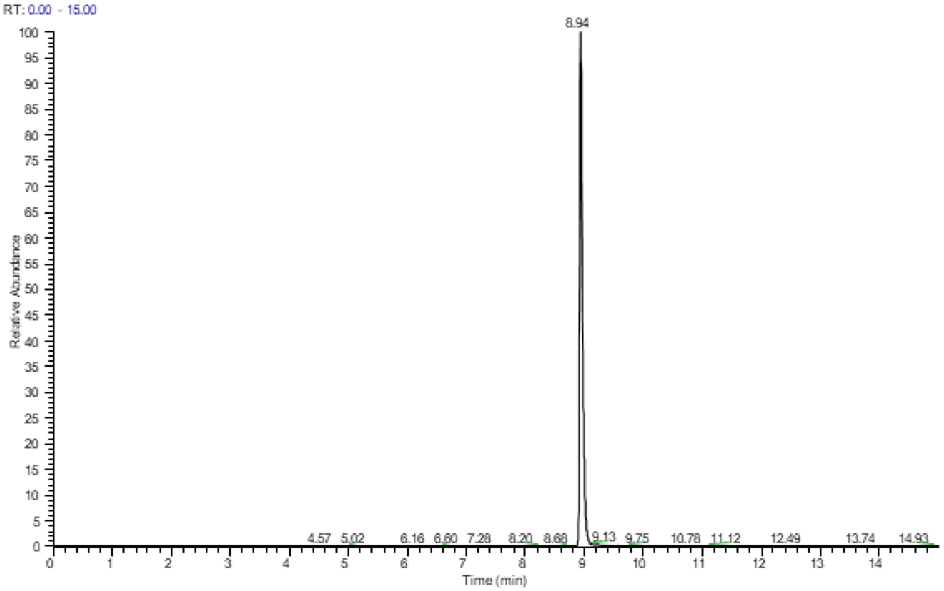

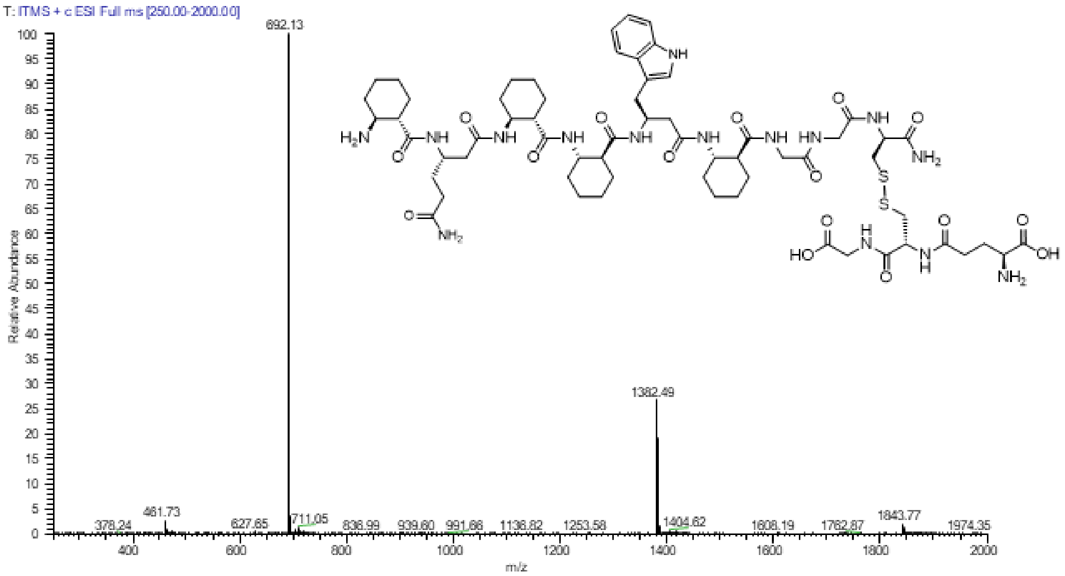

HPLC trace and mass spectrum of the glutathionic precursor **QW-G** (exact mass: 1381.65). Calculated isotopic profile for $[M+2H]^{2+}$ (species, abundance): 691.3295 (100%), 691.8312 (68.14%), 692.3274 (8.95%), 692.3297 (3.73%); m/z calculated: 1382.43 $[M+H]^{+}$, 691.72 $[M+2H]^{2+}$; m/z observed: 1382.49 $[M+H]^{+}$, 692.13 $[M+2H]^{2+}$

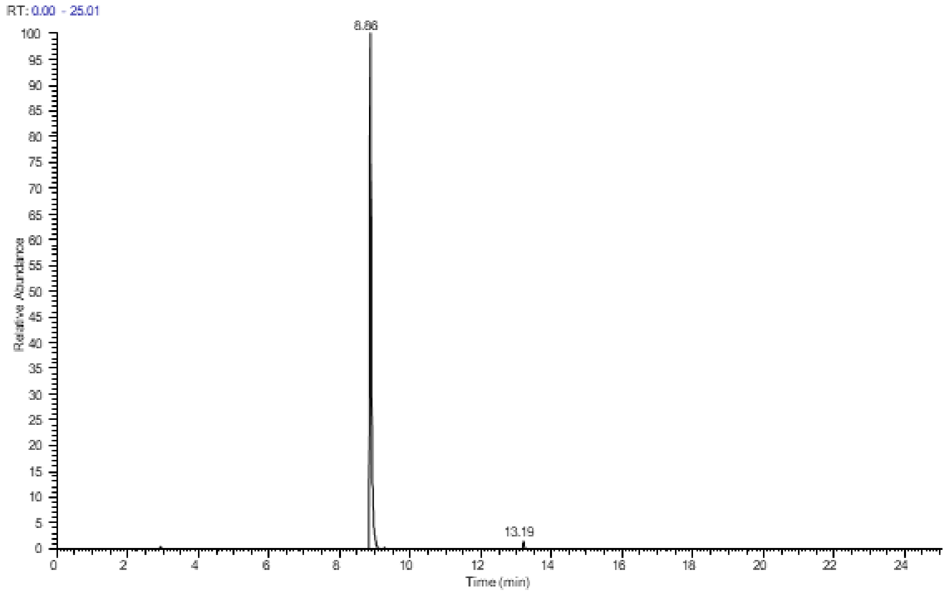

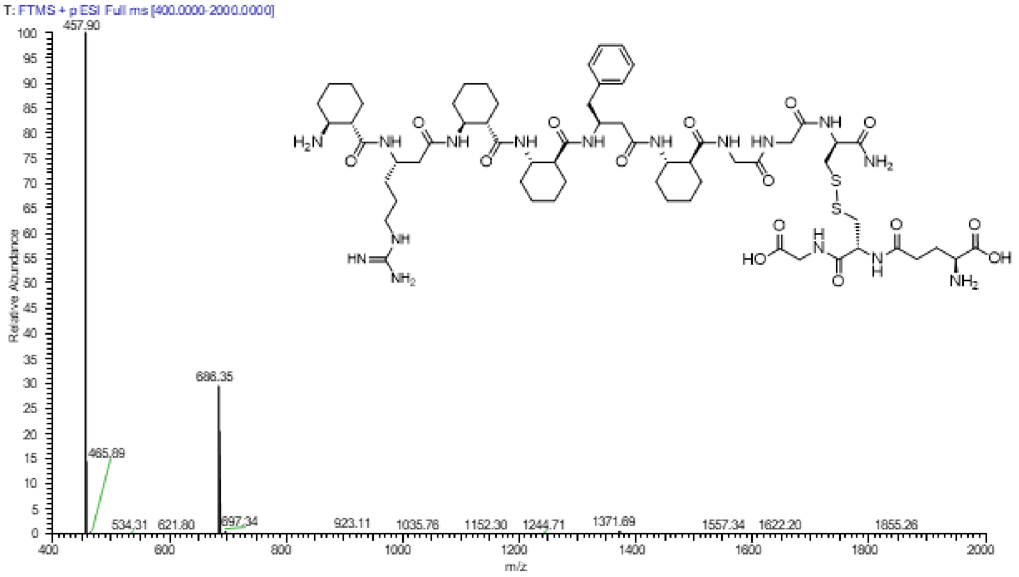

HPLC trace and mass spectrum of the glutathionic precursor **RF-G** (exact mass: 1370.68). Calculated isotopic profile for $[M+2H]^{2+}$ (species, abundance): 685.8453 (100%), 686.3470 (67.06%), 686.8432 (8.95%), 686.8455 (3.92%); m/z calculated: 1371.45 $[M+H]^{+}$, 686.23 $[M+2H]^{2+}$, 457.82 $[M+3H]^{3+}$; m/z observed: 1371.69 $[M+H]^{+}$, 686.35 $[M+2H]^{2+}$, 457.90 $[M+3H]^{3+}$

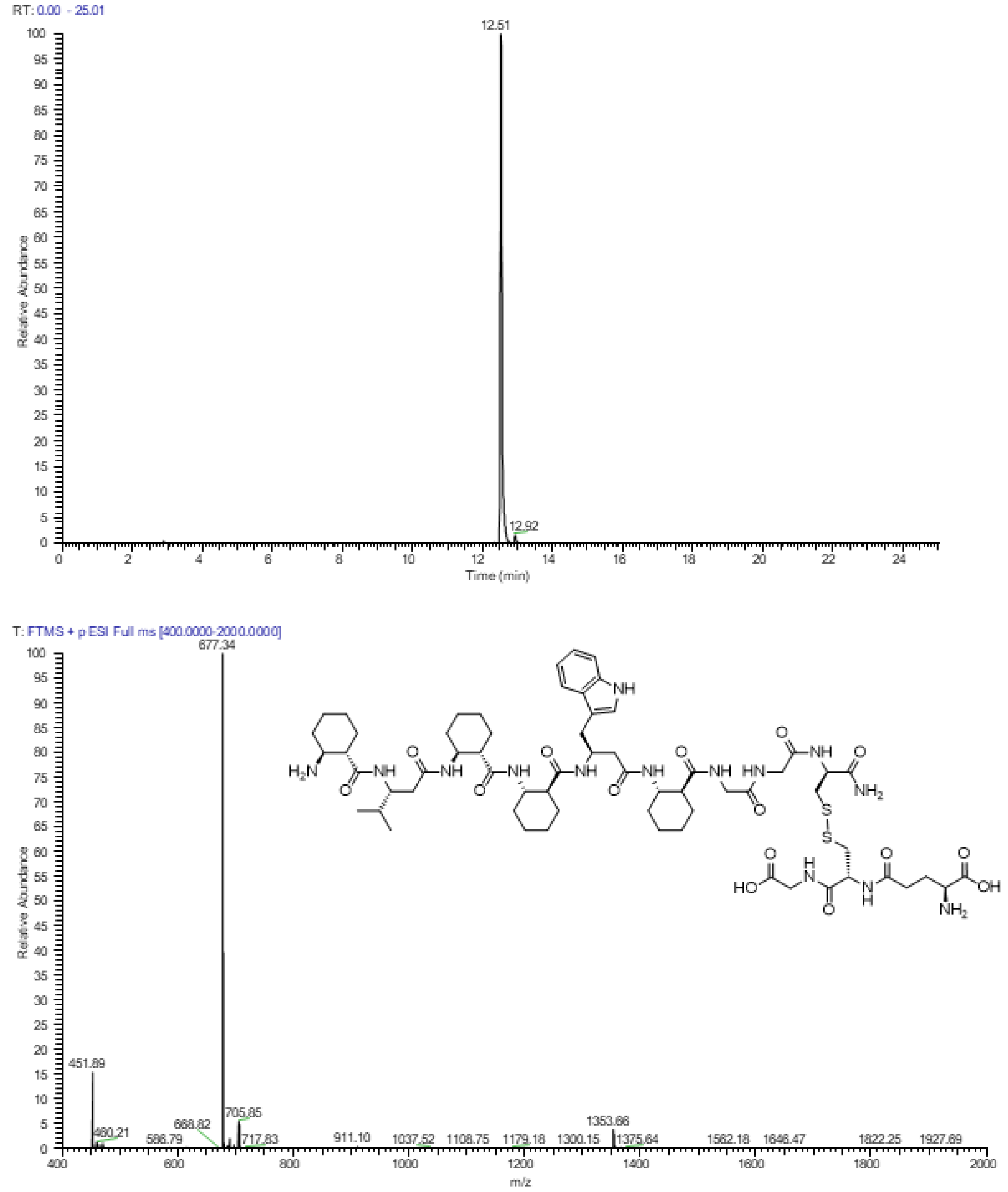

HPLC trace and mass spectrum of the glutathionic precursor **VW-G** (exact mass: 1352.66). Calculated isotopic profile for $[M+2H]^{2+}$ (species, abundance): 676.8344 (100%), 677.3361 (68.14%), 677.8323 (8.95%), 677.8346 (3.49%); m/z calculated: 1353.43 $[M+H]^{+}$, 677.22 $[M+2H]^{2+}$; m/z observed: 1353.66 $[M+H]^{+}$, 677.34 $[M+2H]^{2+}$

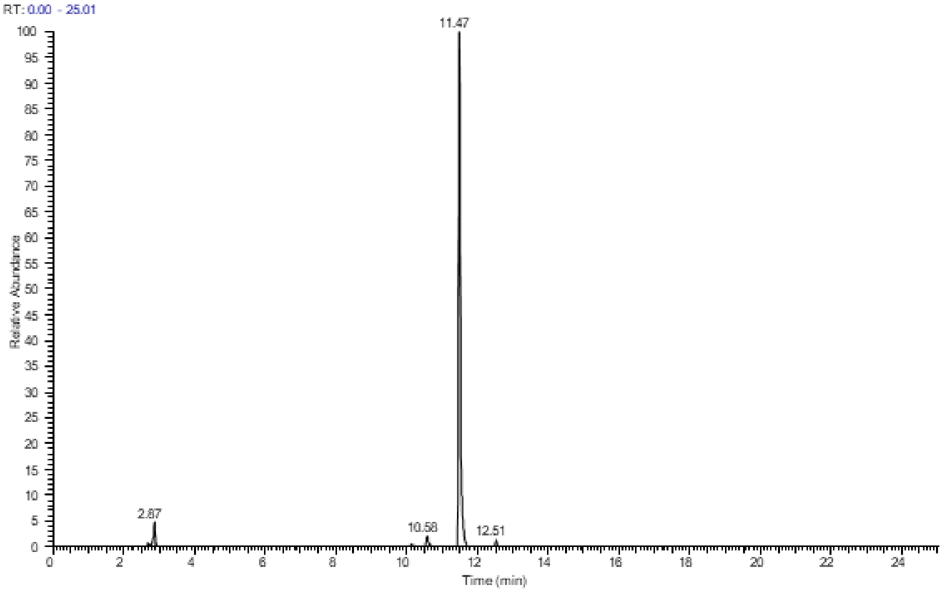

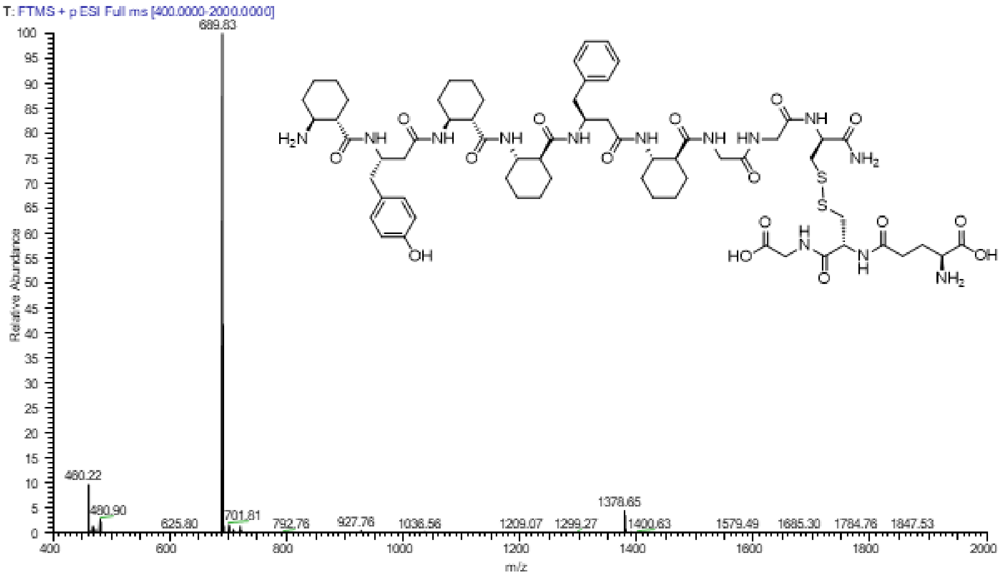

HPLC trace and mass spectrum of the glutathionic precursor **YF-G** (exact mass: 1377.65). Calculated isotopic profile for $[M+2H]^{2+}$ (species, abundance): 689.3264 (100%), 689.8281 (70.30%), 690.3243 (8.95%), 690.3266 (3.34%); m/z calculated: 1378.44 $[M+H]^{+}$, 689.72 $[M+2H]^{2+}$; m/z observed: 1378.65 $[M+H]^{+}$, 689.83 $[M+2H]^{2+}$

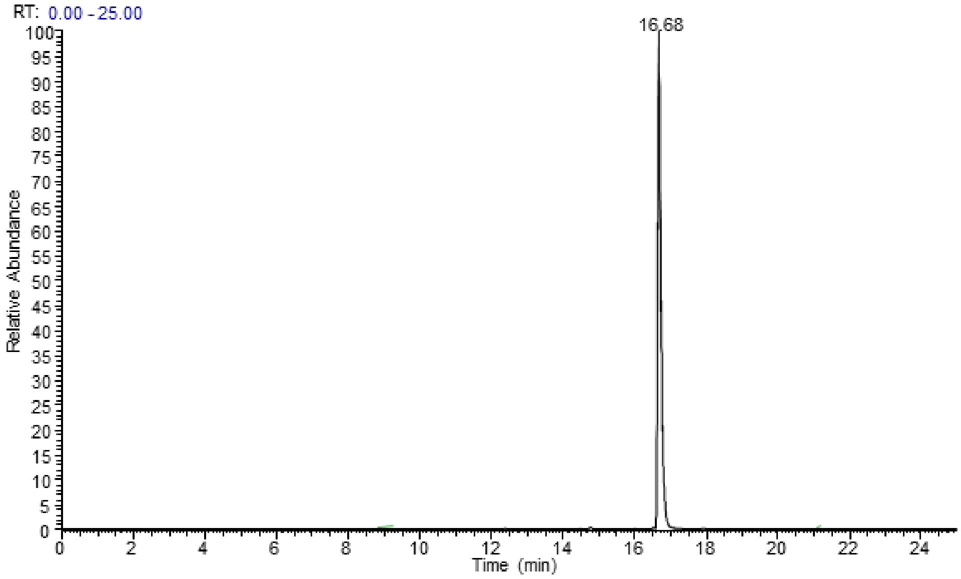

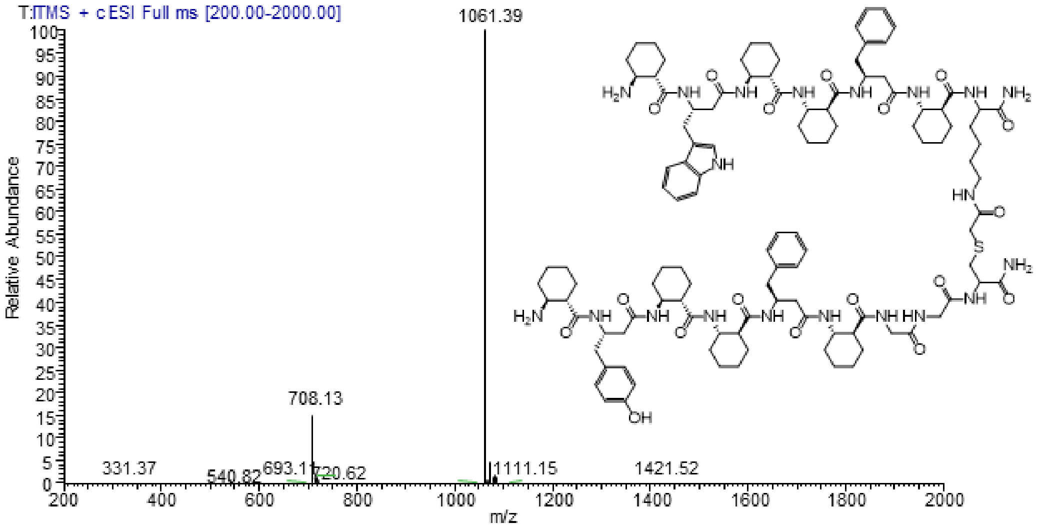

HPLC trace and mass spectrum of the glutathionic precursor **WF-S-YF** (exact mass: 2119.21). Calculated isotopic profile for $[M+2H]^{2+}$ (species, abundance): 1061.1138 (100%), 1060.6121 (81.82%), 1061.6123 (7.31%); m/z calculated: 2120.22 $[M+H]^{+}$, 1061.35 $[M+2H]^{2+}$; 707.90 $[M+3H]^{3+}$ m/z observed: 1061.39 $[M+2H]^{2+}$, 708.13 $[M+3H]^{3+}$

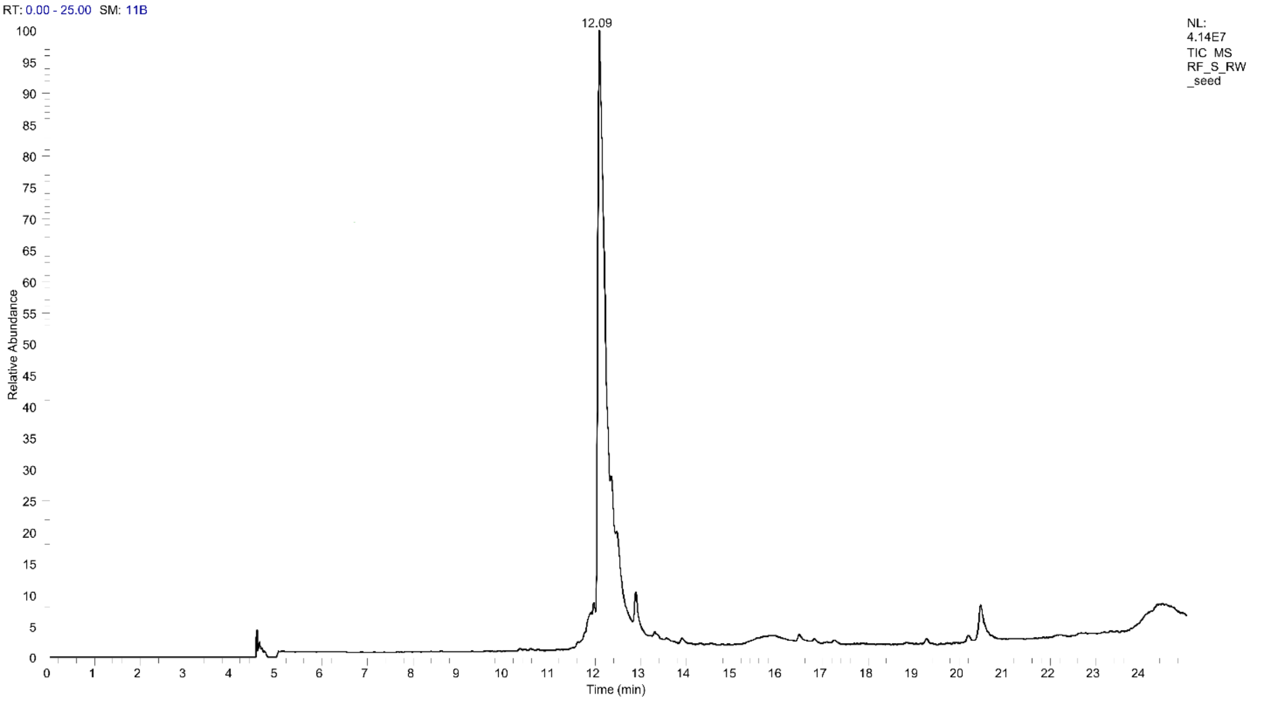

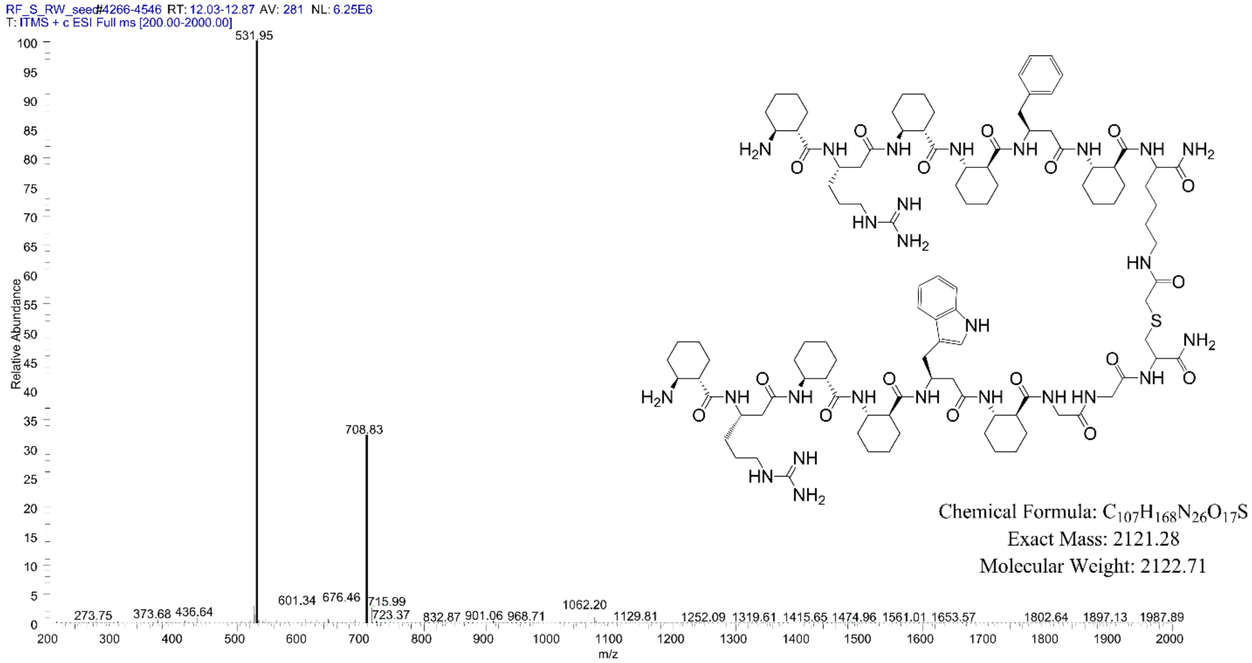

HPLC trace and mass spectrum of the glutathionic precursor **RF-S-RW** (exact mass: 2121.28). Calculated isotopic profile for $[M+2H]^{2+}$ (species, abundance): 1062.1488 (100%), 1060.6499 (70.03%), 1061.6519 (13.63%); m/z calculated: 2122.28 $[M+H]^{+}$, 1062.14 $[M+2H]^{2+}$; 708.10 $[M+3H]^{3+}$ m/z observed: 1062.20 $[M+2H]^{2+}$, 708.83 $[M+3H]^{3+}$, 531.95 $[M+4H]^{4+}$

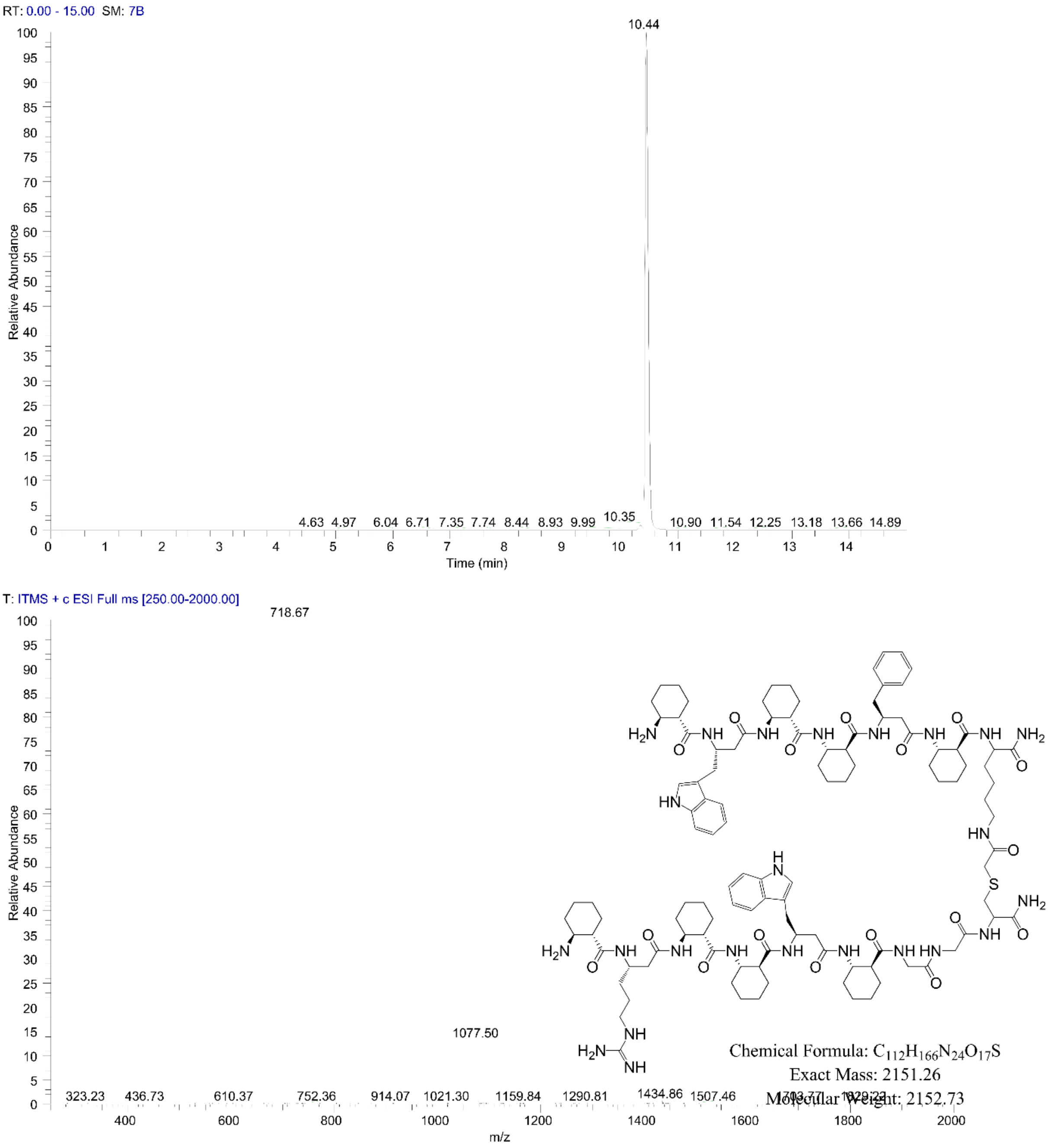

HPLC trace and mass spectrum of the glutathionic precursor **WF-S-RW** (exact mass: 2151.26). Calculated isotopic profile for $[M+2H]^{2+}$ (species, abundance): 1077.1381 (100%), 1077.6391 (72.14%), 1078.1402 (36.88%); m/z calculated: 2152.26 $[M+H]^{+}$, 1076.63 $[M+2H]^{2+}$; 718.09 $[M+3H]^{3+}$ m/z observed: 1077.50 $[M+2H]^{2+}$, 718.67 $[M+3H]^{3+}$

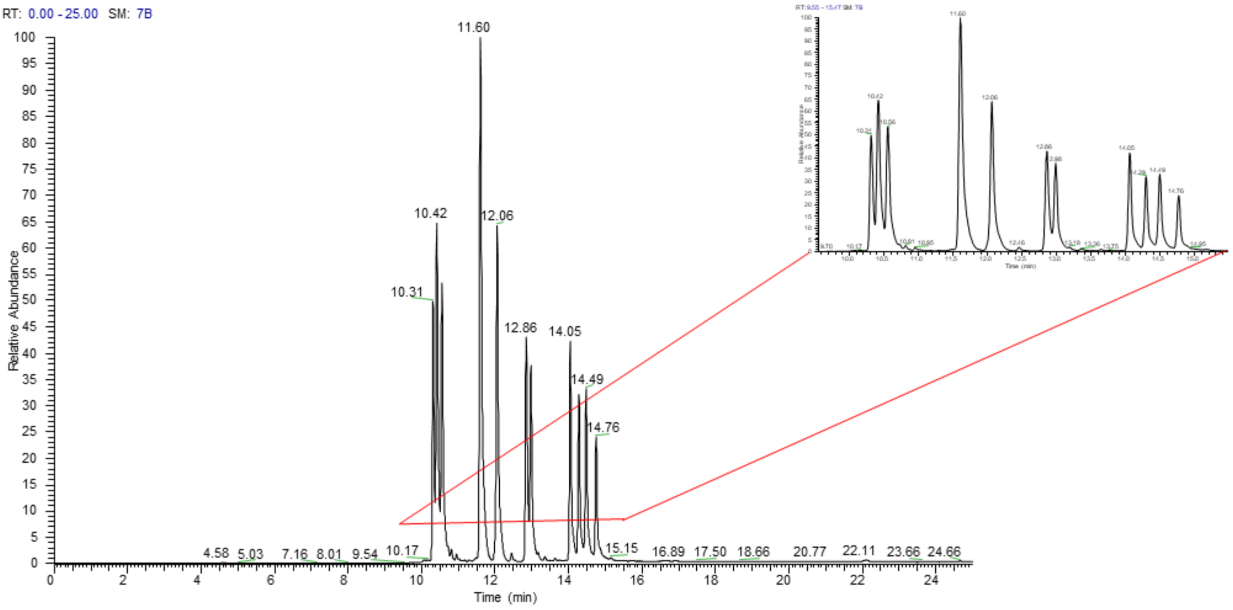

Characterisation of the monomer library. Total ion chromatogram of the initial foldamer library containing 12 different glutathione-protected monomers. Conditions of the analytical HPLC-MS measurement: Column: Aeris Widepore XB-C18 (250 x 4.6 mm) Method: 5–80% B during 25 minutes, flow rate: 0.7 mL min^{-1}, where eluent A: 0.1% HCOOH in water, eluent B: 0.1% HCOOH in ACN. For retention time and molar mass of the compounds, see Table S3.

References

[1] É. Bartus, Z. Hegedüs, E. Wéber, B. Csipak, G. Szakonyi, T. A. Martinek, *ChemistryOpen* **2017**, *6*, 236-241.

[2] A. R. K. Dinesh Gupta, *Can. J. Chem.* **1980**, *58*, 1350-1354.

[3] a) G. von Kiedrowski, in *Bioorganic Chemistry Frontiers* (Eds.: H. Dugas, F. P. Schmidtchen), Springer Berlin Heidelberg, Berlin, Heidelberg, **1993**, pp. 113-146; b) P. G. Higgs, *J. Mol. Evol.* **2017**, *84*, 225-235.
